# Simulation Predicts IGFBP2-HIF1α Interaction Drives Glioblastoma Growth

**DOI:** 10.1371/journal.pcbi.1004169

**Published:** 2015-04-17

**Authors:** Ka Wai Lin, Angela Liao, Amina A. Qutub

**Affiliations:** Department of Bioengineering, Rice University, Houston, Texas, United States of America; University of New Mexico, UNITED STATES

## Abstract

Tremendous strides have been made in improving patients’ survival from cancer with one glaring exception: brain cancer. Glioblastoma is the most common, aggressive and highly malignant type of primary brain tumor. The average overall survival remains less than 1 year. Notably, cancer patients with obesity and diabetes have worse outcomes and accelerated progression of glioblastoma. The root cause of this accelerated progression has been hypothesized to involve the insulin signaling pathway. However, while the process of invasive glioblastoma progression has been extensively studied macroscopically, it has not yet been well characterized with regards to intracellular insulin signaling. In this study we connect for the first time microscale insulin signaling activity with macroscale glioblastoma growth through the use of computational modeling. Results of the model suggest a novel observation: feedback from IGFBP2 to HIF1α is integral to the sustained growth of glioblastoma. Our study suggests that downstream signaling from IGFI to HIF1α, which has been the target of many insulin signaling drugs in clinical trials, plays a smaller role in overall tumor growth. These predictions strongly suggest redirecting the focus of glioma drug candidates on controlling the feedback between IGFBP2 and HIF1α.

## Author Summary

Current treatment for glioblastoma patients is limited to nonspecific methods: surgery followed by a combination of radio- and chemotherapy. With these methods, glioma patient survival is less than one year post-diagnosis. Targeting specific protein signaling pathways offers potentially more potent therapies. One promising potential target is the insulin signaling pathway, which is known to contribute to glioblastoma progression. However, drugs targeting this pathway have shown mixed results in clinical trials, and the detailed mechanisms of how the insulin signaling pathway promotes glioblastoma growth remain to be elucidated. Here, we developed a computational model of insulin signaling in glioblastoma in order to study this pathway’s role in tumor progression. Using the model, we systematically test contributions of different insulin signaling protein interactions on glioblastoma growth. Our model highlights a key driver for the growth of glioblastoma: IGFBP2-HIF1α feedback. This interaction provides a target that could open the door for new therapies in glioma and other solid tumors.

## Introduction

Glioblastoma is the most prevalent, highly malignant and aggressive type of primary brain tumor [1]. The current standard of care for glioblastoma patients includes concurrent radiation and chemotherapy using temozolomide after surgical removal of the tumor [2]. Though this treatment regime is aggressive, the effect on patient outcomes has been disappointing. Glioblastoma patient survival rate has stagnated for the past 30 years, with median survival time less than 1 year [3–5]. Only 20% of young (0–19 years old) glioma patients survive past 5 years, and this number drops to just over 5% for patients between 40 to 64 years old and to less than 5% for patients, 65 years old and older [1]. Such poor prognoses highlight the need for a new treatment strategy for glioblastoma patients.

Besides the attrition with age, reduced glioblastoma survival has also been independently linked to metabolic disorders. Previous studies showed that obese and diabetic patients with high grade glioblastoma have worse survival than their normal weight, non-diabetic counterparts [6–8]. Obesity is an established risk factor for type 2 diabetes, and like diabetes, obesity is associated with insulin resistance and hyperinsulinemia [9]. Due to these observations, an ongoing hypothesis is that aberrant insulin signaling accelerates glioblastoma progression, and that targeting this pathway may offer an alternative therapy to the current standard of care [10–12].

**Fig 1:**
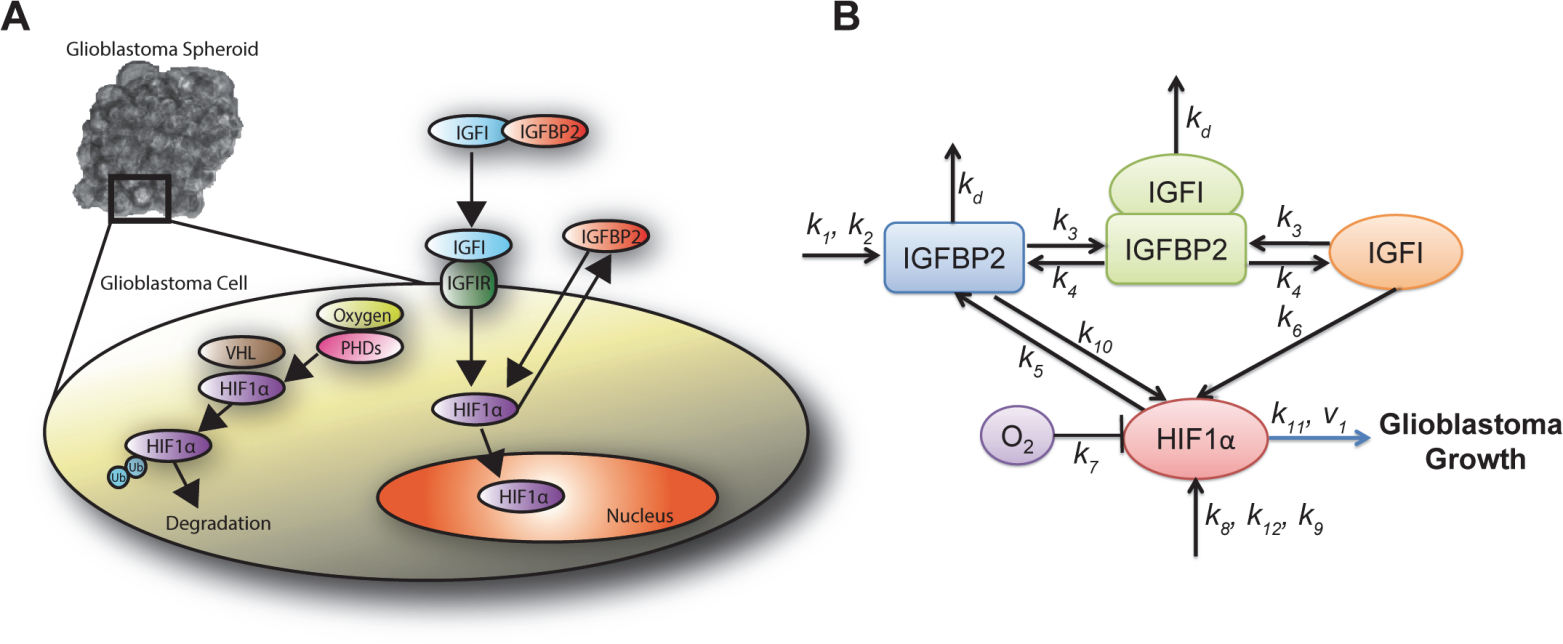
Insulin signaling in glioma. (A) Microscope image of glioma spheroids grown in vitro. Inset illustrates detailed intracellular insulin signaling. HIF1α = hypoxia-inducible factor 1 α, IGFBP2 = Insulin like growth factor binding protein 2, IGFI = Insulin like growth factor 1, IGFIR = Insulin like growth factor receptor 1, VHL = von Hippel-Lindau complex, PHDs = prolyl hydroxylase domain proteins and Ub = Ubiquitinated. (B) Schematic of the simplified insulin signaling pathway used in the computational model.

Key molecular players involved in this signaling have been identified (Fig 1), and extensively studied experimentally since the 1980s [13–18]. Insulin-like growth factor 1 (IGFI) and insulin-like growth factor 1 receptor (IGFIR) are an integral part of normal fetal and postnatal growth of the brain [19]. Brain cancer cells use the same pathways to develop into a cancerous phenotype [20]. Activation of IGFIR by IGFI and subsequent downstream signaling leads to malignant cell proliferation, motility and metastasis [21]. Consequently, researchers have targeted IGFIR to suppress glioblastoma growth. IGFIR inhibition has successfully reduced glioblastoma spheroid growth in vitro and in animal models [3, 22].

Unfortunately, the preclinical work has not successfully translated to clinical relevancy [23]. None of the IGFI-targeting drugs have passed phase III clinical trials [24]. This difficulty in obtaining clinical relevancy can be attributed to our limited understanding of the **how** and **why**: while key molecules have been identified, their dynamics have not been well studied. In order to treat glioma by targeting the insulin signaling pathway, the detailed molecular mechanisms linking this signaling pathway to cancer growth need to be understood. To that end, we developed a computational model that captures the dynamics of insulin signaling. We used our model to test the pathway’s role in glioma progression, with the broader goal of improving existing drug therapies and designing new strategies to treat glioma.

### Development of a computational model

A variety of multiscale modeling methods has been used to describe the growth of solid tumors, including both discrete and continuous approaches. A non-inclusive set of references include [25–29], along with several recent reviews [30–36]. Previous mathematical models of glioma progression have primarily focused on the growth or migration of cancerous cells from a tumor core [37–41]. Despite the increasing number and sophistication of the models, these studies have not considered insulin signaling. Conversely, computational models of insulin signaling exist [42, 43], but have only been applied to other applications, including articular cartilage [44], ovarian cancer [45], and human skeletal muscle [46], and exclude molecules of interest for brain cancer cells [44, 47]. Thus, we created for the first time, a computational chemical-kinetic model linking the insulin signaling pathway to glioblastoma growth.

#### Insulin pathway kinetics

A main goal of the modeling was to identify which sets of signaling regulators have the most influence on glioma progression. To do so, we first developed a theory of important molecular interactions based on the literature. We then designed in silico experiments to test their relative contribution to glioma progression. Fig 1A highlights intracellular insulin signaling pathways present in brain cancer cells. Insulin-like growth factor binding proteins (IGFBPs), which have a high affinity for IGFs, control IGF bioavailability. They can enhance or inhibit the actions of IGFs [20, 48]. IGFBP2 binding to IGFI reduces the concentration of free IGFI and limits its ability to bind to its receptor IGFIR. Thus, it would be expected that higher IGFBP2 levels would reduce IGFIR activation and attenuate downstream signaling—reducing cell growth. However, the presence of IGFBP2 has been shown to promote the development, progression and invasion of gliomas [12, 49]. Notably the expression of IGFBP2 is higher in patients with late stage glioma (known as glioblastoma multiforme), compared to those with earlier stages of the disease [50–53]. Furthermore, the silencing of IGFBP2 using short hairpin RNA (shRNA) has been shown to reduce the metastatic invasiveness in glioblastoma [54], a key hallmark of aggressive cancers. Thus, there exists a link between IGFBP2 and glioma cell growth independent of its effects through the binding of IGFI and the blocking of IGFIR activation.

We hypothesize that this correlation stems from IGFBP2 and its interaction with the transcription factor hypoxia inducible factor 1 α (HIF1α) (Fig 1A). HIF1α is an oxygen sensor which is continually produced in cells, and continually degraded if sufficient oxygen is present. Under normoxic conditions, VHL protein tags hydroxylated HIF1α for ubiquitination and subsequent degradation [55]. However, excess HIF1α that has not been degraded (in hypoxic conditions) is transported into the nucleus, where it binds to its dimer ARNT / HIF1β and subsequently upregulates other genes that promote cell growth [55]. HIF1α can also be regulated by oxygen-independent pathways, as known to be the case in cancer [56, 57]. Some roles for HIF1α in glioma progression and in the insulin signaling pathway specifically have been identified: HIF1α promotes malignant cell growth, and elevated expression of HIF1α has been strongly correlated to tumor malignancy [58–60]. IGFIR activation, through the binding of IGFI to IGFIR, triggers downstream signaling to HIF1α [61]. Moreover, one study discovered a reciprocal, positive relationship between IGF and HIF1α, with HIF1α upregulating mRNAs encoding for IGF2 and IGFBP, but not that of IGFI [62]. Supporting these studies, the inhibition of HIF1α through RNA interference results in a reduction of glioma growth [63]. While these key interactions have been established between molecular factors in the insulin signaling pathway, their dynamics have not been. Moreover, though glioma drug development has focused on IGFI signaling, it remains unproven which insulin signaling compound and its associated coregulators contribute to the greatest glioma progression. We explore for the first time here the multiple roles of IGFBP2 and IGFI, their complex interactions with HIF1α, and their importance in glioma progression.

In Materials and Methods, we describe the creation of a model of insulin signaling in glioma to encompass the aforementioned molecular interactions. We determined unknown model parameters by parameter fitting using both existing literature data and results from our own experiments on glioma spheroid growth. The computational model revealed how inhibition of specific molecular interactions in the insulin signaling pathway could lead to significant reduction of glioblastoma growth. In the Discussion, we describe how these results may be used to explain outcomes of IGFBP2-targeted clinical trials, and in the future, help inform the design of new therapies.

## Materials and Methods

### Insulin signaling interactions in glioblastoma

We developed a chemical-kinetic model that characterizes the network architecture and dynamics of the insulin signaling pathway—and then links these molecular interactions to cell and tissue level responses. Based on previous literature on the insulin signaling pathway, we constructed a model comprised of 4 differential equations and 1 mass conservation equation which describe interactions between components in the insulin signaling system (see Fig 1B). Our aim was to create the minimal model necessary to capture all the following interactions of key molecules:

#### IGFI

Once IGFI is bound to IGFBP2, IGFI becomes inactive and cannot bind to IGFIR or activate downstream signaling. IGFBP2 acts as reservoir for IGFI as it sequesters IGFI for release at a later time [64]. An increase in IGFI concentration leads to the activation of HIF1α through the RAS pathway [61]; and furthermore, it leads to increased production of HIF1α [61]. In our model, we have incorporated this by assuming IGFI directly promotes the production of HIF1α.

#### IGFBP2

In addition to the interactions with IGFI, IGFBP2 is involved in other pathways that are related to cancer progression independent of the IGF system. IGFBP2 was previously shown to interact with integrin alpha 5 [65], which further signals to Integrin Linked Kinase (ILK). The pathways related to ILK show that HIF1α is a downstream signal of ILK [66]. In our model, IGFBP2 was assumed to be promoted by HIF1α. Neither ILK, nor any other potential intermediate, is explicitly modeled.

#### HIF1α

The concentration of HIF1α in the nucleus depends on molecular factors that can be divided into two categories: oxygen dependent and oxygen independent. Oxygen independent interactions are interactions from IGFI and IGFBP2. Activation of IGFIR by IGFI binding to IGFIR leads to an increase in HIF1α levels via downstream signaling. HIF1α is constitutively expressed and is produced through an autocrine process which we assume is independent of oxygen concentration [67, 68]. Under normoxic conditions, HIF1α is readily degraded which results in no detectable cytosolic HIF1α levels [69]. Oxygen binds to prolyl hydroxylase domain proteins (PHDs), which activates the PHDs to hydroxylate HIF1α. The hydroxylated regions of HIF1α bind to von Hippel-Lindau (pVHL) ubiquitin E3 ligase complex which will then ubiquitinate the HIF1α complex, marking it for degradation by the proteasome. Under hypoxic conditions, the lack of oxygen does not allow for the hydroxylation of HIF1α. Consequently HIF1α is not ubiquitinated or degraded, leading to an accumulation of HIF1α and its entry into the nucleus, where it binds HIF1β and activates downstream genes. Since the degradation of HIF1α depends on PHDs and the production of PHDs depends on HIF1α, in our model, we assume the degradation of HIF1α depends on both HIF1α and oxygen levels.

### Model equations

#### Insulin-like Growth Factor 1 (IGFI)

$${\left[IGFI\right]}_{total}={\left[IGFI\right]}_{free}+{\left[IGFI-IGFBP2\right]}_{complex}$$ 1

Total concentration of IGFI = Concentration of free IGFI + Bound (IGFI-IGFBP2)_complex_.

#### Insulin-like Growth Factor Binding Protein 2 (IGFBP2)

$$\begin{array}{l} \frac{d\left[IGFBP2\right]}{dt}=k_{1}\left[IGFBP2\right]\left[IGFI\right]\left(1-k_{2}\left[IGFBP2\right]\right)-k_{3}\left[IGFI\right]\left[IGFBP2\right]+\\ k_{4}{\left[IGFI-IGFBP2\right]}_{complex}-k_{d}\left[IGFBP2\right]+k_{5}\left[HIF1\alpha \right] \end{array}$$ 2

Rate of change in IGFBP2 = production of IGFBP2—binding of IGFBP2 to IGFI + dissociation of (IGFI-IGFBP2)_complex_—degradation of IGFBP2 + upregulation of HIF1α.

#### Bound complex of Insulin-like Growth Factor 1 and Insulin-like Growth Factor Binding Protein 2 (IGFI-IGFBP2)_complex_

$$\begin{array}{l} \frac{d{\left[IGFI-IGFBP2\right]}_{complex}}{dt}=k_{3}\left[IGFI\right]\left[IGFBP2\right]-k_{3}{\left[IGFI-IGFBP2\right]}_{complex}\\ -k_{d}{\left[IGFI-IGFBP2\right]}_{complex} \end{array}$$ 3

Rate of change in (IGFI-IGFBP2)_complex_ = formation of (IGFI-IGFBP2)_complex_—dissociation of (IGFI-IGFBP2)_complex_—degradation of (IGFI-IGFBP2)_complex_.

#### Hypoxic Inducible Factor 1 alpha (HIF1α)

$$\frac{d\left[HIF1\alpha \right]}{dt}=k_{6}\left[IGFI\right]-k_{7}{\left[O_{2}\right]}_{cons\tan t}\left[HIF1\alpha \right]+\frac{k_{8}\left[HIF1\alpha \right]}{k_{12}+k_{9}\left[HIF1\alpha \right]}+k_{10}\left[IGFBP2\right]$$ 4

Rate of change in HIF1α = production of HIF1α due to activation of IGFI—degradation of HIF1α by oxygen + production of HIF1α in absence of oxygen + production of HIF1α due to activation of IGFBP2.

#### Glioblastoma Diameter (GD)

$$\frac{d\left[GD\right]}{dt}=v_{1}+k_{11}\left[HIF1\alpha \right]$$ 5

Rate of change in GD = diameter change due to basal glucose dependent growth + diameter change due to HIF1α dependent growth.

$$\text{Glioblastoma Volume}=\frac{4}{3}\pi {\left(\frac{\text{Glioblastoma Diameter}}{2}\right)}^{3}.$$ 6

### Growth of glioblastoma experiments

The growth rate of the glioblastoma tumor, Eq 5, was determined by regression analysis using the data from both our previous experiments on spheroid growth in vitro using the U87 glioblastoma cell line and LN229 glioblastoma growth in mice [70]. The U87 and LN229 glioblastoma cell lines were used to compare glioblastoma cell lines which were more dependent on insulin signaling (LN229) and less dependent on insulin signaling (U87) [3]. Growth of the glioblastoma is normally measured experimentally by changes in the volume or the diameter of the cancerous spheroid/tumor mass. In the model, glioblastoma growth is a time-varying function, defined as net growth of the glioblastoma spheroid/tumor volume and is assumed to depend on its basal growth and the additional growth that is promoted by HIF1α.

#### In vitro hanging drop assay

**Fig 2:**
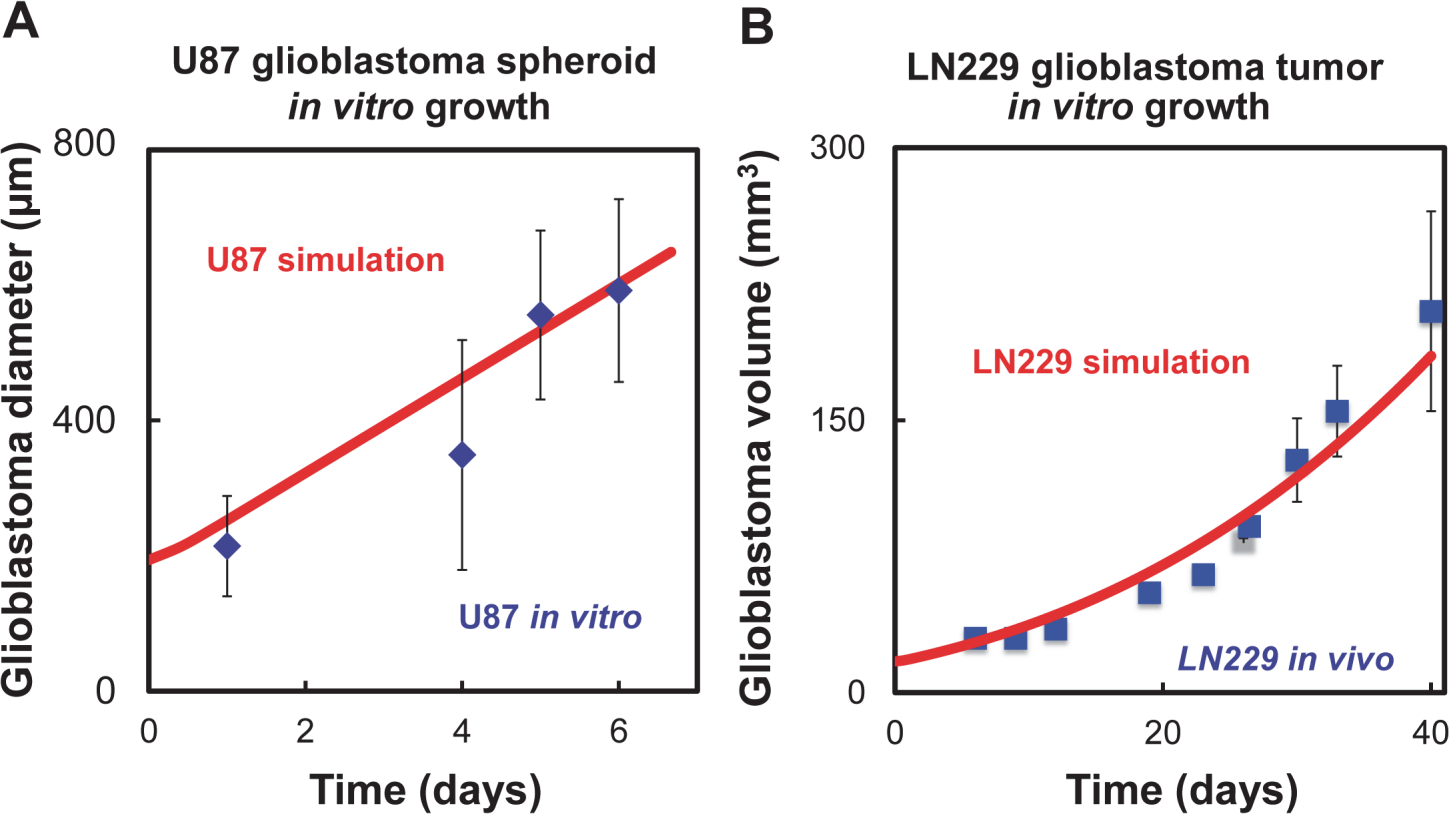
Computational model results compared to glioblastoma growth data. (A) In vitro data using U87 cell line, R^2^ = 0.86. (B) In vivo data using LN229 cell line, R^2^ = 0.95. Blue points represent in vitro experiments and red lines represent the computational simulations.

We performed the following in vitro assay in order to form glioblastoma spheroids and track their growth: U87 cells were collected from cells plated on tissue culture flasks, and the cells were suspended to a final concentration of 45,000 cells/mL using Lonza DMEM media with 5% methocel. The cell suspensions were plated as droplets on 60 mm petri dish lids. Each plate lid contained approximately 20 droplets of 20 μl cell suspension. The lids were then inverted over a petri dish bottom containing 2 ml of PBS to keep the media from evaporating. The inverted droplets were kept in an incubator at 37°C with 5% CO_2_. By observing the spheroids using phase contrast imaging (Ti-E Nikon automated stage microscope system), we measured the minor and major axes of the spheroid diameters on days 1, 4, 5, and 6 after they had been seeded. The average of these measurements are displayed (Fig 2A).

#### In vivo

In vivo glioma progression was based on data obtained from Fig 4E of ref [70]. The experiment recorded growth of LN229 tumors derived from cells transduced with lentiviruses expressing a scrambled short hairpin RNA (shRNA). The glioblastoma volume data represents the average of ten mice.

#### Oxygen levels

Oxygen levels in the in vitro hanging drop experiments are kept constant, and we assumed uniform oxygen levels in the media. The oxygen level in the model is set at the start of a simulation with any value between the range of 2% and 21%. The oxygen level is then held constant for the duration of a particular simulation.

### Fitting model parameters

**Fig 3:**
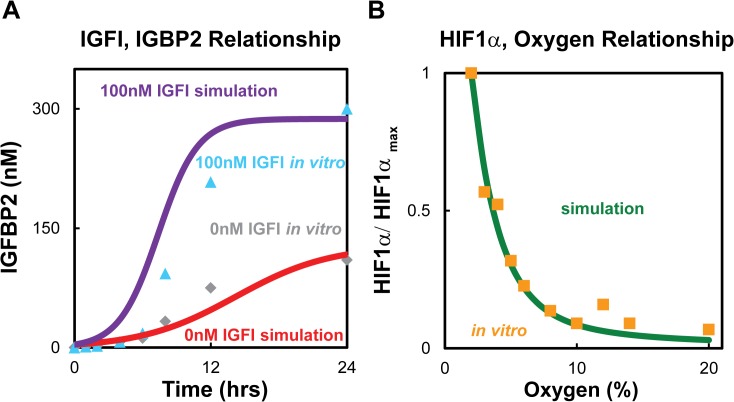
Comparisons between computational model and literature data. (A) Relationship between IGFB2 and IGFI over time. Grey and blue points represent in vitro data obtained from Slomiany et al [74] where 0 nM and 100 nM of IGFI, were added to the media at the start of the experiment respectively. Red line represents computational simulation with no added IGFI, R^2^ = 0.91, and purple line indicates computational simulation with 100 nM of IGFI added at 0 hrs, R^2^ = 0.83. (B) HIF1α as a function of O_2._ Orange points are the in vitro expression data obtained in HeLa cells. Green line shows model simulations using the same initial conditions as the in vitro experiments, R^2^ = 0.97.

A genetic algorithm was used to determine default values for the unknown kinetic rates (the genetic algorithm was employed in Matlab, and refined using *fminsearch*). The estimated initial conditions and fitted rate constants are shown in Tables 1 and 2. The model was fitted for three outputs: glioblastoma growth rate; HIF1α vs. O_2_ levels; and IGFI as a function of IGFBP2. The glioblastoma growth rates were found for two distinct experiments (U87 and LN229) by fitting the same model and obtaining different initial conditions and growth rates for the two cell lines. Results from fitting the in vitro U87 spheroid growth and literature data of LN229 growth in mice are shown in Fig 2A and 2B, respectively. HIF1α is a function of oxygen levels, and it was fitted using data from Jiang et al. [71] which monitored how the HIF1α levels changed in HeLa cells as a function of O_2_. The rate constants were simultaneously fitted using data of IGFI and IGFBP2 levels as a function of each other and time (see Fig 3A, Slomiany et al. [41]). In those experiments, the IGFBP2 concentration was monitored as a function of time under two external concentrations of IGFI (0 nM and 100 nM). The experiments used the human retinal pigment epithelial (RPE) cell line D407; and it is an assumption of the model that the same relationships hold in glioma cells (these measurements are the only ones we are aware of that measure IGFBP2 as a function of IGFI levels). We also estimated that the IGFBP2 response was the same as that of IGFBP3, which is the IGFBP species available from the in vitro experimental data. Initial conditions were also determined from experiments. The concentration of IGFI under normal conditions was calculated based on the data by Lonn et al [72]. Similarly the mean concentration of IGFBP2 in patients with glioblastoma was calculated from a previous study [73]. Both of the calculations for IGFI and IGFBP2 are shown in the S1 File.

**Table 1:** Initial conditions used in the model.

Species	Name	Initial value (U87)	Initial value (LN229)	Unit
[IGFI]	Insulin-like growth factor I	92.5	92.5	nM
[IGFBP2]	Insulin-like growth factor binding protein 2	3.68	3.68	nM
[IGFI-IGFBP2]_complex_	Bound IGFI and IGFBP2	2.6	2.6	nM
[HIF1α]	Hypoxic inducible factor 1 alpha	1	1	μM
[GD]	Glioblastoma diameter	170	3200	μm

**Table 2:** Rate constants used in computational model.

Constant	Description	Value (U87)	Value (LN229)	Units	Source
*k*_1_	Production rate of IGFBP2	0.0452	0.0452	hr/nM	Fit from [74]
*k*_2_	Production rate of IGFBP2	0.0004	0.0004	1/nM	Fit from [74]
*k*_3_	Binding rate of IGFBP2 and IGFI	0.0002	0.0002	hr/nM	Fit from [74]
*k*_4_	Dissociation rate of complex	0.0007	0.0007	1/hr	Fit from [74]
*k*_5_	Promotion of IGFBP2 feedback by HIF1α	9.5495	9.5495	1/hr	Fit from [74]
*k*_6_	Production rate of HIF1α by IGFI	0.0001	0.0001	1/nM	Fit from [74]
*k*_7_	Degradation rate of HIF1α by oxygen	0.1176	0.1176	1/hr	Fit from [74]
*k*_8_	Hill equation rate constant	0.01057	0.01057	1/hr	Fit from [71]
*k*_9_	Hill equation rate constant	0.0241	0.0241	1/μM	Fit from [71]
*k*_10_	Promotion of HIF1α by IGFBP2	0.0002	0.0002	1/hr	Sensitivity analysis
*k*_*d*_	Degradation rate of IGFBP2	3.92702	3.92702	1/hr	Fit from [74]
*v*_1_	Basal growth of glioma based on glucose	2.601	0.5779	μm/hr	Fit from [54]
*k*_11_	Growth rate due to HIF1α	21.6	179.55	μm/hr	Fit from [54]
*k*_12_	Hill equation rate constant	21	21	(dimensionless)	Fit from [54]

### Sensitivity analysis

**Fig 4:**
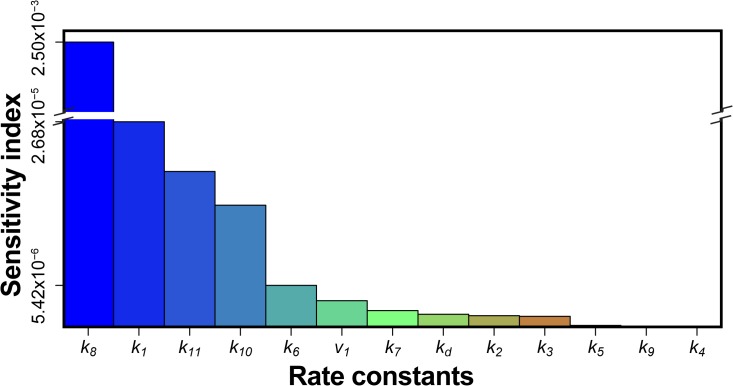
Results of model sensitivity to single rate constants as measured by the sensitivity index. Sensitivity index of each model parameter as defined in the main text. The sensitivity index is shown in descending order going from left to right. Rate constant *k*_8_ (production of HIF1α) had the highest sensitivity when varying the rate constants individually.

Initial concentrations of all molecular factors involved in the system were varied independently between 0.1×-10× of the fitted concentrations, and the effect on each compound and overall glioma growth was simulated. Oxygen levels were tested between 2–21%. The sensitivity of glioblastoma growth to changes in kinetic rate constants was determined for kinetic rates of 0.1×-10× the fitted values individually. The results from the complete sensitivity analysis can be found in S2 File. Sensitivity analysis was summarized by calculating the sensitivity index (see below) at 40 days for the LN229 cell line in Table 1. The time duration of 40 days was chosen as it matched the duration of studies performed in the in vivo LN229 work from literature. The following equation was used to calculate the sensitivity index to quantify the levels of sensitivity. The sensitivity index was plotted in Fig 4. The definitions of each variable in the sensitivity index can be found in Table 3.

**Table 3:** Description of variables in sensitivity index.

Variable	Description
*GD*_*n*_	Glioblastoma diameter at final time point using varied rate constant
*GD*_*o*_	Glioblastoma diameter at final time point at optimized value
*C*	Maximum glioblastoma diameter at final time point.
*T*	Time duration of simulation
Δ*k*	Multiplying factor by which rate constant was varied

### Sensitivity index

$$\text{Sensitivity Index}=\frac{\left|GD_{n}-GD_{o}\right|}{CT\Delta k}.$$ 7

### Global sensitivity analysis

**Fig 5:**
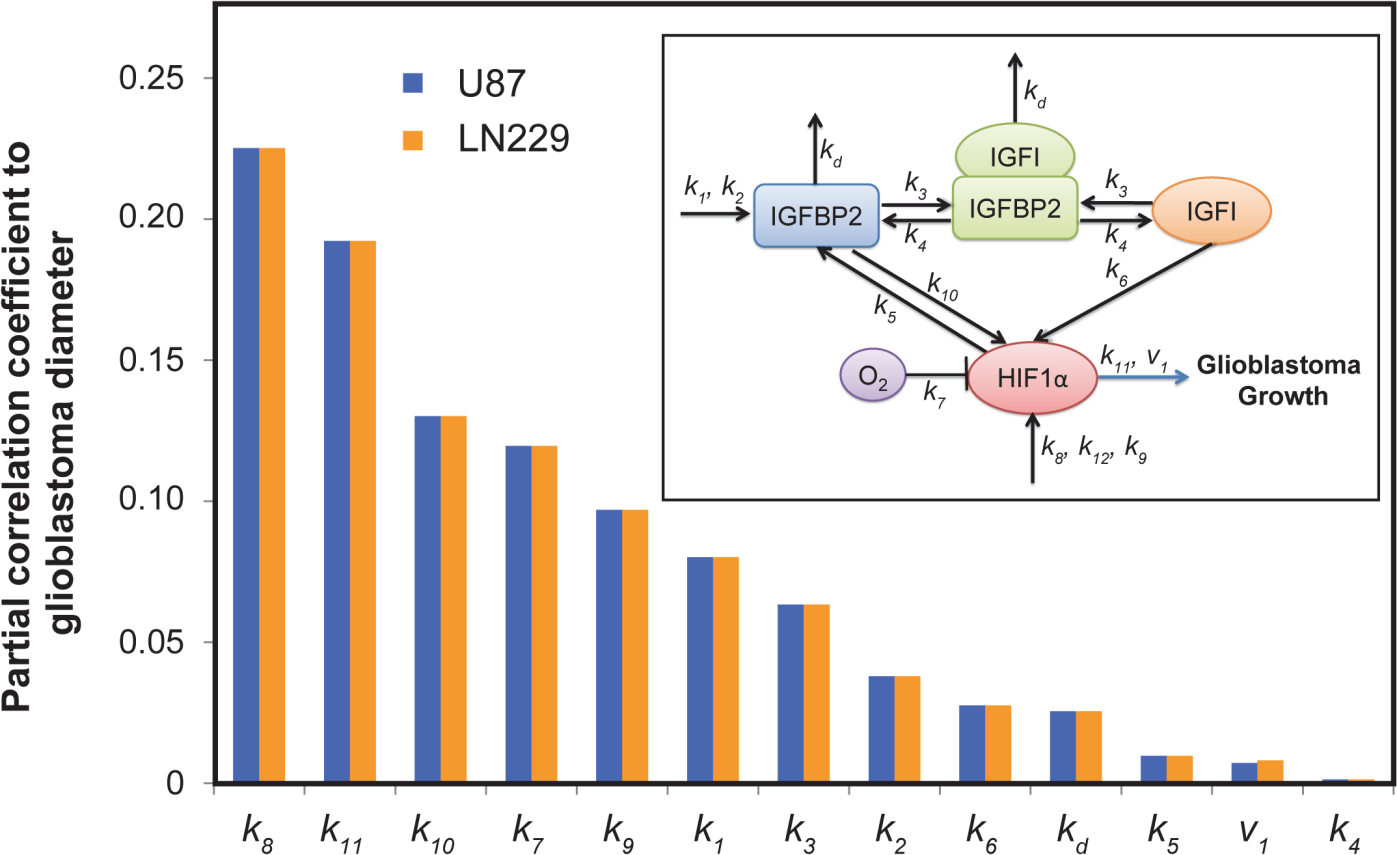
Partial Correlation of rate constants to glioblastoma growth. Latin Hypercube sampling of all rate constants within 0.1× to 10× of fitted values followed by Partial Correlation of rate constants to glioblastoma growth. Blue bars represent U87 cell line while orange bars represent LN229 cell line. Rate constant *k*_8_ (production of HIF1α) had the highest sensitivity when varying the rate constants in combination using the Latin Hypercube sampling method. Inset shows the schematic of the simplified insulin signaling pathway used in the computational model.

In addition to varying the rate constants individually, we simultaneously explored the entire parameter space of the rate constants (varying between 0.1×–10× of the fitted values) using the Latin Hypercube Sampling method [75]. From this sampling, 500 sets of rate constants were simulated in the model for glioma growth over 40 days where the glioblastoma diameter was recorded. Principal component analysis illustrating the resulting glioblastoma diameters as a function of multi-varied kinetics rates is shown in S1–S4 Figs. Additionally, to confirm the kinetic parameters that most significantly influence glioma progression, glioblastoma diameters were correlated to the rate constants by calculating partial correlations (Fig 5).

### Glioblastoma growth reduction

**Fig 6:**
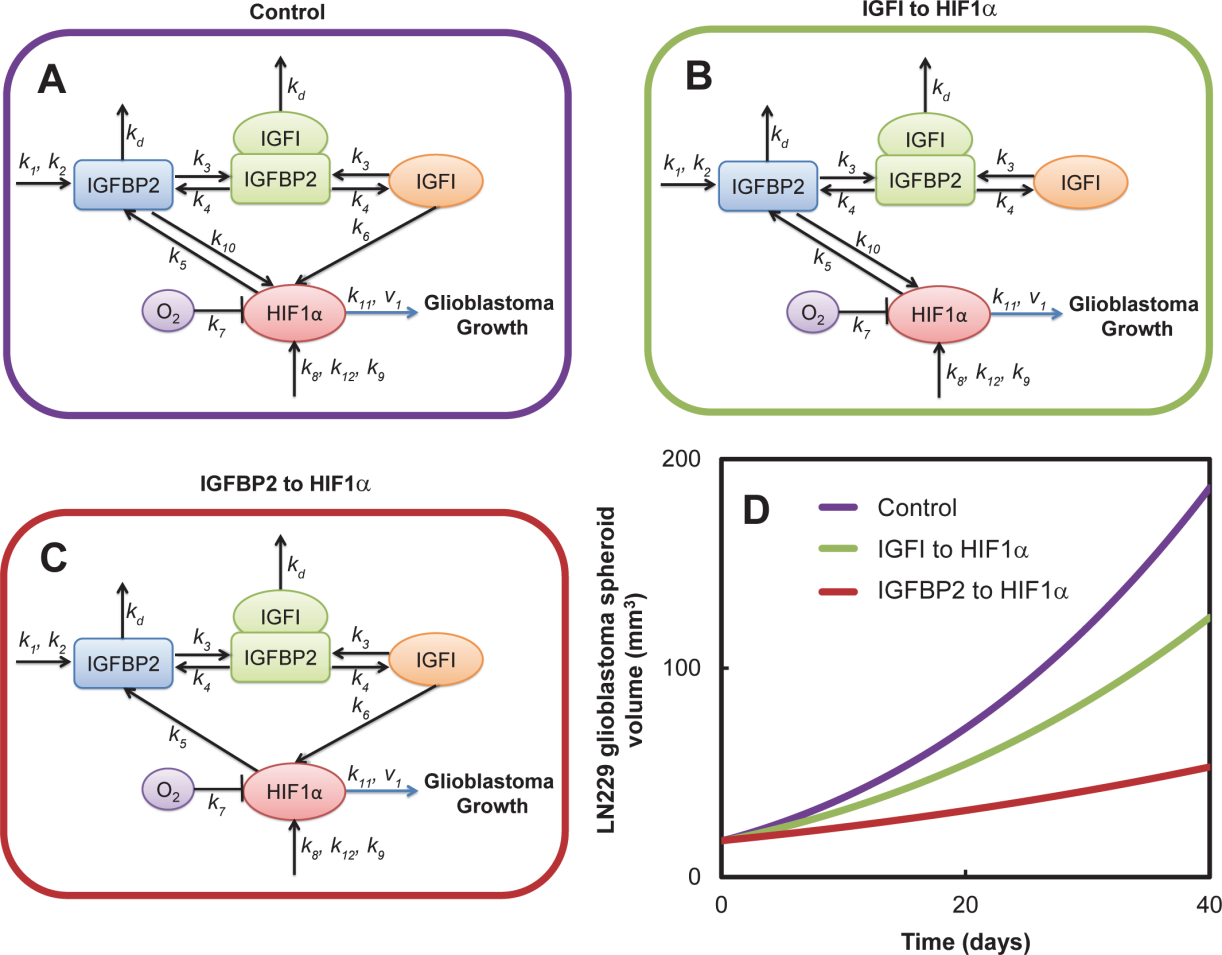
In silico reduction of glioblastoma growth for LN229 glioblastoma cell line. Glioblastoma growth was simulated for (A) control conditions, and when two separate interactions were removed from the model: (B) IGFI to HIF1α and (C) IGFBP2 to HIF1α. (D) Removal of the IGFBP2 to HIF1α interaction had the greatest reduction in the glioblastoma growth as compared to control conditions.

To simulate the effect of using different drug targeting factors in glioblastoma, we set each rate constant to 0 separately, modeling the effects of removing each interaction, with the exception of the basal production and degradation of HIF1α. The exception is because HIF1α is ubiquitous in cells; targeting HIF1α would not only affect glioblastoma cells but also other cells. Setting the rate constant to 0 simulated the removal of each reaction from the system. The diameter of the glioblastoma for both cell lines U87 and LN229 was then compared to the original pathway before the removal of the reaction. The glioblastoma diameter was simulated over 40 days. Results are shown in Fig 6.

### Parameter fitting

Unknown rate constants were found by fitting existing literature data. Fig 4A shows the model simulations compared to the literature in vitro data, to which the model was fit, that monitored the IGFBP2 concentration as a function of time under two external concentrations of IGFI (0 nM and 100 nM) in the system [74]. For the case with 0 nM external IGFI, the model simulations that best fitted the in vitro data was found to be internal IGFI concentration levels of 92.5 nM of IGFI. Fig 3B shows the model simulations compared to literature in vitro data that monitored HIF1α as a function of oxygen [71].

## Results

### Compounds that drive insulin signaling

**Fig 7:**
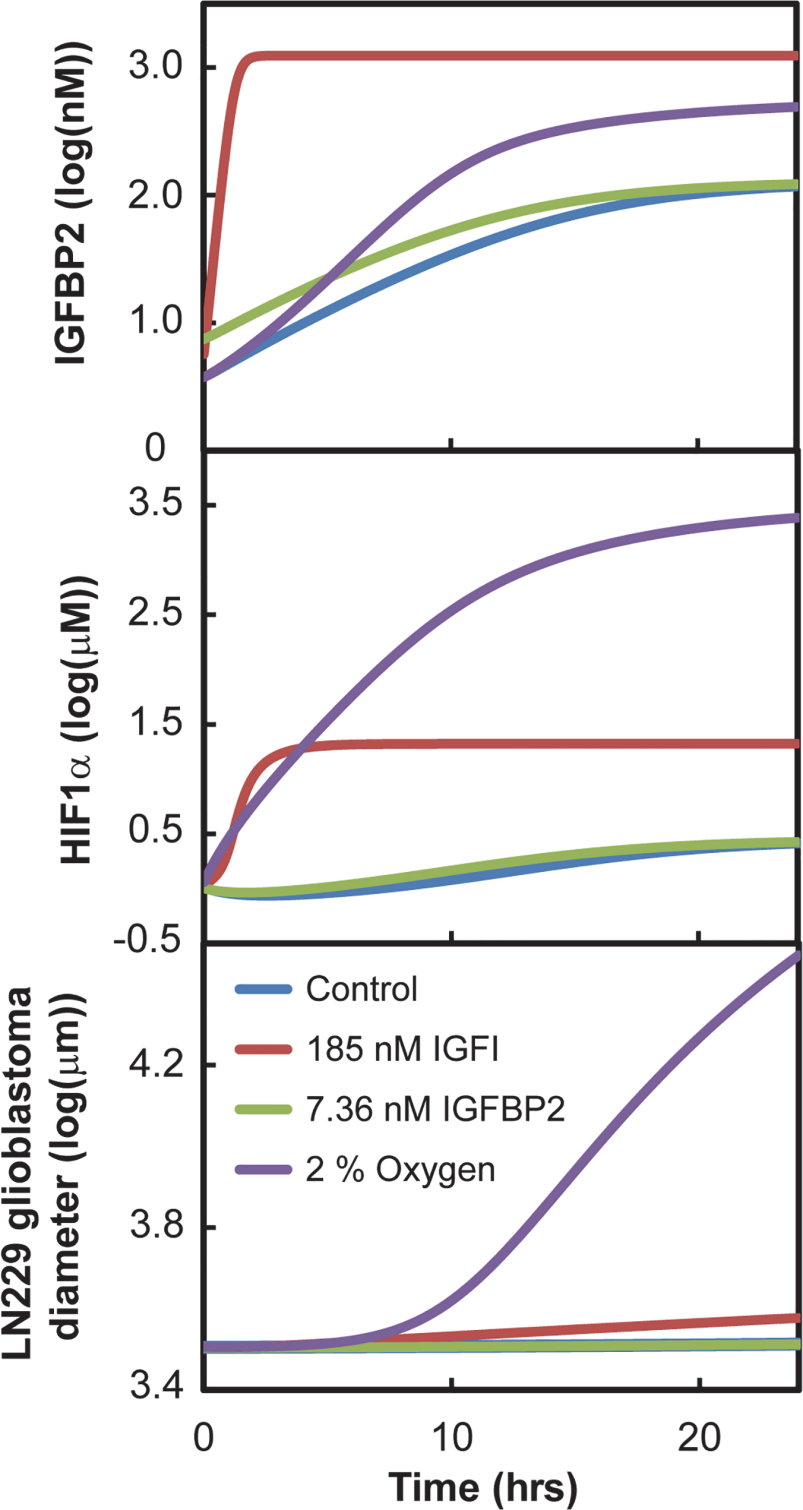
Effects of initial conditions on LN229 simulations. (A) IGFBP2 concentrations over time. (B) HIF1α concentrations over time. (C) LN229 glioblastoma diameter over time. Low oxygen conditions had the greatest increase in the growth of glioblastoma as compared to control.

Results of the sensitivity analysis on the initial model conditions showed that HIF1α and IGFBP2 levels in the insulin signaling system were most sensitive to reduced oxygen (2%) and also elevated IGFI_total_ levels (Fig 7). At higher concentrations of IGFI_total_, elevated steady state concentrations of IGFI and IGFBP2 were observed. In hypoxic conditions (2% oxygen), HIF1α and IGFBP2 concentrations were increased initially and reached a steady-state of 7× and 1.25× baseline values, respectively.

Varying initial conditions in the model showed that the insulin system is highly sensitive to reduced oxygen concentrations and elevated IGFI concentrations compared to the default initial conditions (control). For the remaining initial conditions, the insulin signaling system in glioblastoma was robust over changes in initial HIF1α concentrations and the (IGFI-IGFBP2) complex concentration.

### Insulin signaling pathway reactions that drive glioma growth

In order to analyze the contribution of each rate constant to glioblastoma growth, the sensitivity index was calculated for each rate constant, for LN229 tumor growth (shown in Table 4 in descending order). Results are plotted in Fig 4, which shows that LN229 glioblastoma growth was most sensitive to the production of HIF1α (*k*_8_) production of IGFBP2 (*k*_1_), growth rate due to HIF1α (*k*_11_) and promotion of HIF1α by IGFBP2 (*k*_10_). Results of the Latin Hypercube Sampling confirmed these findings. After computing the Partial Correlation Coefficients between rate constants and glioblastoma growth, we found that the production of HIF1α (*k*_8_) was the highest correlated rate constant to glioblastoma growth, as shown in Fig 6.

**Table 4:** Rate constants that glioblastoma growth rate were most sensitive to in LN229 cells.

Rate Constant	Description	Sensitivity Index (*hrs* ^−1^)
*k*_8_	Hill equation rate constant	2.50×10^−3^
*k*_1_	Production rate of IGFBP2	2.68×10^−5^
*k*_11_	Growth rate due to HIF1α	2.03×10^−5^
*k*_10_	Promotion of HIF1α by IGFBP2	1.59×10^−5^
*k*_6_	Production rate of HIF1α by IGFI	5.42×10^−6^
*v*_1_	Basal growth of glioblastoma based on glucose	3.42×10^−6^
*k*_7_	Degradation rate of HIF1α by oxygen	2.13×10^−6^
*k*_*d*_	Degradation rate of IGFBP2	1.65×10^−6^
*k*_2_	Logistic equation rate of IGFBP2	1.46×10^−6^
*k*_3_	Binding rate of IGFBP2 and IGFI	1.38×10^−6^
*k*_5_	Promotion of IGFBP2 feedback by HIF1α	1.88×10^−7^
*k*_9_	Hill equation rate constant	4.75×10^−8^
*k*_4_	Dissociation rate of complex	4.96×10^−10^

Shown in descending order of sensitivity according to the sensitivity index at 40 days where each rate constant was varied 10× of fitted conditions.

When we removed each reaction independently from the model, the results were striking. When the feedback from IGFBP2 to HIF1α was removed in LN229 cells, the glioblastoma volume over the simulation of 40 days was halved as compared to when the downstream signal from IGFI to HIF1α was removed shown in Fig 6. Removal of the HIF1α to IGFBP2 connection had minimal effect on the glioblastoma growth. When a similar simulation was conducted for the U87 cell line, there was not a significant change in the glioblastoma volume when either the IGFBP2 to HIF1α or the IGFI to HIF1α connection was removed, see S5 Fig.

## Discussion

We have developed a chemical-kinetic model that predicts glioblastoma growth as a function of insulin signaling. Our model agrees with experimental in vitro data on interactions between IGFI, IGFBP2 and HIF1α. Sensitivity analysis on initial conditions found the insulin signaling pathway to be most sensitive to IGFI concentration and oxygen levels.

Current literature data on the relationship between HIF1α and oxygen shows that glioblastoma growth is insensitive to high oxygen levels, but highly sensitive at low oxygen concentrations. This is significant as glioblastoma spheroids are generally under hypoxic conditions. There is maximal HIF1α expression at low oxygen levels [76]. In addition, there are more pronounced changes in HIF1α expression at these low oxygen levels. Small changes in oxygen levels result in large changes in HIF1α levels. As the oxygen levels increase towards 21%, HIF1α levels are exponentially decreased. This relationship explains how glioblastoma tumors have a fairly constant response at higher oxygen levels. However, at low oxygen levels, glioblastoma will have drastically higher HIF1α levels which result in a much different phenotype and growth rate.

Drugs have been developed to target the IGFIR pathway by suppressing the IGFI to HIF1α pathway using three main types of compounds: IGFIR targeting antibodies, tyrosine kinase inhibitors for kinase domains of IGFIR, and IGFI ligand neutralizing antibodies [24, 77–79]. However, these compounds have failed to control glioblastoma growth clinically, and have not made it past phase III clinical trials [24].

Our sensitivity analysis on the rate constants showed that the contribution of basal HIF1α production to LN229 glioblastoma growth is greater than contribution of the IGFI-dependent HIF1α production. This suggests that HIF1α would be a more effective target to reduce glioblastoma growth than targeting the IGFIR molecular interactions by current drugs.

In fact, the top four rate constants that glioblastoma growth was most sensitive to when individually perturbed were the production of HIF1α (*k*_8_), production of IGFBP2 (*k*_1_), growth rate due to HIF1α (*k*_11_) and promotion of HIF1α by IGFBP2 (*k*_10_). However, since the HIF1α effects are ubiquitous in all cells, alterations in HIF1α and production of IGFBP2 would be difficult to target in cancerous cells only. On the other hand, IGFBP2 overexpression is specific to glioblastoma multiforme compared to gliomas. Thus we focused on the effect of promotion of HIF1α by IGFBP2 (*k*_10_), which had the third highest correlation found by Partial Correlation to glioblastoma growth in Fig 5. Our results from the growth reduction analysis showed that glioblastoma growth was more sensitive to the removal of feedback from IGFBP2 to HIF1α as compared to the IGFI to HIF1α interaction. There have not been any published drugs that have specifically blocked feedback between IGFBP2 and HIF1α in glioblastoma. Our model predicts that this pathway could result in significantly reduced growth of glioblastoma and should be targeted by the next generation of glioblastoma drugs.

This study offers an explanation for the difficulties encountered by current drugs targeting IGFIR to reduce glioblastoma cell growth: a secondary mechanism that upregulates HIF1α. We found that glioblastoma growth was highly sensitive to this new hypothesized interaction, IGFBP2 to HIF1α signaling. While other researchers have highlighted the importance of IGFBP2 in glioblastoma growth [80], we have been able to suggest a specific mechanism that can be potentially targeted. In our predictions, removing the feedback from IGFBP2 to HIF1α resulted in almost half of the growth in the glioblastoma diameter over 40 days as compared to removing the downstream signal from IGFI to HIF1α.

By using two different glioblastoma cell lines in our analysis, we have found that glioblastoma growth through the insulin signaling pathway is tumor specific. When we conducted the glioblastoma growth reduction analyses of the LN229 and U87 cell lines, there was almost no change in growth observed in the U87 cell lines, while the LN229 showed a reduction in the glioblastoma tumors’ growth. Glioblastoma cells lines that rely on the insulin signaling pathway for their aggressive growth phenotype will be more affected by drugs that target the insulin signaling pathway. Conversely, if the glioblastoma cells do not rely on the signaling from insulin for their growth, then targeting the insulin signaling pathway would not be effective in controlling the growth. This explains why when U87 and LN229 were targeted using TAE226 (IGFIR tyrosine kinase inhibitor), a larger amount of apoptosis was observed for the LN229 cell line compared to the U87 cells [3]. Thus, targeting the insulin signaling pathway through the IGFBP2-HIF1α interaction could be effective for those glioblastoma cells dependent on insulin signaling. Compensatory pathways may also influence cancer growth, and the computational results presented here warrant targeted experimental testing focusing on the IGFBP2-HIF1α interaction in the context of other signaling networks.

In conclusion, we have been able to achieve a deeper understanding of the interactions between key factors in the insulin signaling pathway through our computational model. The model allowed us to simulate the effects of removing different reactions in the insulin signaling pathway network, to test in silico potential therapeutic targets. These model predictions provide the impetus for future experimental studies exploring the role of IGFBP2-HIF1α interactions. In sum, we have found a possible target in the insulin signaling system that merits exploration as a candidate drug target for glioblastoma patients and other patients with cancers sensitive to the insulin signaling pathway.

## Supporting Information

S1 File Calculations for IGFI and IGFBP2. (PDF)

S2 File Complete sensitivity analysis results. Sensitivity analysis of initial conditions and rate constants on IGFI, IGFBP2, HIF1α and glioblastoma diameter for both U87 and LN229 glioblastoma cell lines for 24 hour simulation.
(PDF)

S1 Fig Results from Principle Component Analysis of the rate constants and its effect on the glioblastoma growth in the U87 glioblastoma cell line. PC1 is the first principle component and PC2 is the second principle component. Both components contributed about 10% each to the overall correlation.
(PDF)

S2 Fig Results from Principle Component Analysis of the rate constants and its effect on the glioblastoma growth in the U87 glioblastoma cell line. PC2 is the second principle component and PC3 is the third principle component. Both components contributed about 10% and 9% each to the overall correlation respectively.
(PDF)

S3 Fig Results from Principle Component Analysis of the rate constants and its effect on the glioblastoma growth in the LN229 glioblastoma cell line. PC1 is the first principle component and PC2 is the second principle component. Both components contributed about 10% each to the overall correlation.
(PDF)

S4 Fig Results from Principle Component Analysis of the rate constants and its effect on the glioblastoma growth in the LN229 glioblastoma cell line. PC2 is the second principle component and PC3 is the third principle component. Both components contributed about 10% and 9% each to the overall correlation respectively.
(PDF)

S5 Fig In silico reduction of glioblastoma growth for U87 glioblastoma cell line. Glioblastoma growth was simulated for (A) control conditions, and when two separate interactions were removed from the model: (B) IGFI to HIF1α and (C) IGFBP2 to HIF1α. (D) Removal of the IGFBP2 to HIF1α had a small reduction in the glioblastoma growth as compared to control conditions.
(PDF)
