# Supplementary material for: Simulation Predicts IGFBP2-HIF1α Interaction Drives Glioblastoma Growth
Source: PLoS Comput Biol. 2015 Apr 17;11(4):e1004169. doi: 10.1371/journal.pcbi.1004169 (PMC4401766; doi:10.1371/journal.pcbi.1004169)
Supplement: S1 File — (PDF) [file pcbi.1004169.s001.pdf]

S1 File: Calculations for IGFI and IGFBP2

The concentration of IGFI under normal conditions without the addition of IGFI was made through a conversion using the molecular weight of IGFI as 21841Daltons and the conversion factor of 1Da to 1g/mol. This value was based off a concentration value in Lonn et al [72] which looked at the histopathology of gliomas and stated the IGFI concentration was found to be 127ng/mL.

$$\frac{127\text{ng}}{1\text{ml}} \times \frac{1}{21841\text{Da}} \times \frac{1\text{Da(mol)}}{1\text{g}} \times \frac{1000\text{ml}}{1\text{L}} = \frac{5.81\text{nmol}}{\text{L}} = 5.81\text{nM}$$

The mean concentration of IGFBP2 in patients with glioma was 128ng/ml in a previous study [73]. The conversion to nM was made using the molecular weight of IGFP2 as 34814 Daltons and the conversion factor of 1Da to 1g/mol.

$$\frac{128\text{ng}}{1\text{ml}} \times \frac{1}{34814\text{Da}} \times \frac{1\text{Da(mol)}}{1\text{g}} \times \frac{1000\text{ml}}{1\text{L}} = \frac{3.68\text{nmol}}{\text{L}} = 3.68\text{nM}$$
