# Supplementary material for: Simulation Predicts IGFBP2-HIF1α Interaction Drives Glioblastoma Growth
Source: PLoS Comput Biol. 2015 Apr 17;11(4):e1004169. doi: 10.1371/journal.pcbi.1004169 (PMC4401766; doi:10.1371/journal.pcbi.1004169)
Supplement: S2 File — Sensitivity analysis of initial conditions and rate constants on IGFI, IGFBP2, HIF1α and glioblastoma diameter for both U87 and LN229 glioblastoma cell lines for 24 hour simulation. (PDF) [file pcbi.1004169.s002.pdf]

S2 File: Sensitivity analysis supplement

Sensitivity: initial conditions

IGFI

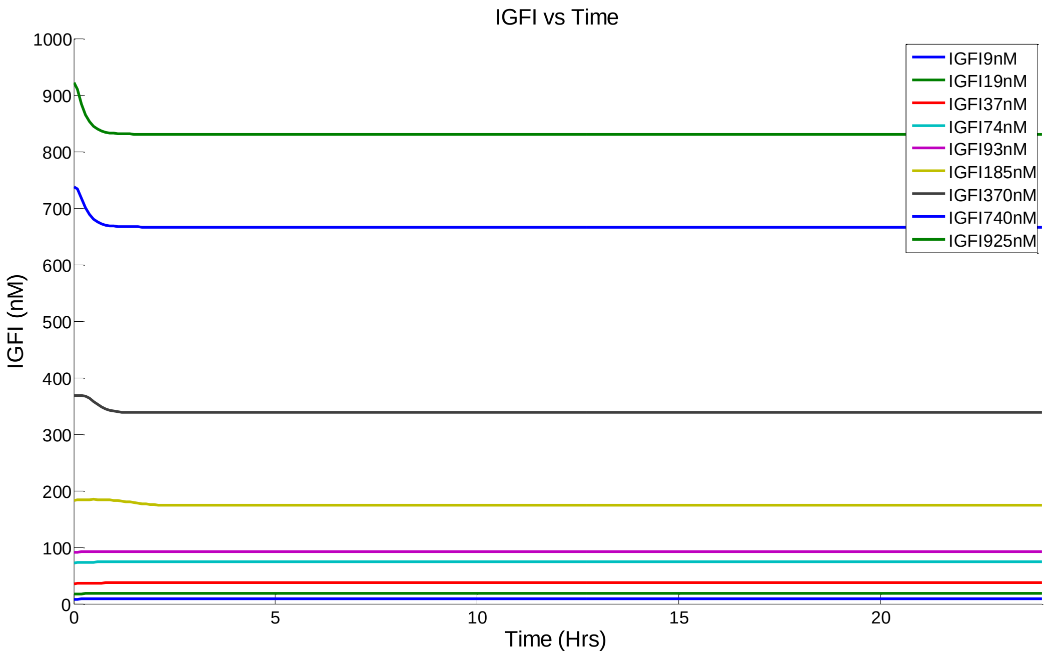

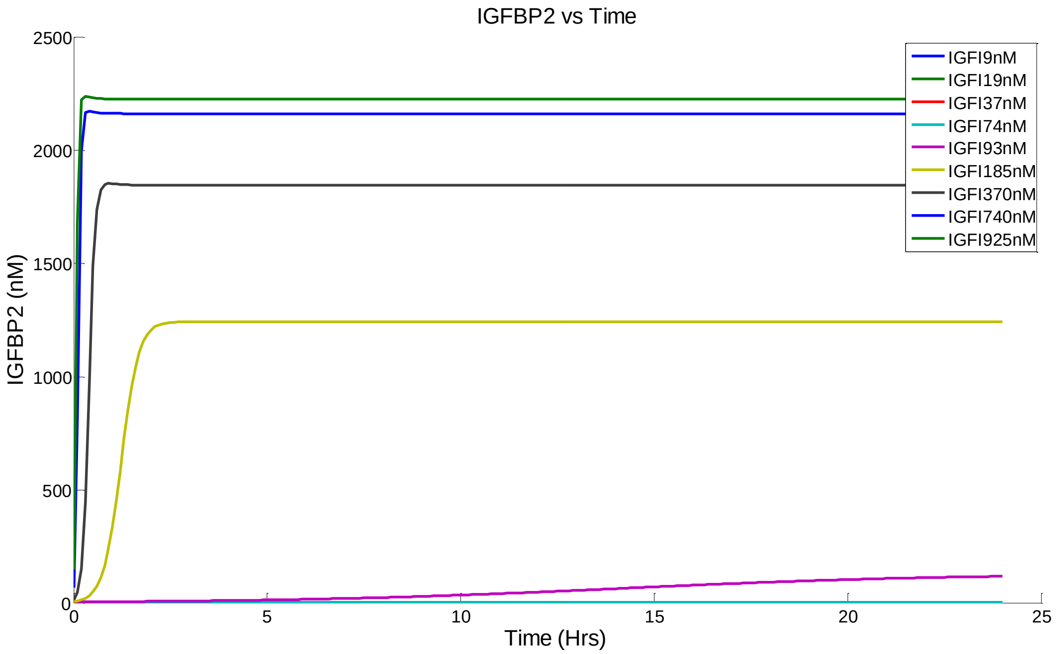

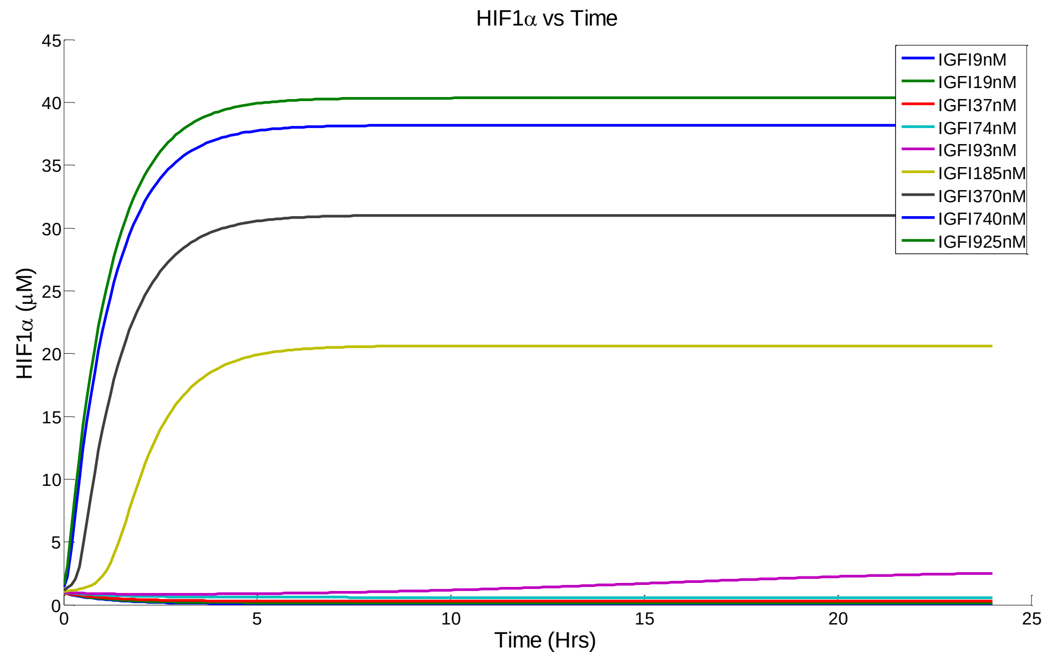

HIF1α vs Time
HIF1α (μM)
Time (Hrs)
IGFI9nM
IGFI19nM
IGFI37nM
IGFI74nM
IGFI93nM
IGFI185nM
IGFI370nM
IGFI740nM
IGFI925nM

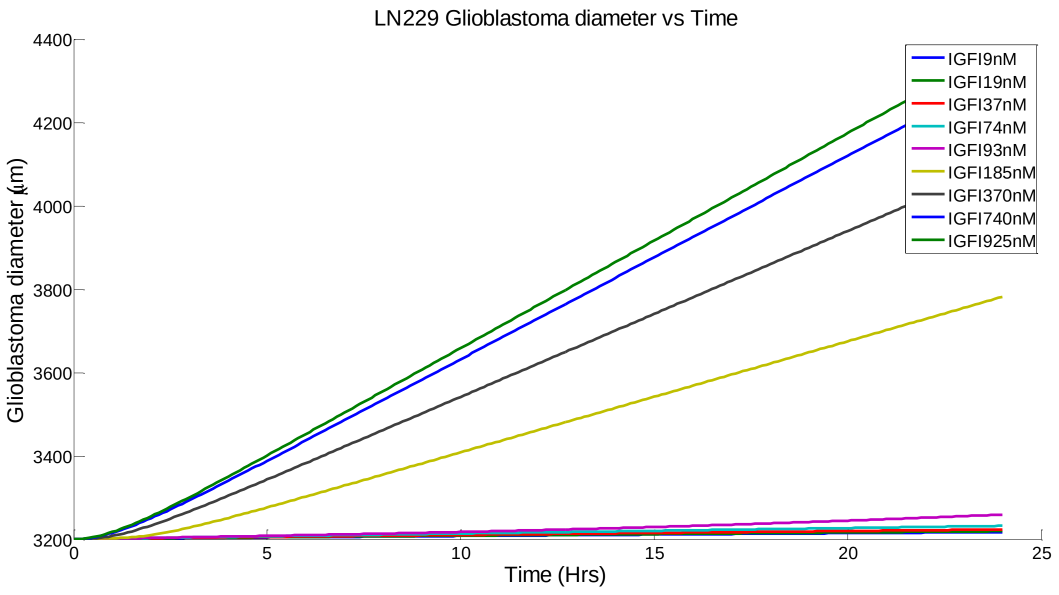

LN229 Glioblastoma diameter vs Time
Glioblastoma diameter (μm)
Time (Hrs)
IGFI9nM
IGFI19nM
IGFI37nM
IGFI74nM
IGFI93nM
IGFI185nM
IGFI370nM
IGFI740nM
IGFI925nM

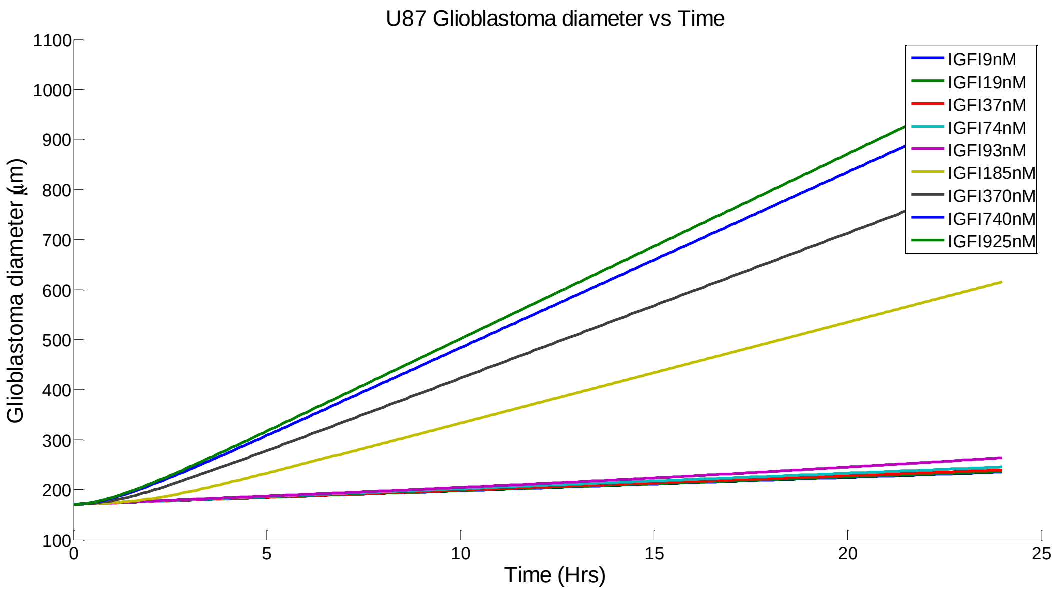
U87 Glioblastoma diameter vs Time
Glioblastoma diameter (μm)
Time (Hrs)
IGFI9nM
IGFI19nM
IGFI37nM
IGFI74nM
IGFI93nM
IGFI185nM
IGFI370nM
IGFI740nM
IGFI925nM

IGFBP2

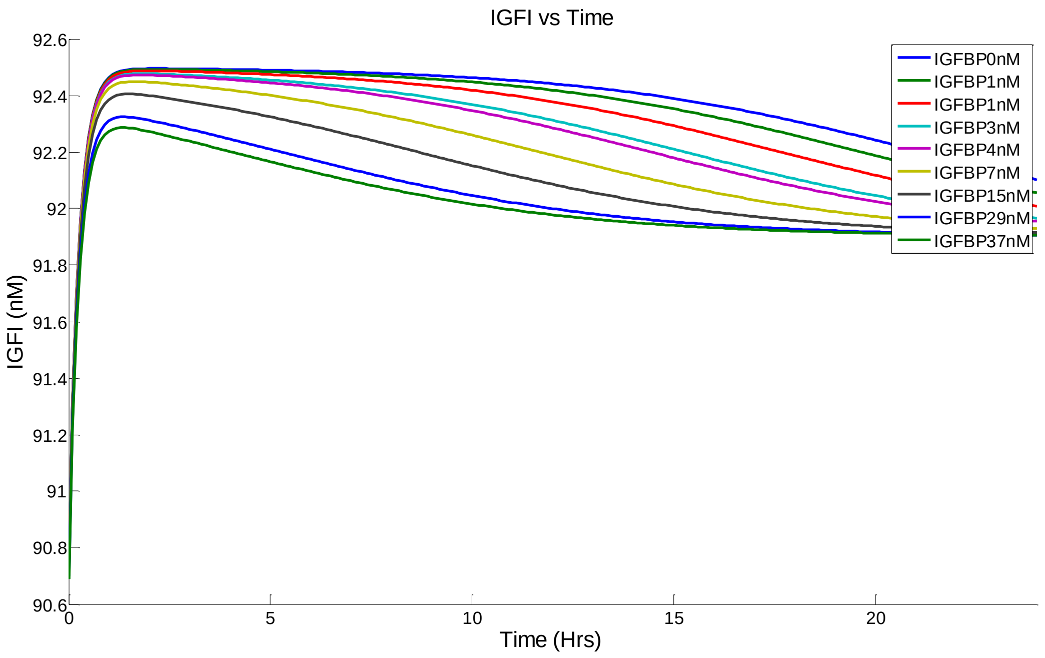
IGFI vs Time
IGFI (nM)
Time (Hrs)
IGFBP0nM
IGFBP1nM
IGFBP1nM
IGFBP3nM
IGFBP4nM
IGFBP7nM
IGFBP15nM
IGFBP29nM
IGFBP37nM

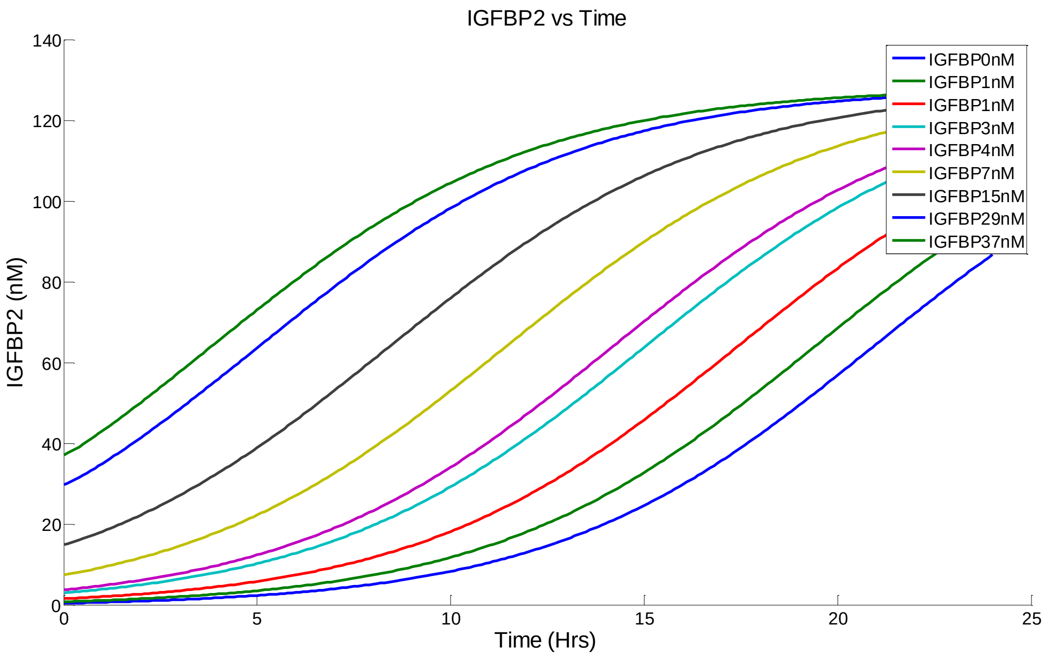
IGFBP2 vs Time
IGFBP2 (nM)
Time (Hrs)
IGFBP0nM
IGFBP1nM
IGFBP1nM
IGFBP3nM
IGFBP4nM
IGFBP7nM
IGFBP15nM
IGFBP29nM
IGFBP37nM

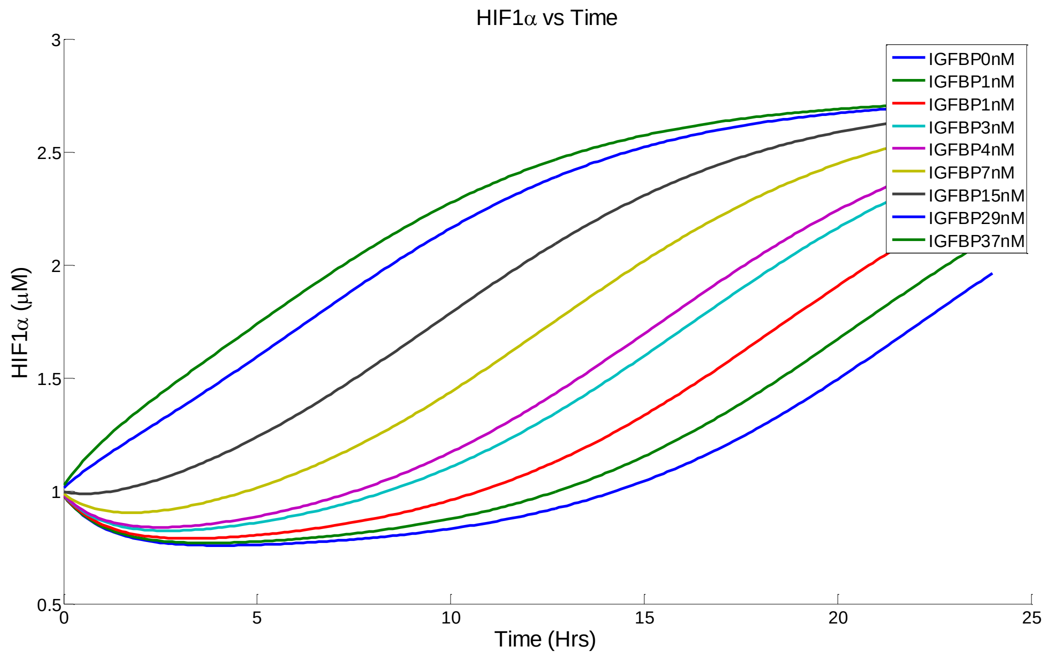
HIF1α vs Time
HIF1α (μM)
Time (Hrs)
IGFBP0nM
IGFBP1nM
IGFBP1nM
IGFBP3nM
IGFBP4nM
IGFBP7nM
IGFBP15nM
IGFBP29nM
IGFBP37nM

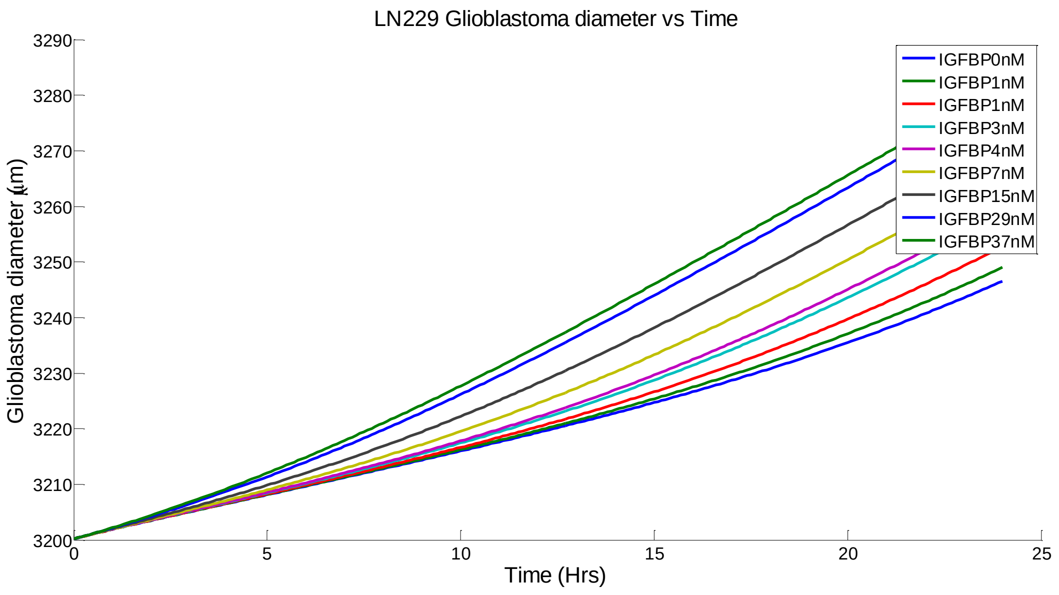
LN229 Glioblastoma diameter vs Time
Glioblastoma diameter (μm)
Time (Hrs)
3290
3280
3270
3260
3250
3240
3230
3220
3210
3200
0
5
10
15
20
25
IGFBP0nM
IGFBP1nM
IGFBP1nM
IGFBP3nM
IGFBP4nM
IGFBP7nM
IGFBP15nM
IGFBP29nM
IGFBP37nM

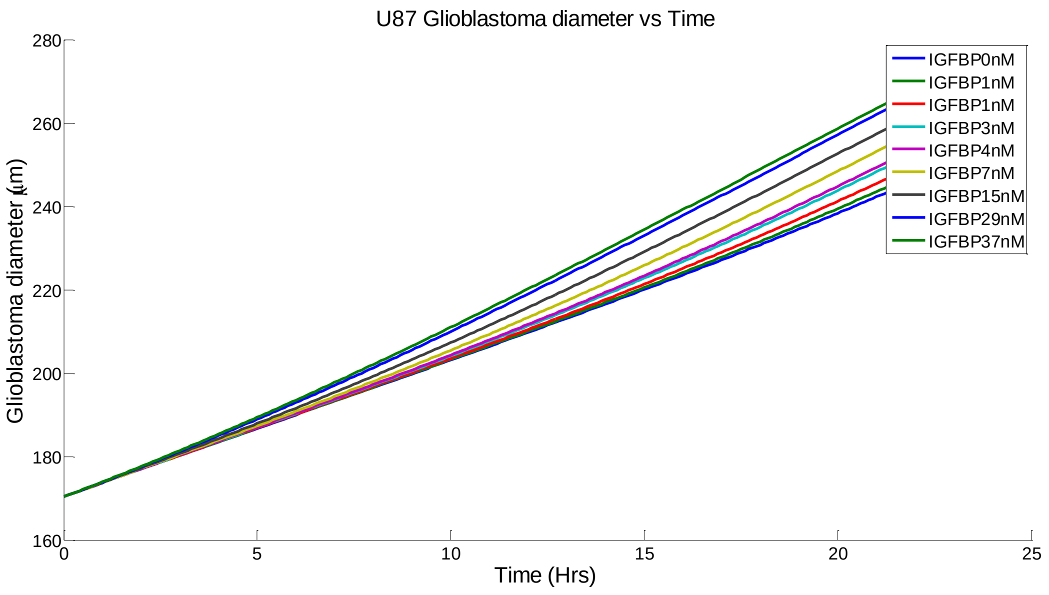
U87 Glioblastoma diameter vs Time
Glioblastoma diameter (μm)
Time (Hrs)
280
260
240
220
200
180
160
0
5
10
15
20
25
IGFBP0nM
IGFBP1nM
IGFBP1nM
IGFBP3nM
IGFBP4nM
IGFBP7nM
IGFBP15nM
IGFBP29nM
IGFBP37nM

IGFI-IGFBP2$_{complex}$

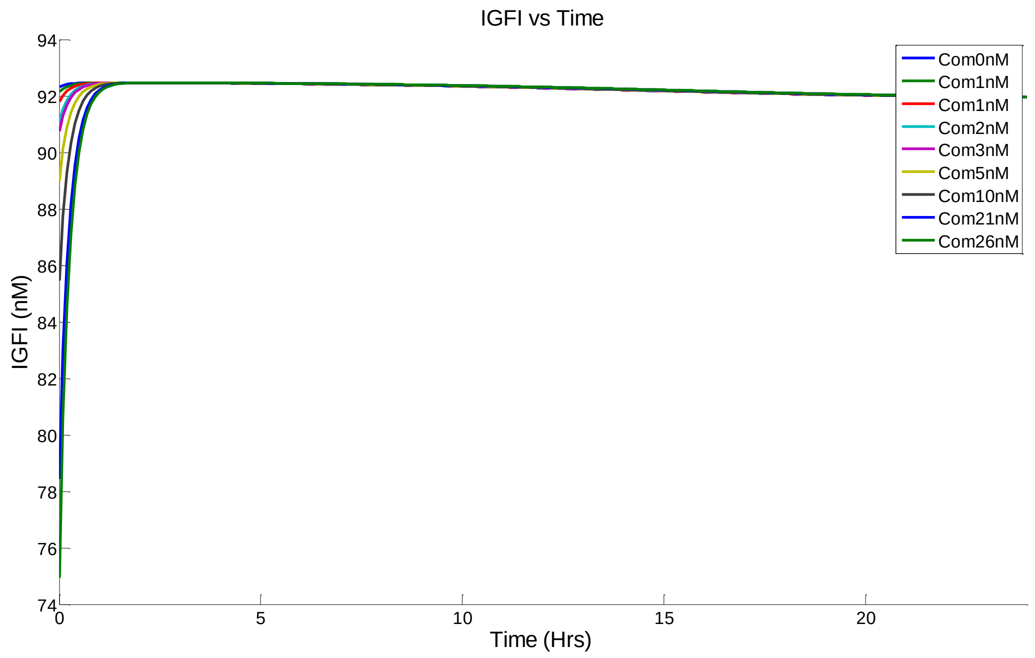

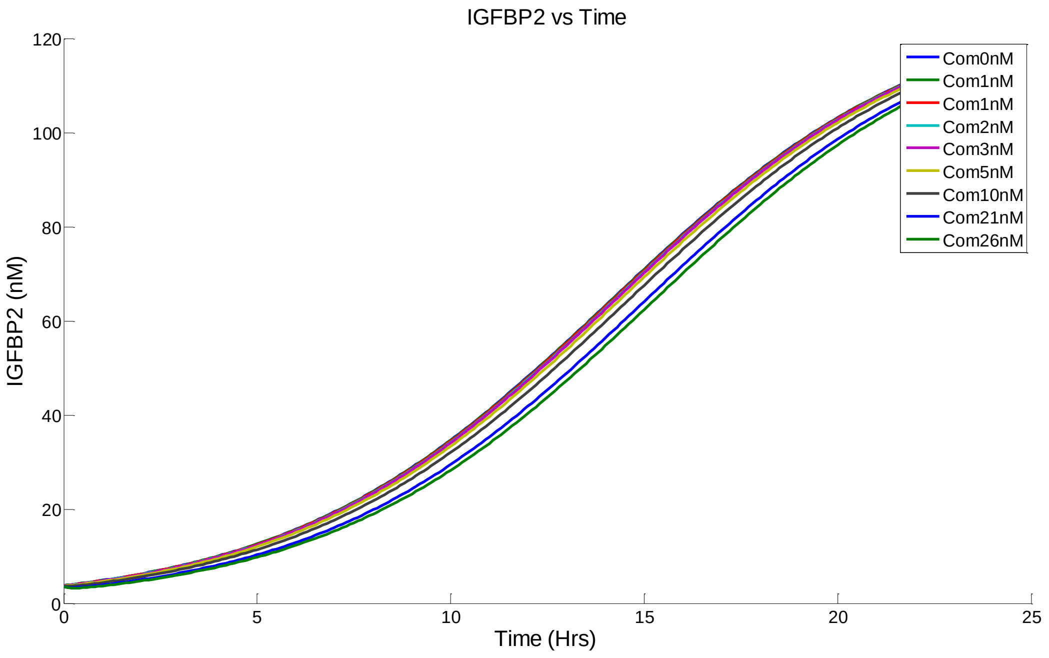

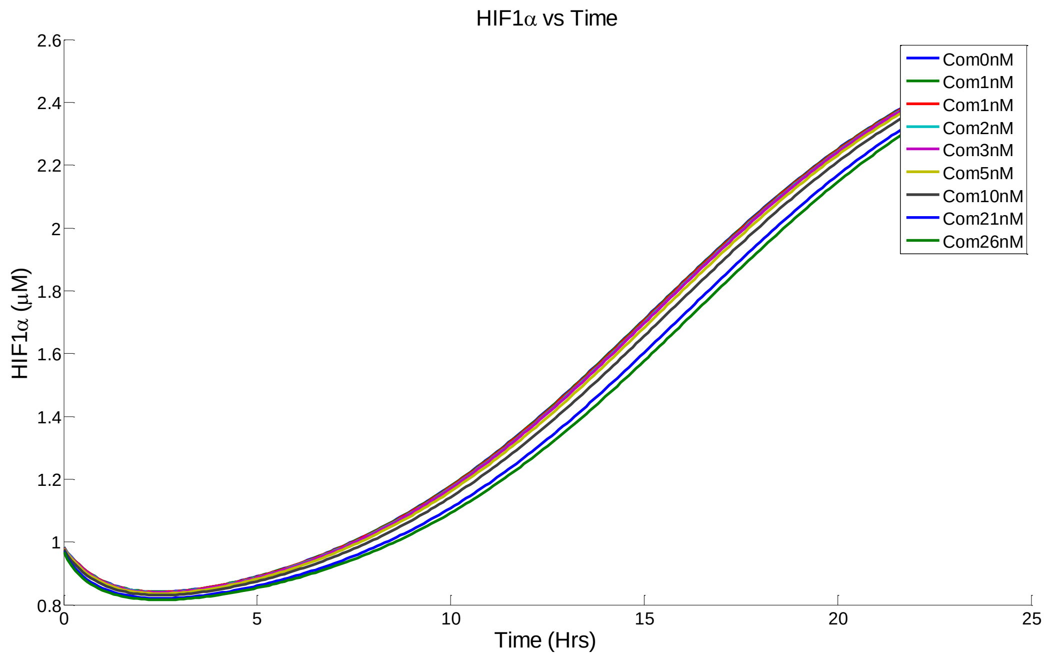
HIF1α vs Time
HIF1α (μM)
Time (Hrs)
2.6
2.4
2.2
2
1.8
1.6
1.4
1.2
1
0.8
0
5
10
15
20
25
Com0nM
Com1nM
Com1nM
Com2nM
Com3nM
Com5nM
Com10nM
Com21nM
Com26nM

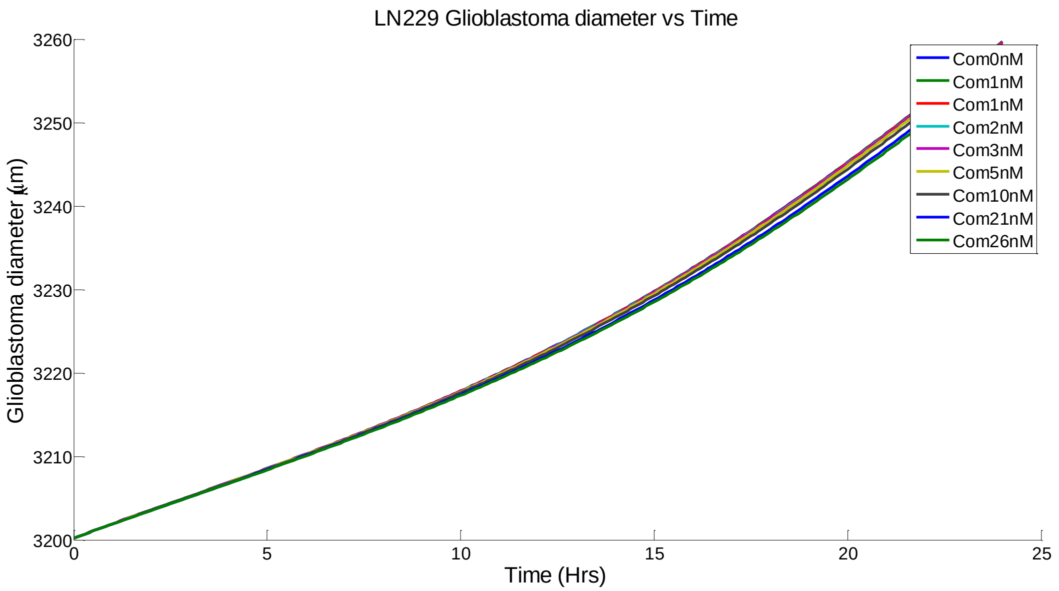
LN229 Glioblastoma diameter vs Time
Glioblastoma diameter (μm)
Time (Hrs)
3260
3250
3240
3230
3220
3210
3200
0
5
10
15
20
25
Com0nM
Com1nM
Com1nM
Com2nM
Com3nM
Com5nM
Com10nM
Com21nM
Com26nM

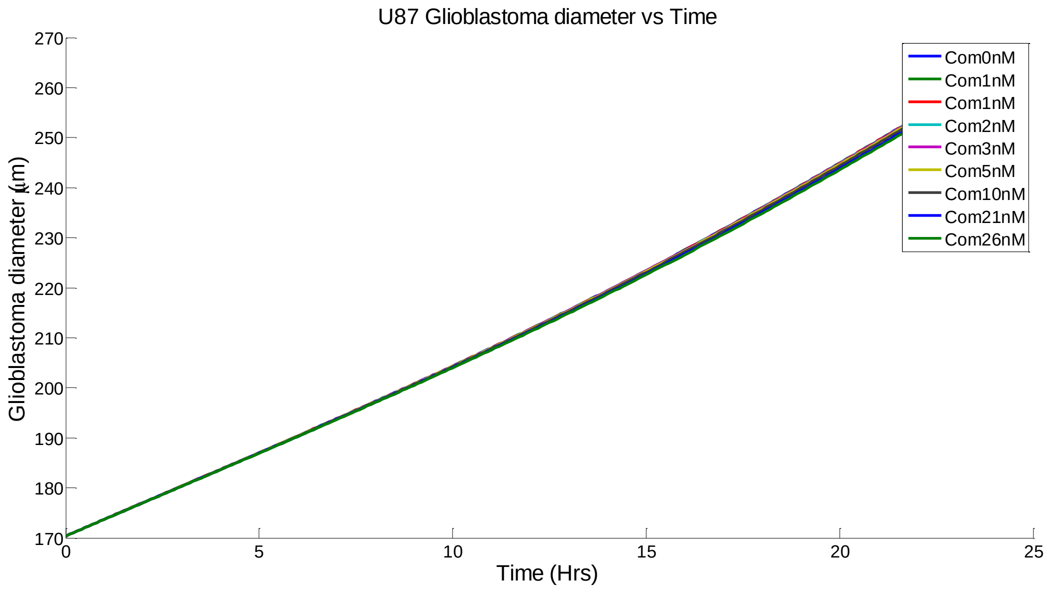
U87 Glioblastoma diameter vs Time
Glioblastoma diameter (μm)
Time (Hrs)
Com0nM
Com1nM
Com1nM
Com2nM
Com3nM
Com5nM
Com10nM
Com21nM
Com26nM

Oxygen

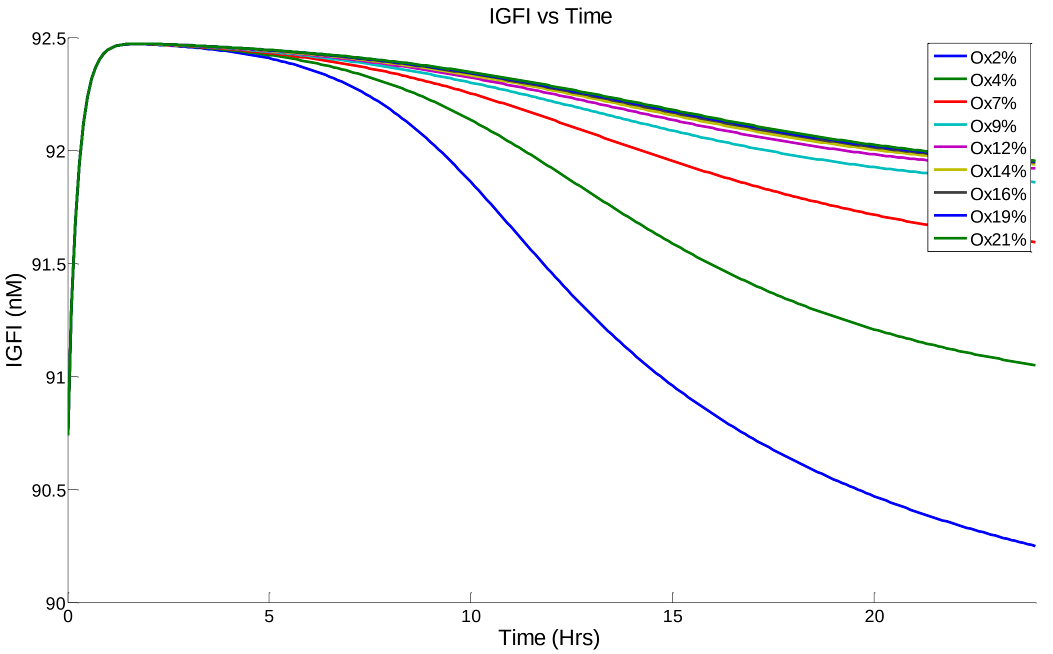
IGFI vs Time
IGFI (nM)
Time (Hrs)
Ox2%
Ox4%
Ox7%
Ox9%
Ox12%
Ox14%
Ox16%
Ox19%
Ox21%

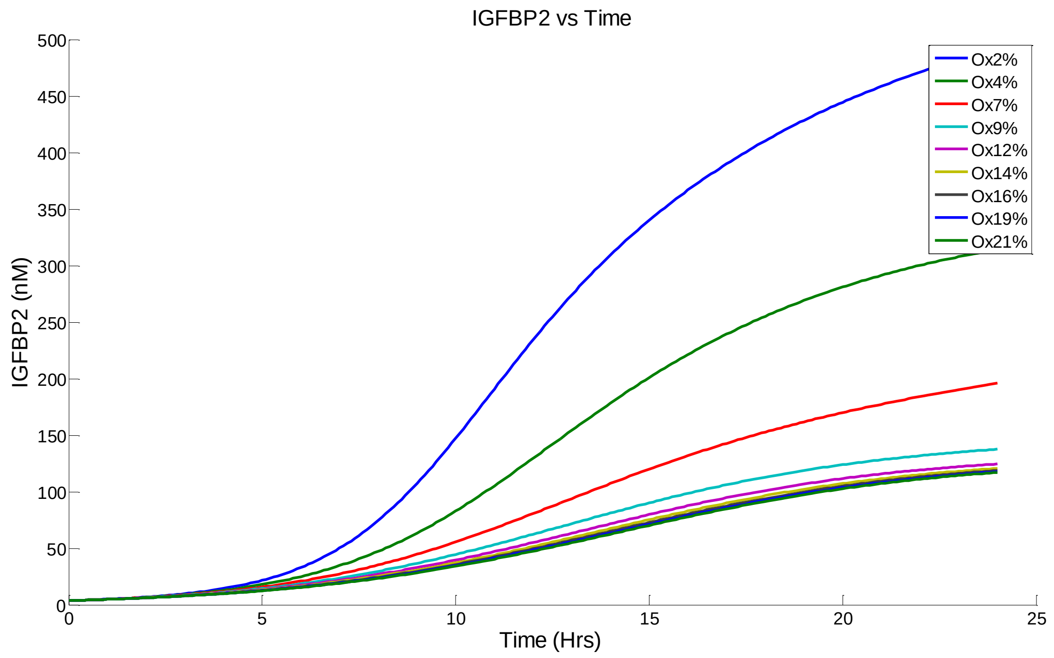
IGFBP2 vs Time
IGFBP2 (nM)
Time (Hrs)
Ox2%
Ox4%
Ox7%
Ox9%
Ox12%
Ox14%
Ox16%
Ox19%
Ox21%
0
50
100
150
200
250
300
350
400
450
500
0
5
10
15
20
25

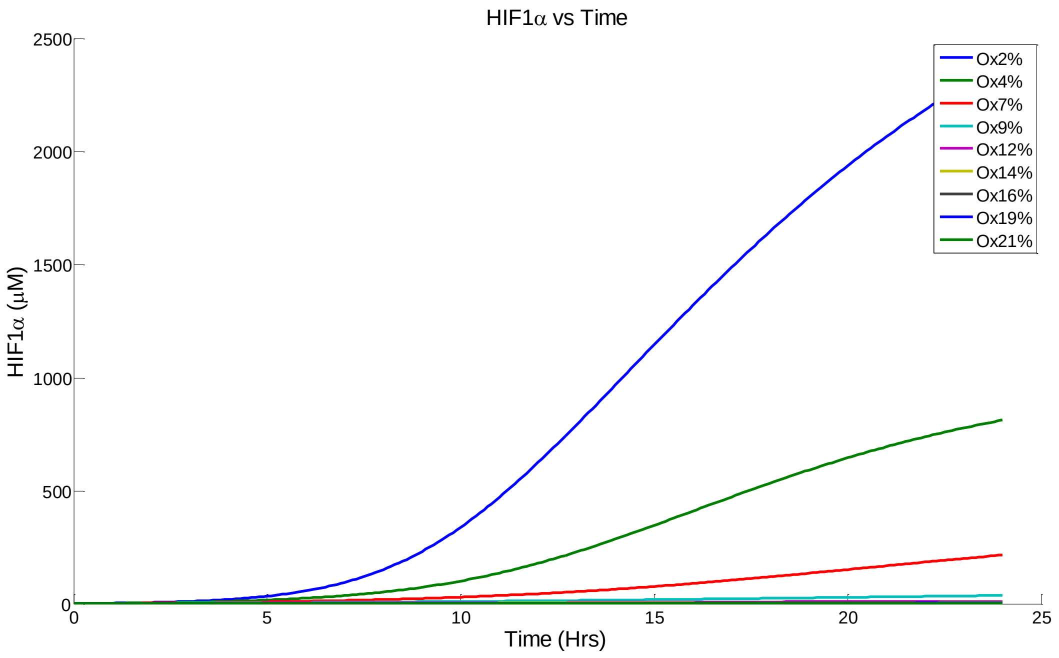
HIF1α vs Time
HIF1α (μM)
Time (Hrs)
Ox2%
Ox4%
Ox7%
Ox9%
Ox12%
Ox14%
Ox16%
Ox19%
Ox21%
0
500
1000
1500
2000
2500
0
5
10
15
20
25

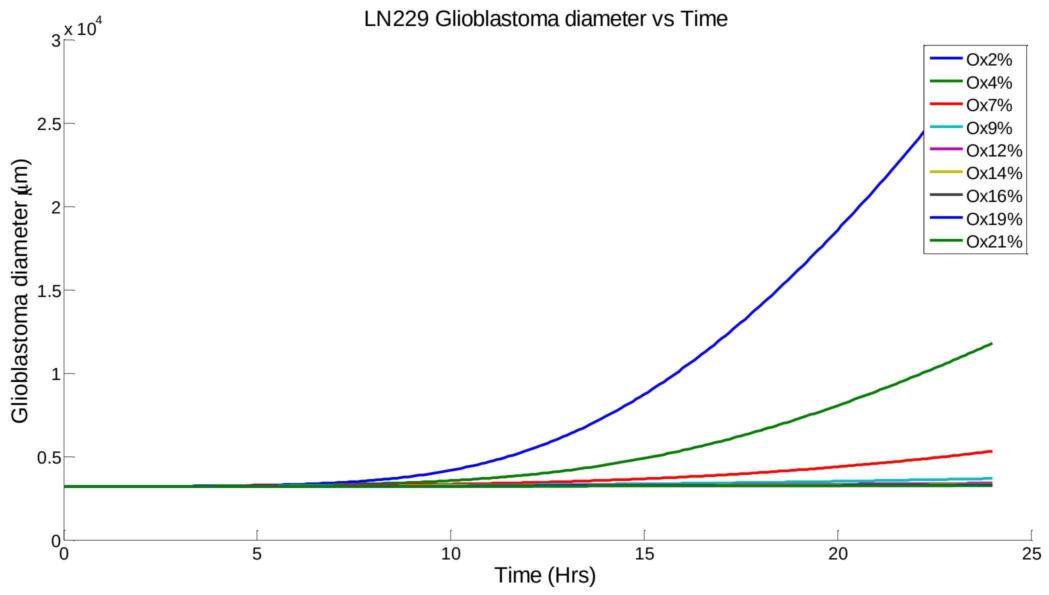
LN229 Glioblastoma diameter vs Time
x 10^4
Glioblastoma diameter (μm)
Time (Hrs)
0
0.5
1
1.5
2
2.5
3
0
5
10
15
20
25
Ox2%
Ox4%
Ox7%
Ox9%
Ox12%
Ox14%
Ox16%
Ox19%
Ox21%

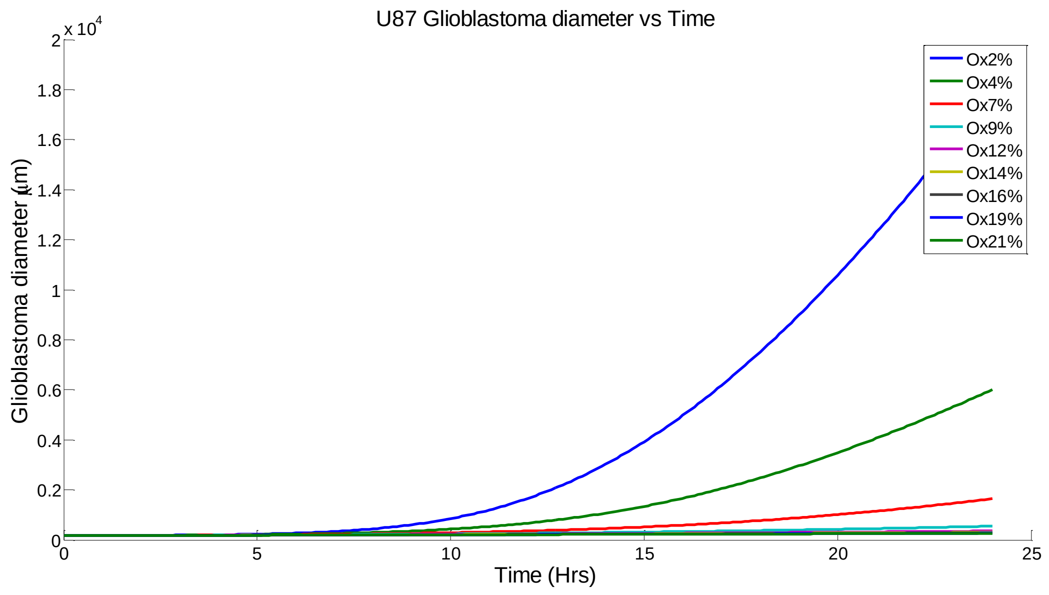
U87 Glioblastoma diameter vs Time
x 10^4
Glioblastoma diameter (μm)
Time (Hrs)
0
0.2
0.4
0.6
0.8
1
1.2
1.4
1.6
1.8
2
0
5
10
15
20
25
Ox2%
Ox4%
Ox7%
Ox9%
Ox12%
Ox14%
Ox16%
Ox19%
Ox21%

HIF1α

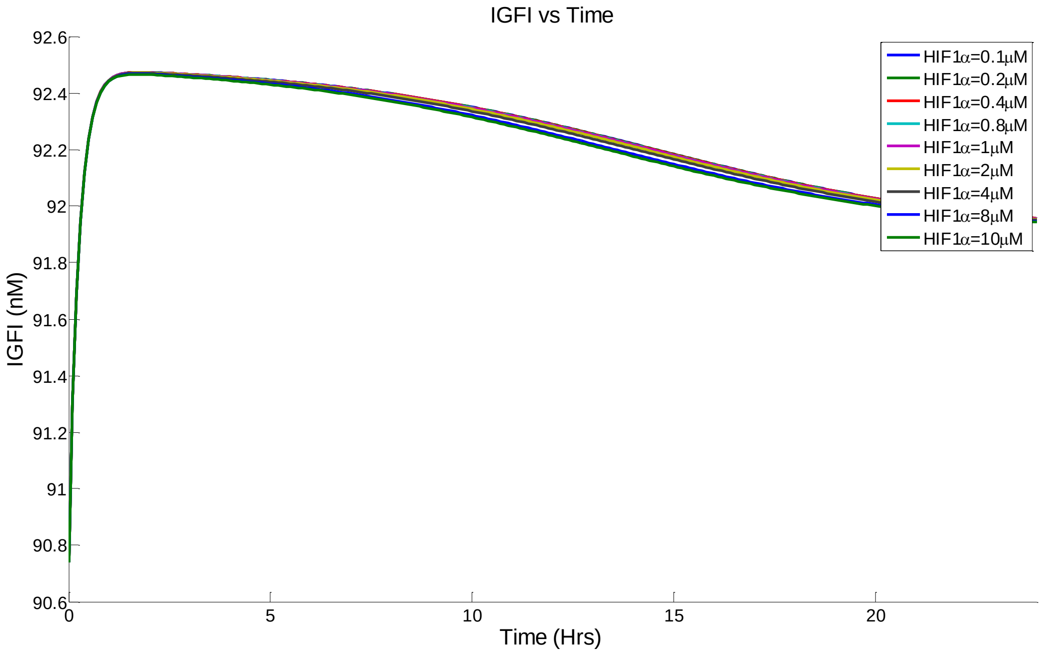
IGFI vs Time
92.6
92.4
92.2
92
91.8
91.6
91.4
91.2
91
90.8
90.6
0
5
10
15
20
IGFI (nM)
Time (Hrs)
HIF1α=0.1μM
HIF1α=0.2μM
HIF1α=0.4μM
HIF1α=0.8μM
HIF1α=1μM
HIF1α=2μM
HIF1α=4μM
HIF1α=8μM
HIF1α=10μM

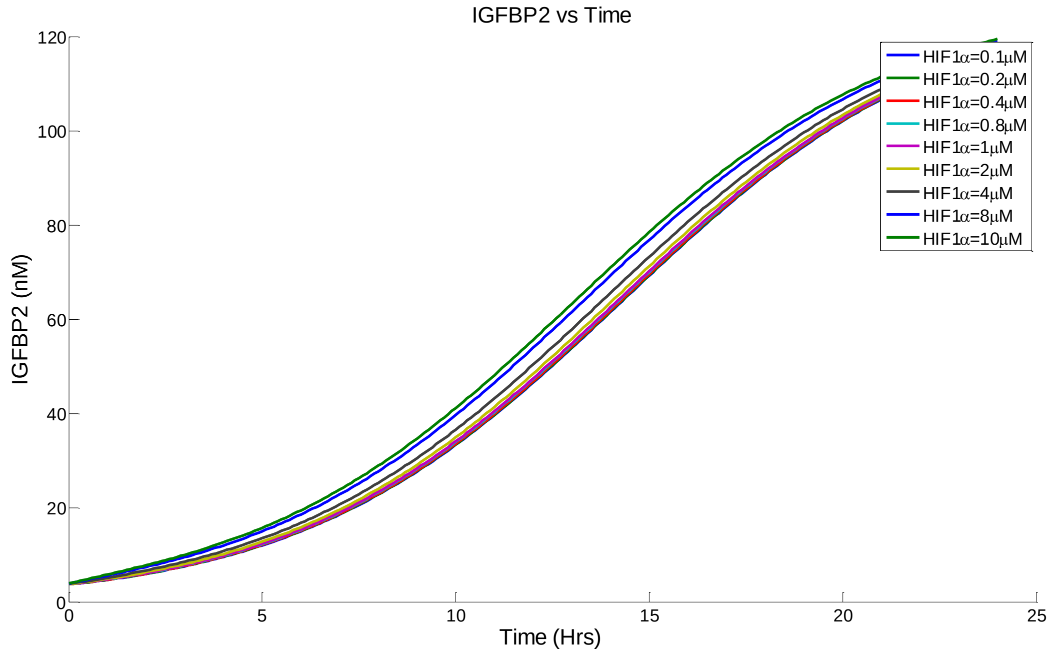
IGFBP2 vs Time
120
100
80
60
40
20
0
0
5
10
15
20
25
IGFBP2 (nM)
Time (Hrs)
HIF1α=0.1μM
HIF1α=0.2μM
HIF1α=0.4μM
HIF1α=0.8μM
HIF1α=1μM
HIF1α=2μM
HIF1α=4μM
HIF1α=8μM
HIF1α=10μM

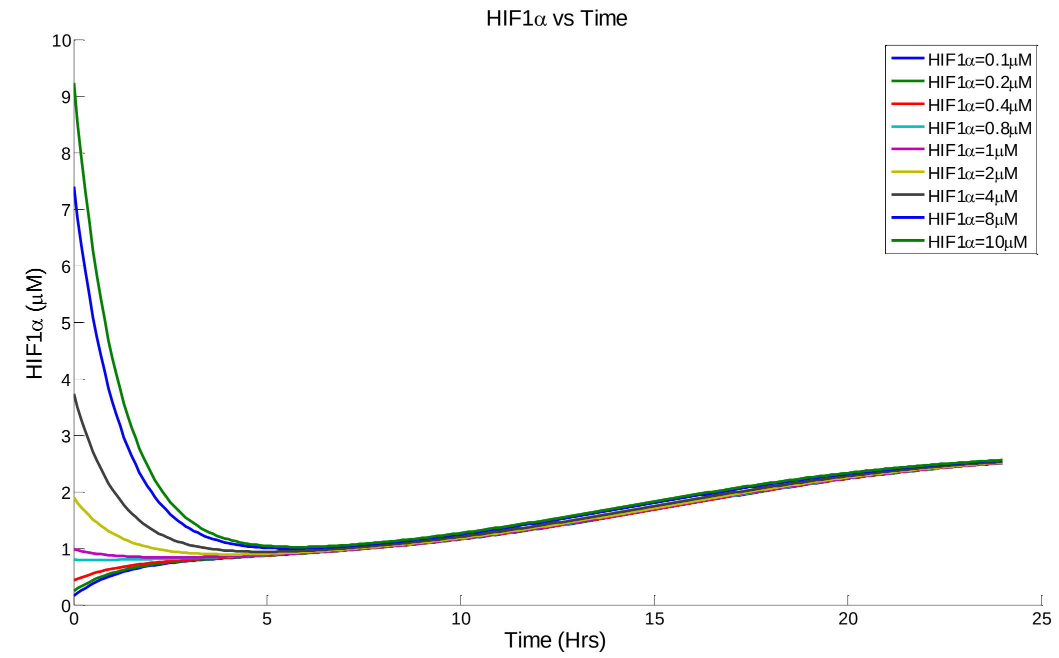

HIF1α vs Time
HIF1α (μM)
Time (Hrs)
HIF1α=0.1μM
HIF1α=0.2μM
HIF1α=0.4μM
HIF1α=0.8μM
HIF1α=1μM
HIF1α=2μM
HIF1α=4μM
HIF1α=8μM
HIF1α=10μM

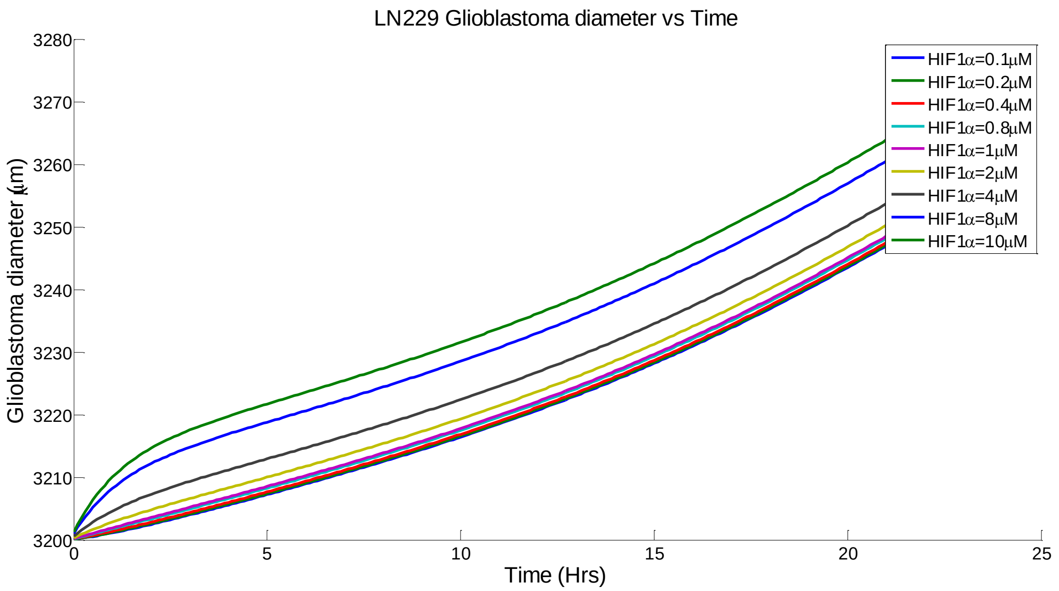

LN229 Glioblastoma diameter vs Time
Glioblastoma diameter (μm)
Time (Hrs)
HIF1α=0.1μM
HIF1α=0.2μM
HIF1α=0.4μM
HIF1α=0.8μM
HIF1α=1μM
HIF1α=2μM
HIF1α=4μM
HIF1α=8μM
HIF1α=10μM

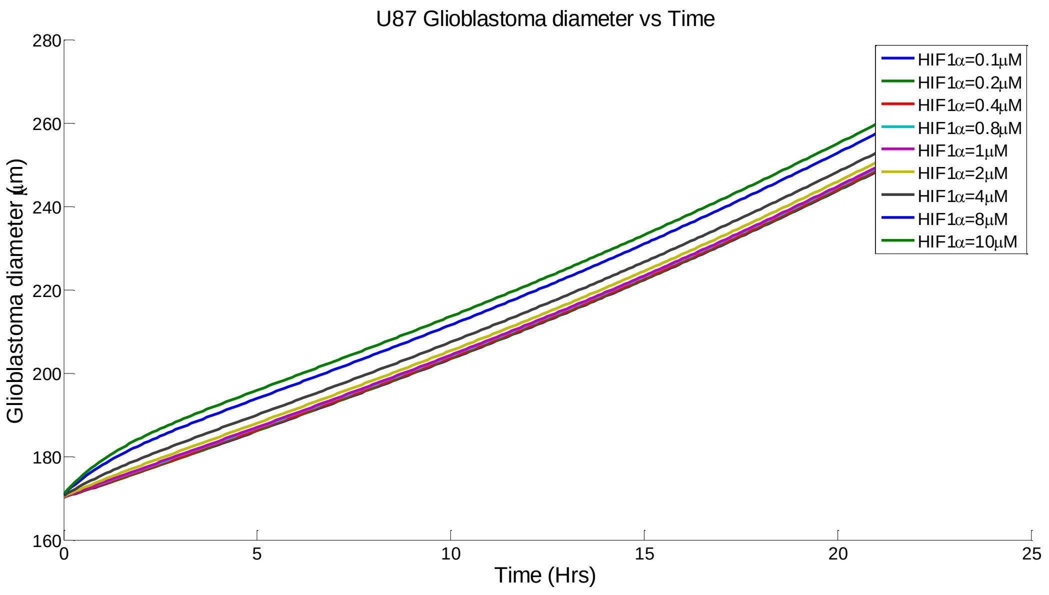

Senstivity analysis: rate constants

k1

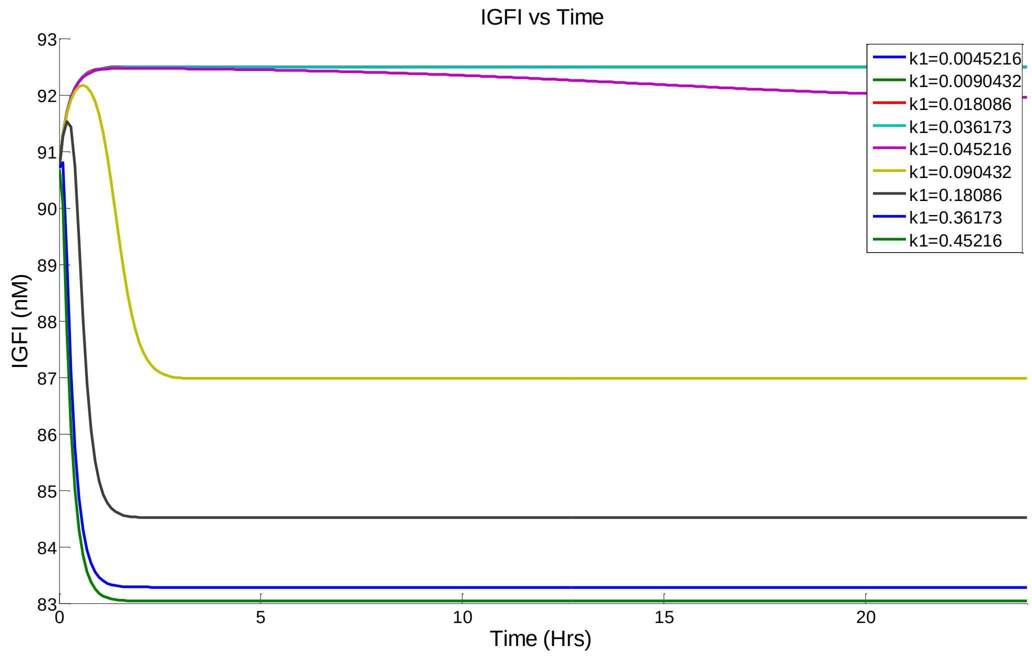

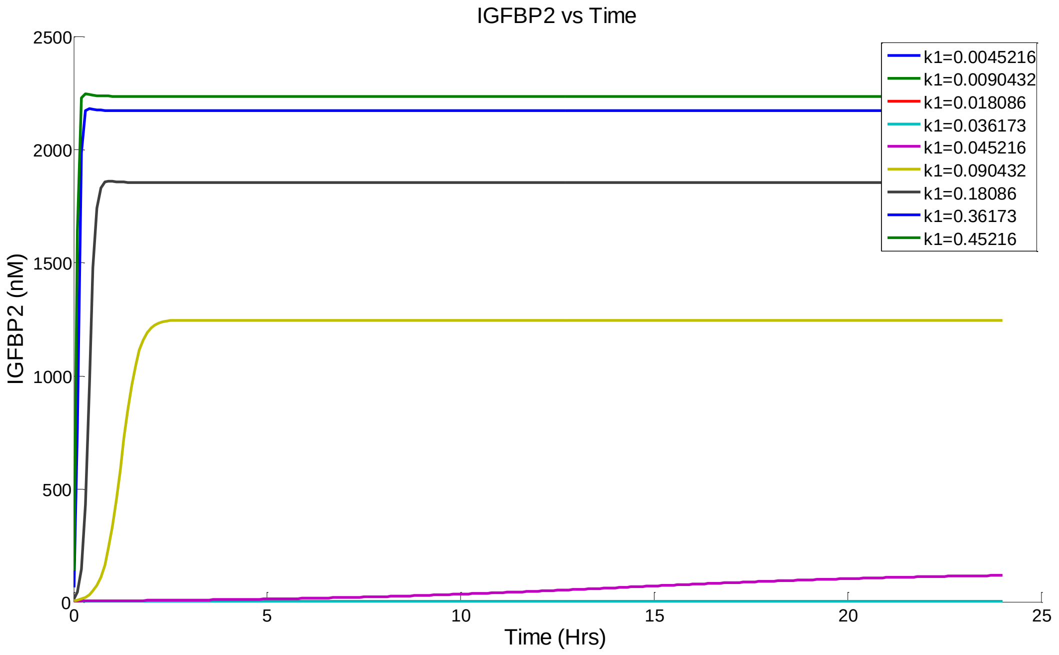
IGFBP2 vs Time
IGFBP2 (nM)
Time (Hrs)
k1=0.0045216
k1=0.0090432
k1=0.018086
k1=0.036173
k1=0.045216
k1=0.090432
k1=0.18086
k1=0.36173
k1=0.45216

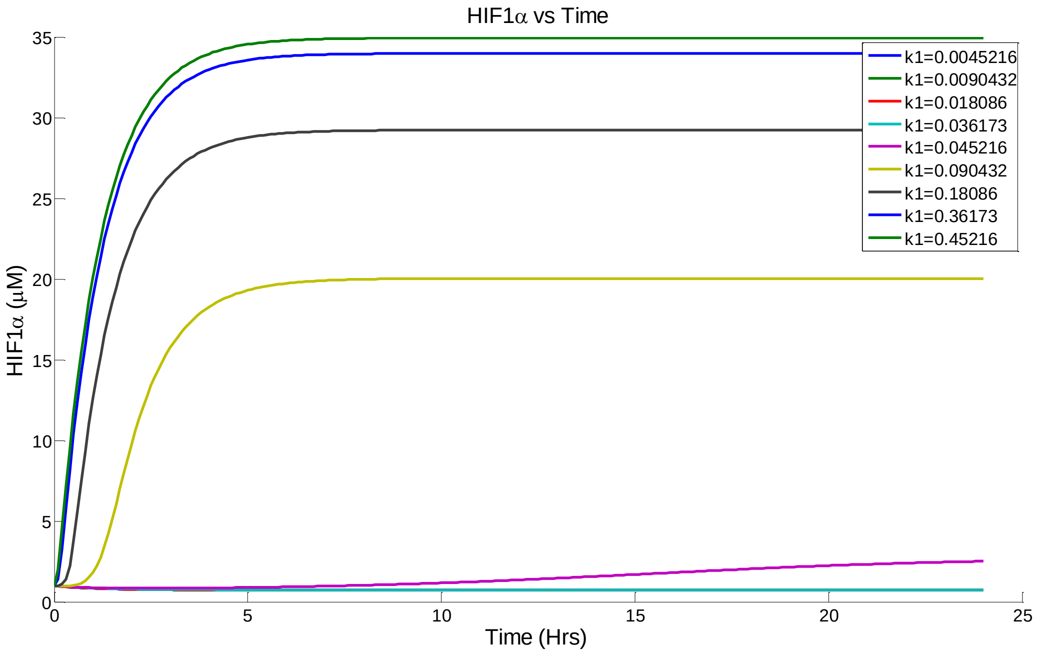
HIF1α vs Time
HIF1α (μM)
Time (Hrs)
k1=0.0045216
k1=0.0090432
k1=0.018086
k1=0.036173
k1=0.045216
k1=0.090432
k1=0.18086
k1=0.36173
k1=0.45216

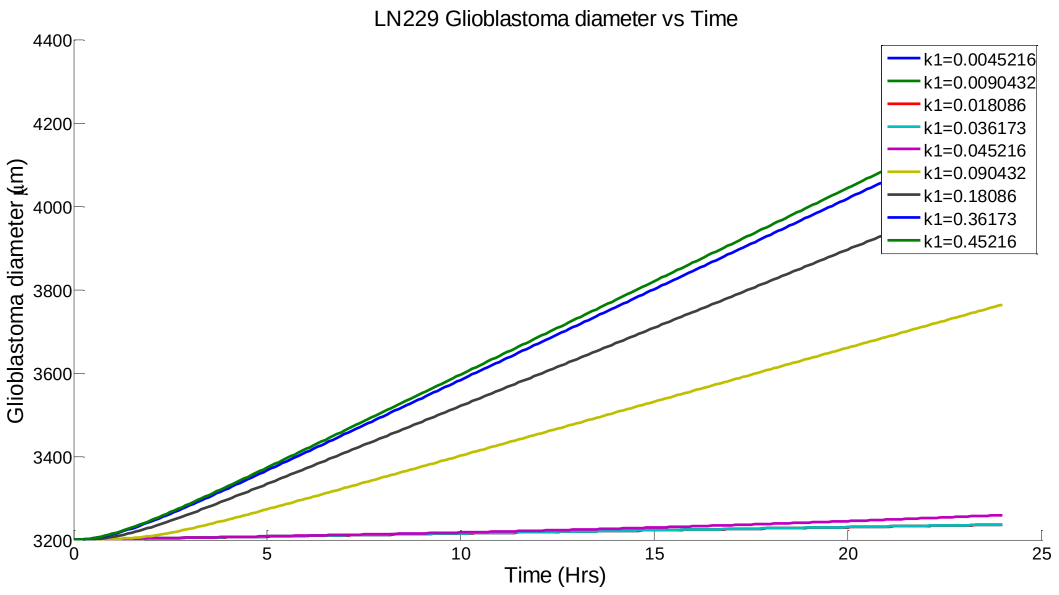

LN229 Glioblastoma diameter vs Time
Glioblastoma diameter (μm)
Time (Hrs)
4400
4200
4000
3800
3600
3400
3200
0
5
10
15
20
25
k1=0.0045216
k1=0.0090432
k1=0.018086
k1=0.036173
k1=0.045216
k1=0.090432
k1=0.18086
k1=0.36173
k1=0.45216

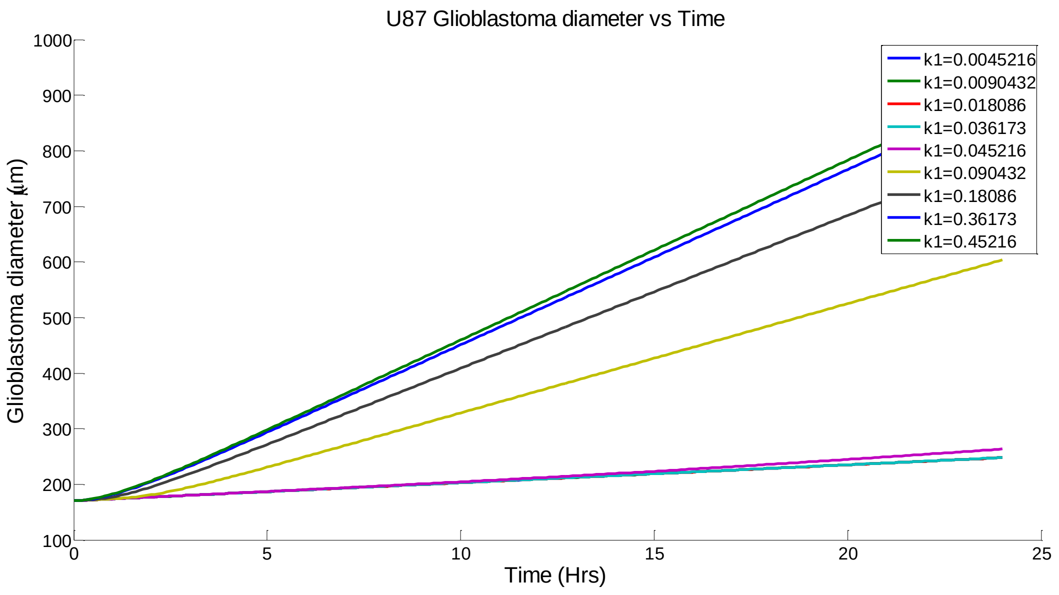

U87 Glioblastoma diameter vs Time
Glioblastoma diameter (μm)
Time (Hrs)
1000
900
800
700
600
500
400
300
200
100
0
5
10
15
20
25
k1=0.0045216
k1=0.0090432
k1=0.018086
k1=0.036173
k1=0.045216
k1=0.090432
k1=0.18086
k1=0.36173
k1=0.45216

k2

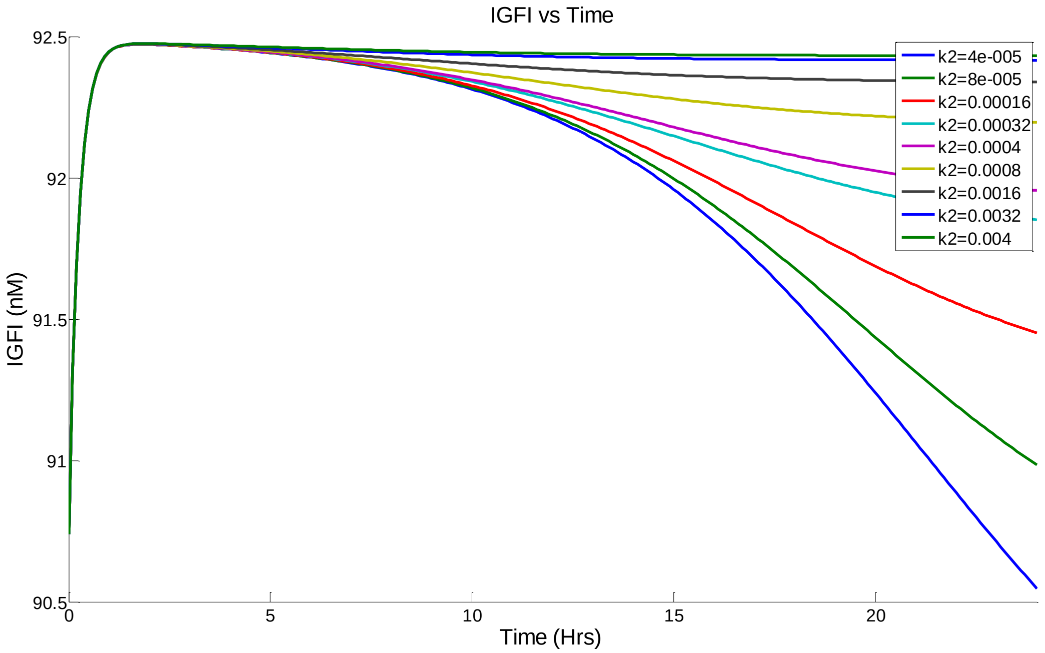
IGFI vs Time
IGFI (nM)
92.5
92
91.5
91
90.5
0
5
10
15
20
Time (Hrs)
k2=4e-005
k2=8e-005
k2=0.00016
k2=0.00032
k2=0.0004
k2=0.0008
k2=0.0016
k2=0.0032
k2=0.004

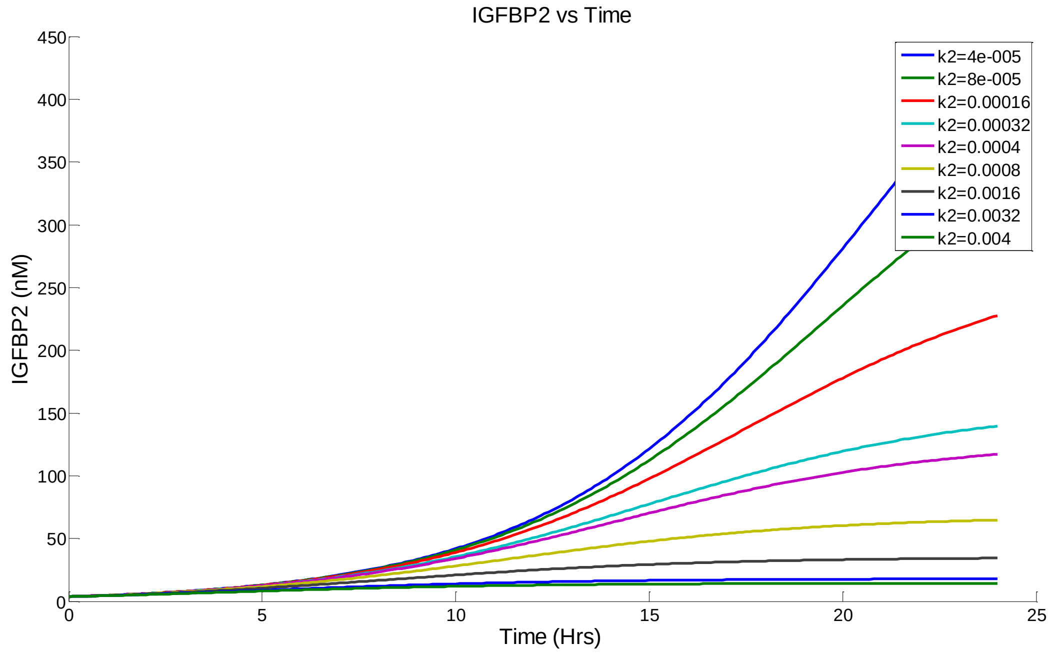
IGFBP2 vs Time
IGFBP2 (nM)
450
400
350
300
250
200
150
100
50
0
0
5
10
15
20
25
Time (Hrs)
k2=4e-005
k2=8e-005
k2=0.00016
k2=0.00032
k2=0.0004
k2=0.0008
k2=0.0016
k2=0.0032
k2=0.004

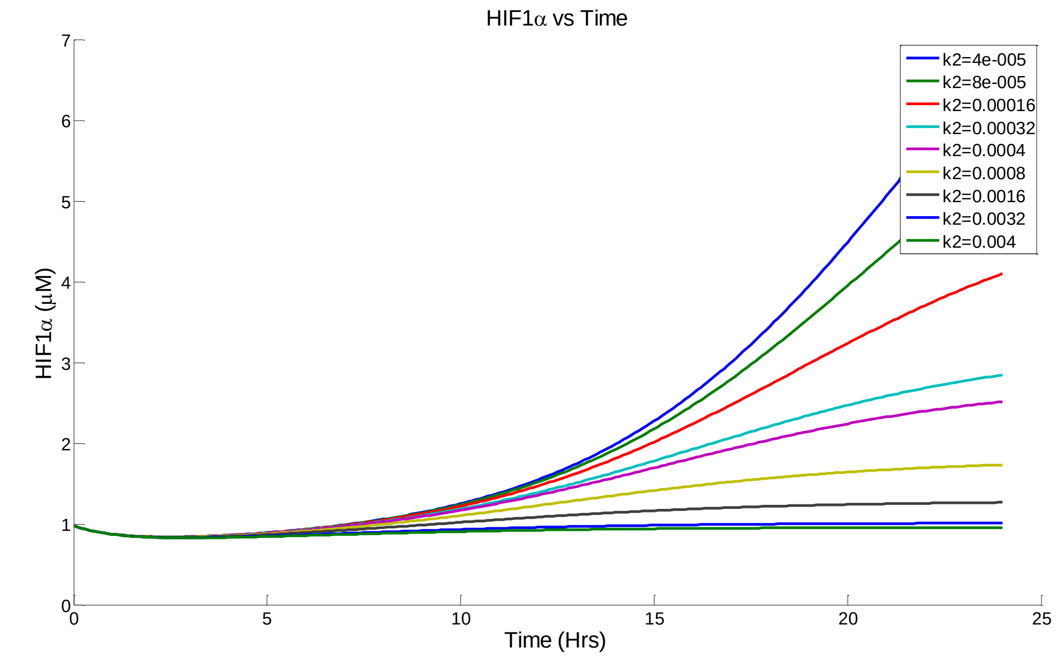
HIF1α vs Time
HIF1α (μM)
Time (Hrs)
0
1
2
3
4
5
6
7
0
5
10
15
20
25
k2=4e-005
k2=8e-005
k2=0.00016
k2=0.00032
k2=0.0004
k2=0.0008
k2=0.0016
k2=0.0032
k2=0.004

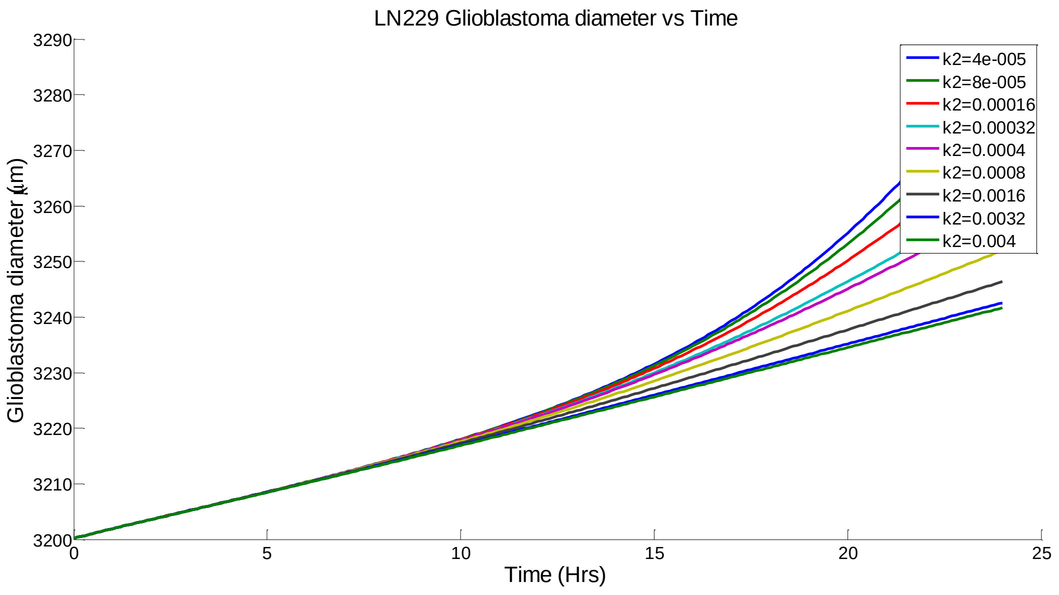
LN229 Glioblastoma diameter vs Time
Glioblastoma diameter (μm)
Time (Hrs)
3200
3210
3220
3230
3240
3250
3260
3270
3280
3290
0
5
10
15
20
25
k2=4e-005
k2=8e-005
k2=0.00016
k2=0.00032
k2=0.0004
k2=0.0008
k2=0.0016
k2=0.0032
k2=0.004

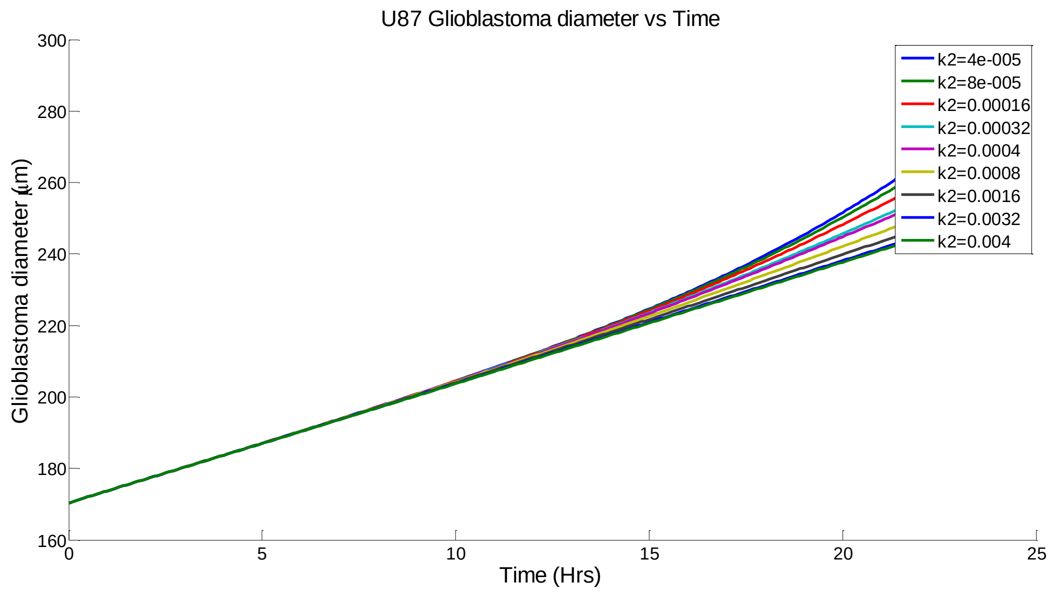
U87 Glioblastoma diameter vs Time
Glioblastoma diameter (μm)
Time (Hrs)
k2=4e-005
k2=8e-005
k2=0.00016
k2=0.00032
k2=0.0004
k2=0.0008
k2=0.0016
k2=0.0032
k2=0.004

k3

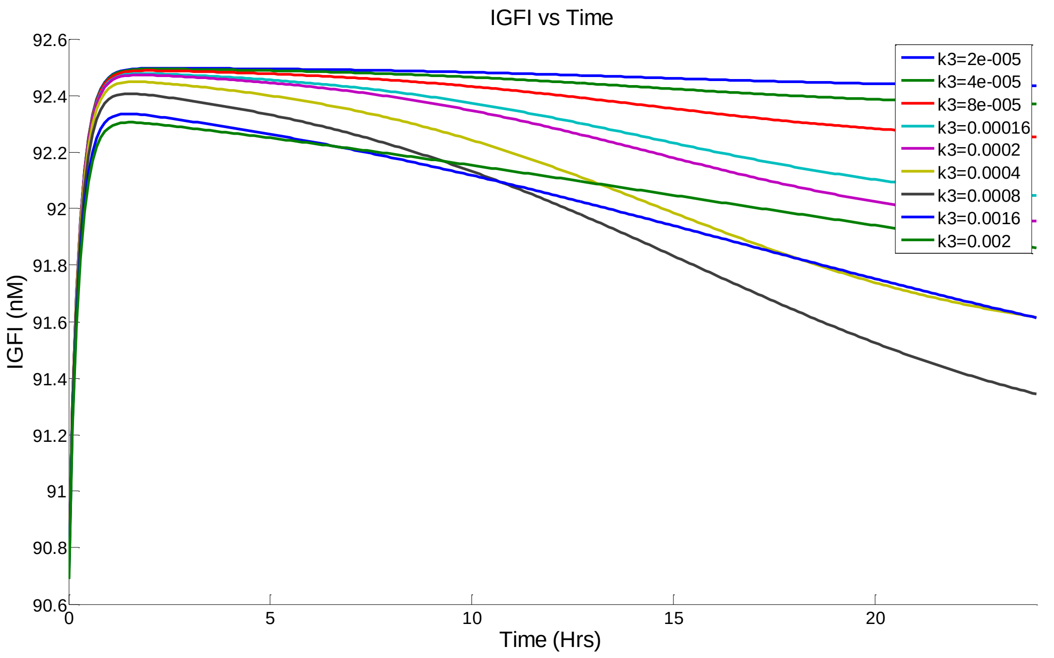
IGFI vs Time
IGFI (nM)
Time (Hrs)
k3=2e-005
k3=4e-005
k3=8e-005
k3=0.00016
k3=0.0002
k3=0.0004
k3=0.0008
k3=0.0016
k3=0.002

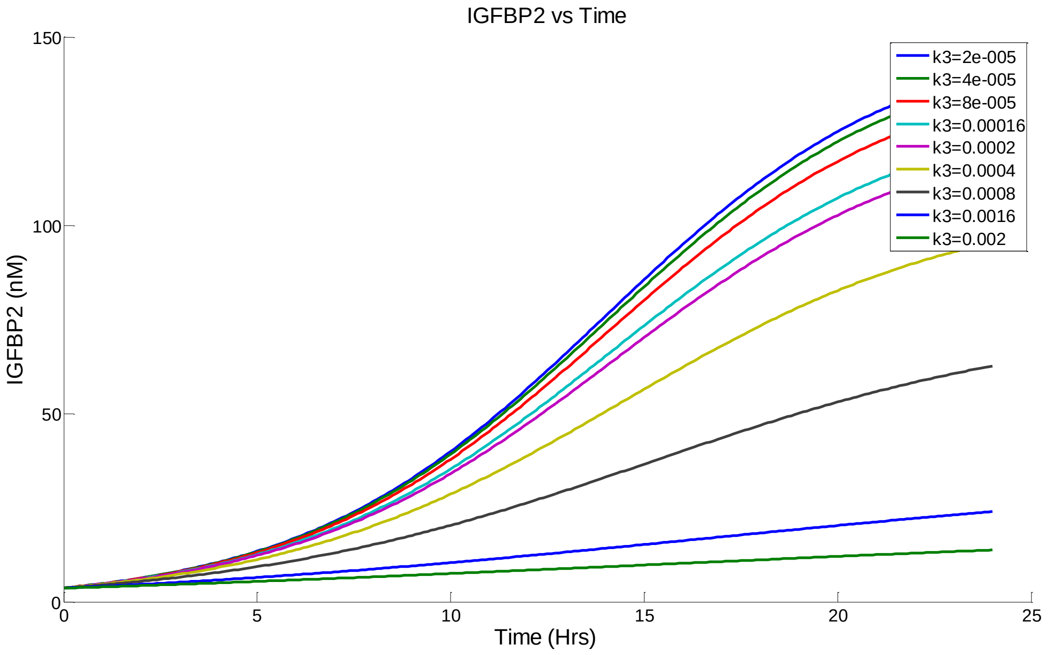
IGFBP2 vs Time
IGFBP2 (nM)
Time (Hrs)
0
50
100
150
0
5
10
15
20
25
k3=2e-005
k3=4e-005
k3=8e-005
k3=0.00016
k3=0.0002
k3=0.0004
k3=0.0008
k3=0.0016
k3=0.002

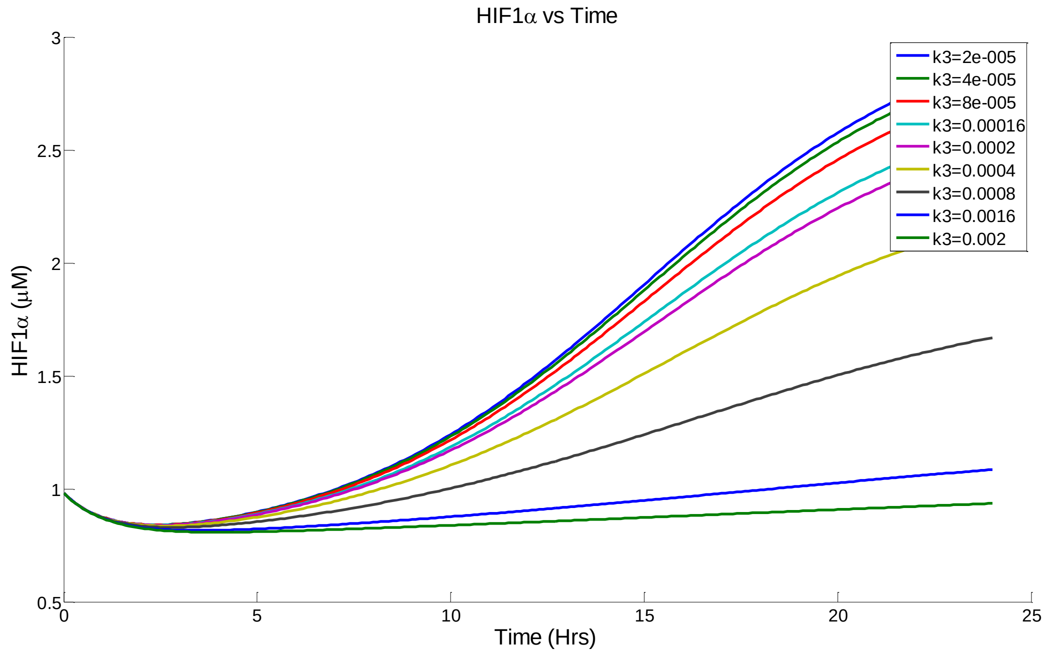
HIF1α vs Time
HIF1α (μM)
Time (Hrs)
0.5
1
1.5
2
2.5
3
0
5
10
15
20
25
k3=2e-005
k3=4e-005
k3=8e-005
k3=0.00016
k3=0.0002
k3=0.0004
k3=0.0008
k3=0.0016
k3=0.002

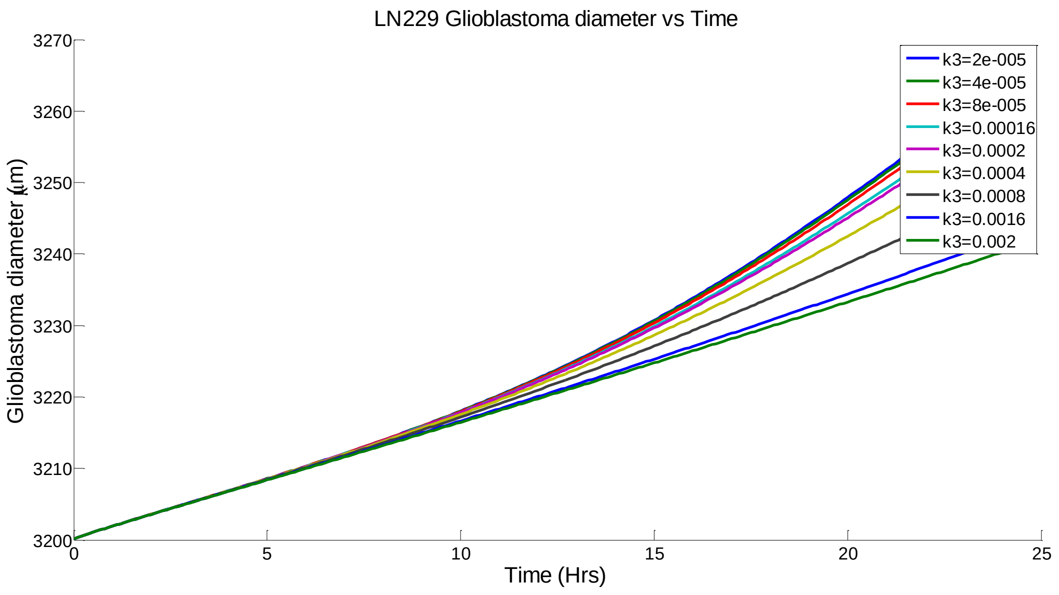
LN229 Glioblastoma diameter vs Time
Glioblastoma diameter (μm)
Time (Hrs)
3270
3260
3250
3240
3230
3220
3210
3200
0
5
10
15
20
25
k3=2e-005
k3=4e-005
k3=8e-005
k3=0.00016
k3=0.0002
k3=0.0004
k3=0.0008
k3=0.0016
k3=0.002

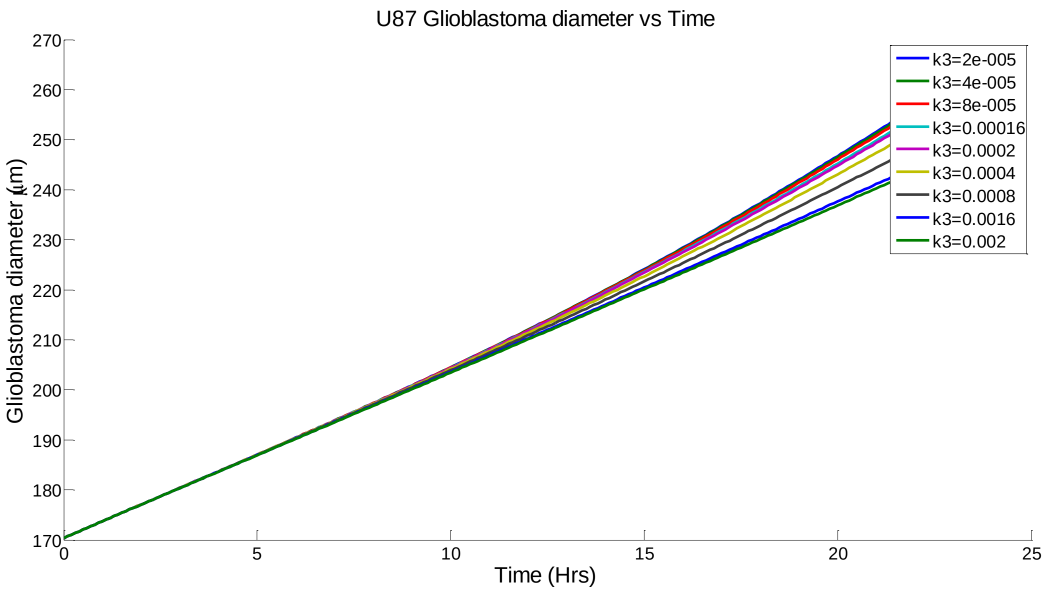
U87 Glioblastoma diameter vs Time
Glioblastoma diameter (μm)
Time (Hrs)
270
260
250
240
230
220
210
200
190
180
170
0
5
10
15
20
25
k3=2e-005
k3=4e-005
k3=8e-005
k3=0.00016
k3=0.0002
k3=0.0004
k3=0.0008
k3=0.0016
k3=0.002

k4

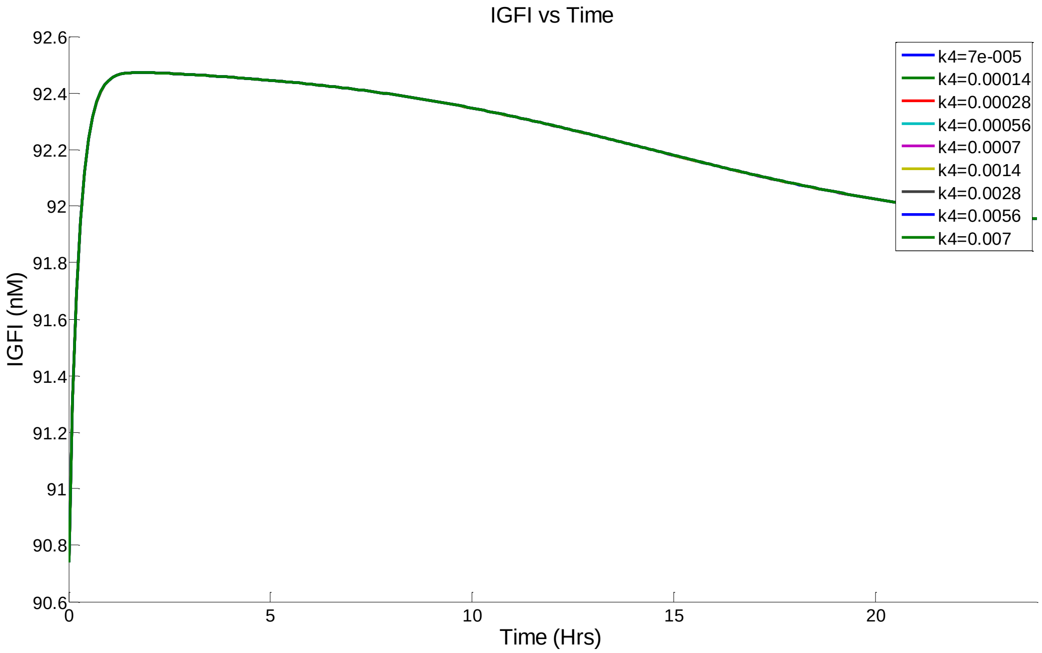
IGFI vs Time
IGFI (nM)
Time (Hrs)
92.6
92.4
92.2
92
91.8
91.6
91.4
91.2
91
90.8
90.6
0
5
10
15
20
k4=7e-005
k4=0.00014
k4=0.00028
k4=0.00056
k4=0.0007
k4=0.0014
k4=0.0028
k4=0.0056
k4=0.007

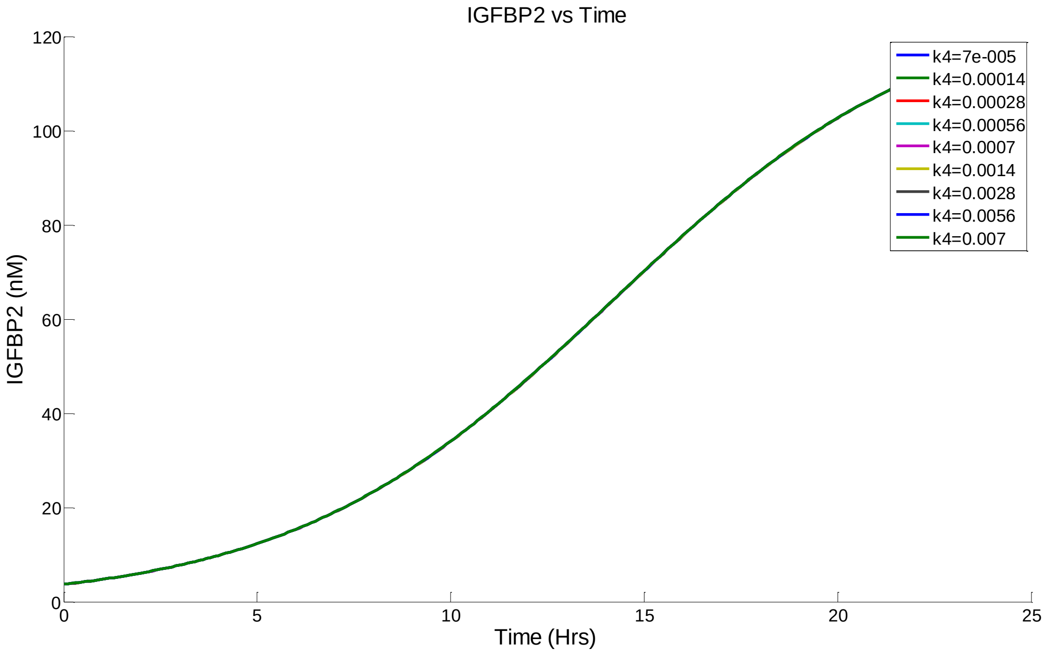
IGFBP2 vs Time
IGFBP2 (nM)
Time (Hrs)
120
100
80
60
40
20
0
0
5
10
15
20
25
k4=7e-005
k4=0.00014
k4=0.00028
k4=0.00056
k4=0.0007
k4=0.0014
k4=0.0028
k4=0.0056
k4=0.007

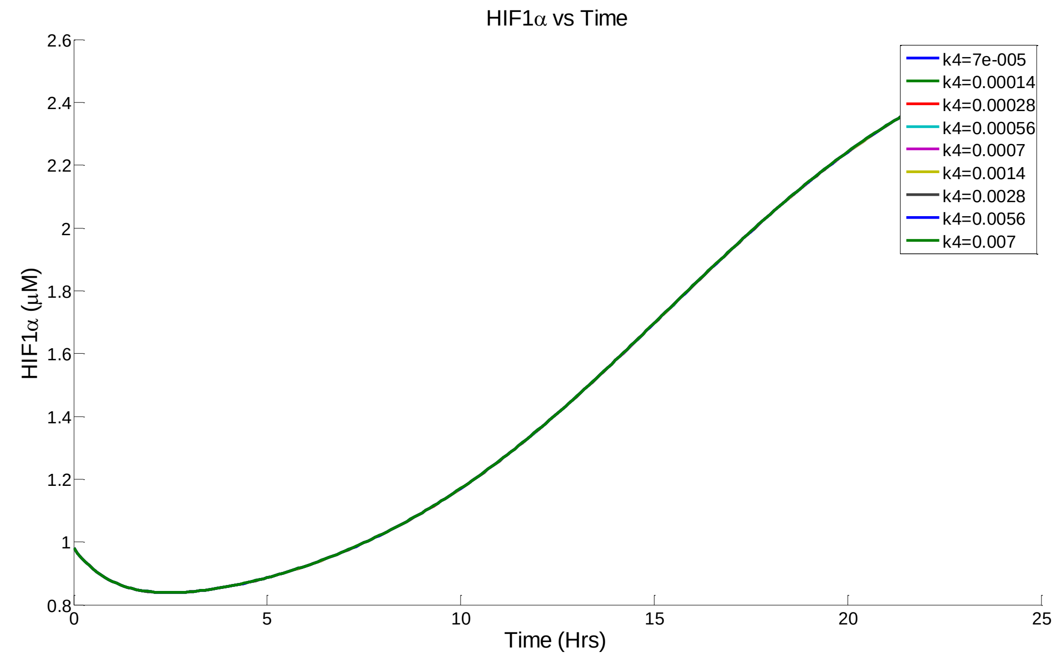

HIF1α vs Time
HIF1α (μM)
Time (Hrs)
k4=7e-005
k4=0.00014
k4=0.00028
k4=0.00056
k4=0.0007
k4=0.0014
k4=0.0028
k4=0.0056
k4=0.007

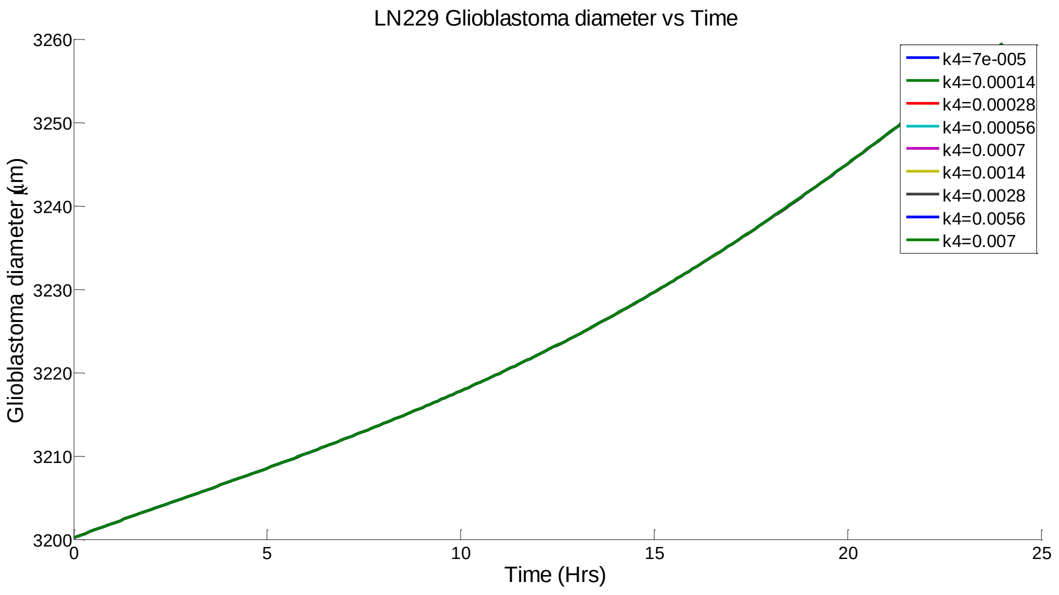

LN229 Glioblastoma diameter vs Time
Glioblastoma diameter (μm)
Time (Hrs)
k4=7e-005
k4=0.00014
k4=0.00028
k4=0.00056
k4=0.0007
k4=0.0014
k4=0.0028
k4=0.0056
k4=0.007

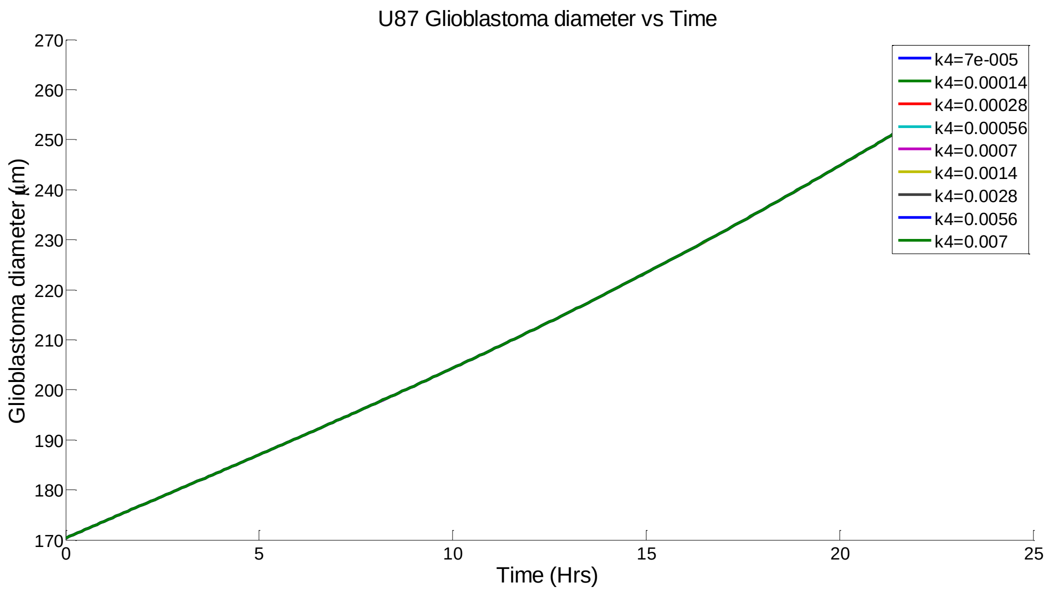

U87 Glioblastoma diameter vs Time
Glioblastoma diameter (μm)
Time (Hrs)
270
260
250
240
230
220
210
200
190
180
170
0
5
10
15
20
25
k4=7e-005
k4=0.00014
k4=0.00028
k4=0.00056
k4=0.0007
k4=0.0014
k4=0.0028
k4=0.0056
k4=0.007

k5

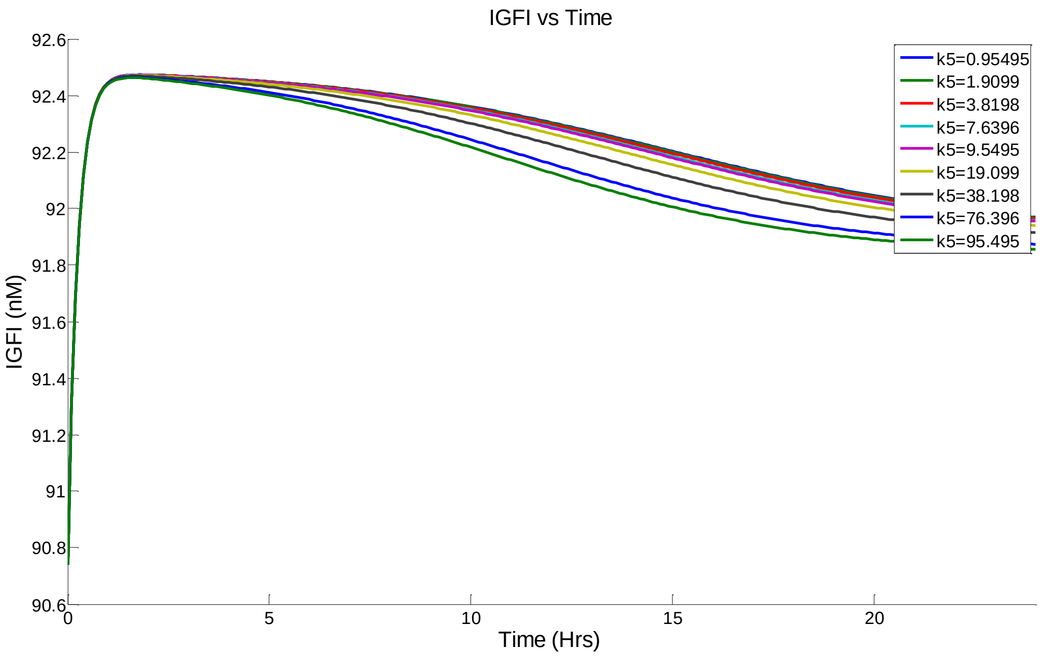

IGFI vs Time
IGFI (nM)
Time (Hrs)
92.6
92.4
92.2
92
91.8
91.6
91.4
91.2
91
90.8
90.6
0
5
10
15
20
k5=0.95495
k5=1.9099
k5=3.8198
k5=7.6396
k5=9.5495
k5=19.099
k5=38.198
k5=76.396
k5=95.495

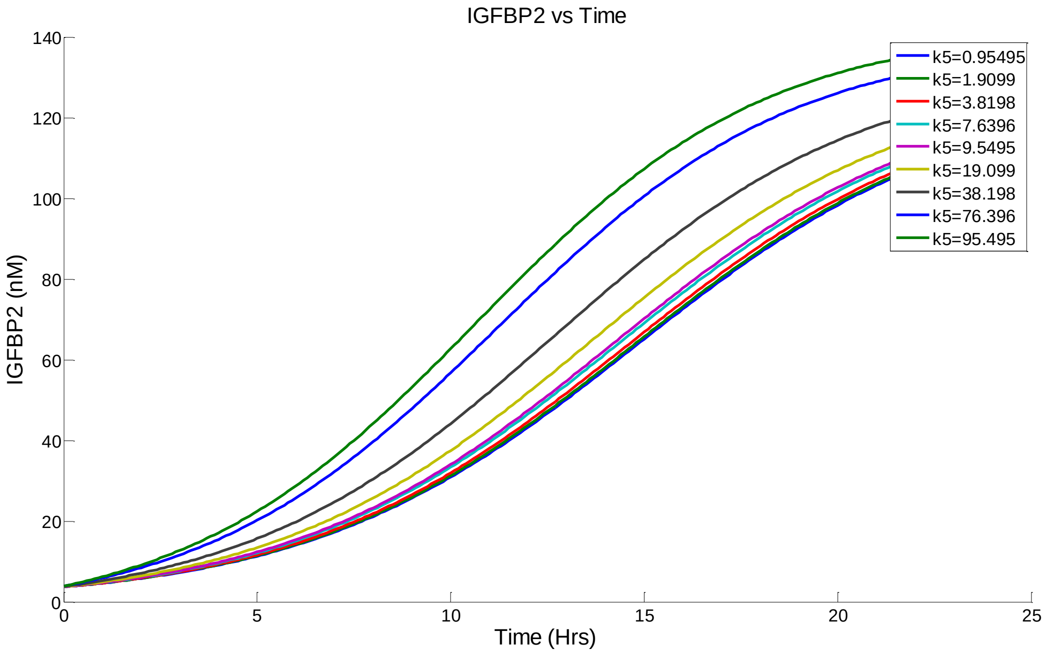
IGFBP2 vs Time
IGFBP2 (nM)
Time (Hrs)
0
20
40
60
80
100
120
140
0
5
10
15
20
25
k5=0.95495
k5=1.9099
k5=3.8198
k5=7.6396
k5=9.5495
k5=19.099
k5=38.198
k5=76.396
k5=95.495

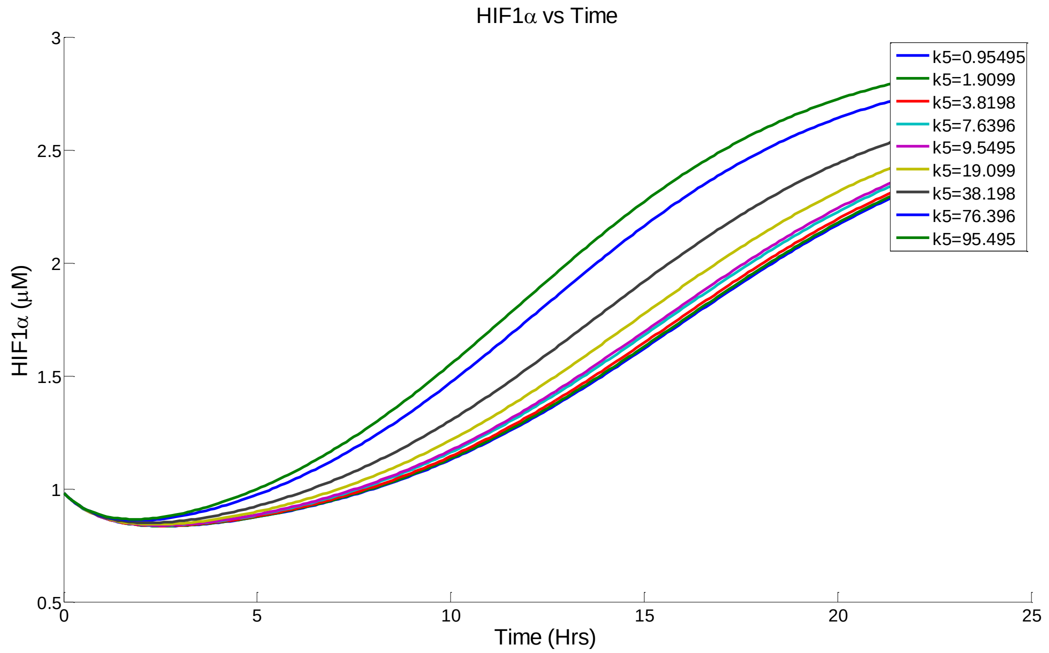
HIF1α vs Time
HIF1α (μM)
Time (Hrs)
0.5
1
1.5
2
2.5
3
0
5
10
15
20
25
k5=0.95495
k5=1.9099
k5=3.8198
k5=7.6396
k5=9.5495
k5=19.099
k5=38.198
k5=76.396
k5=95.495

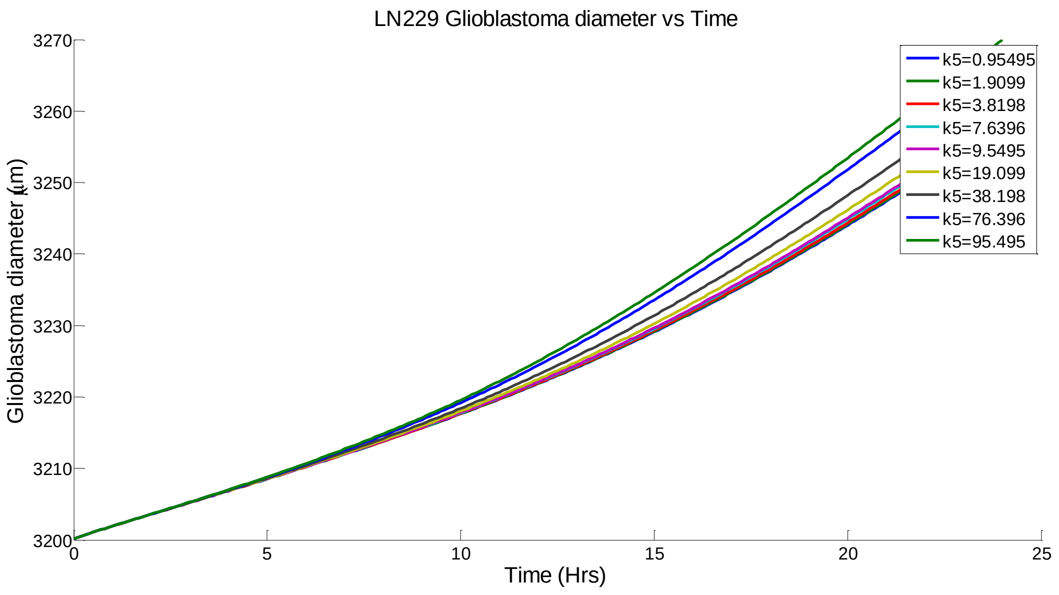
LN229 Glioblastoma diameter vs Time
Glioblastoma diameter (μm)
Time (Hrs)
3200
3210
3220
3230
3240
3250
3260
3270
0
5
10
15
20
25
k5=0.95495
k5=1.9099
k5=3.8198
k5=7.6396
k5=9.5495
k5=19.099
k5=38.198
k5=76.396
k5=95.495

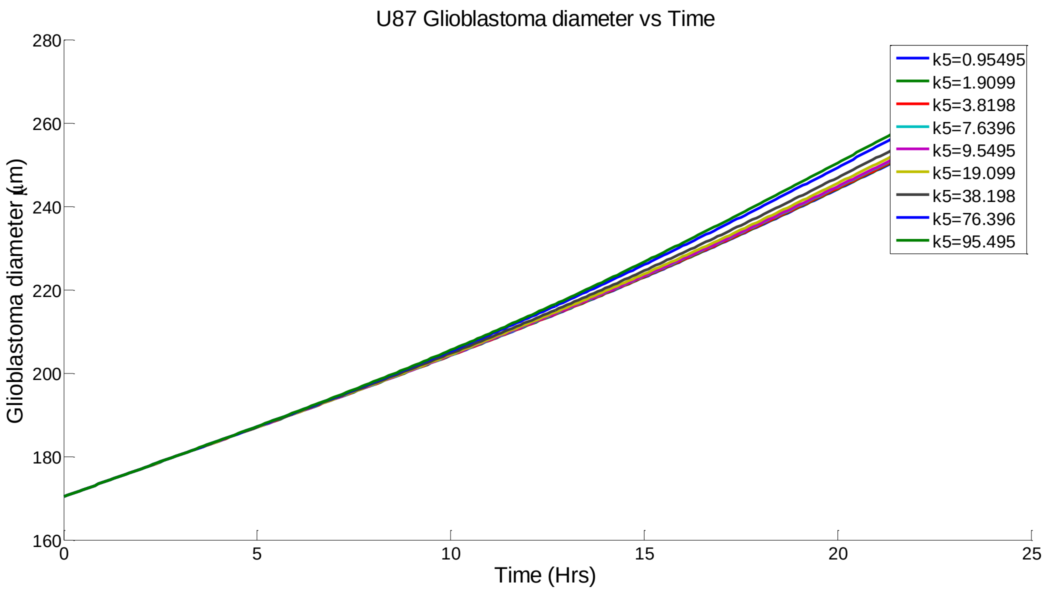
U87 Glioblastoma diameter vs Time
Glioblastoma diameter (μm)
Time (Hrs)
160
180
200
220
240
260
280
0
5
10
15
20
25
k5=0.95495
k5=1.9099
k5=3.8198
k5=7.6396
k5=9.5495
k5=19.099
k5=38.198
k5=76.396
k5=95.495

kd

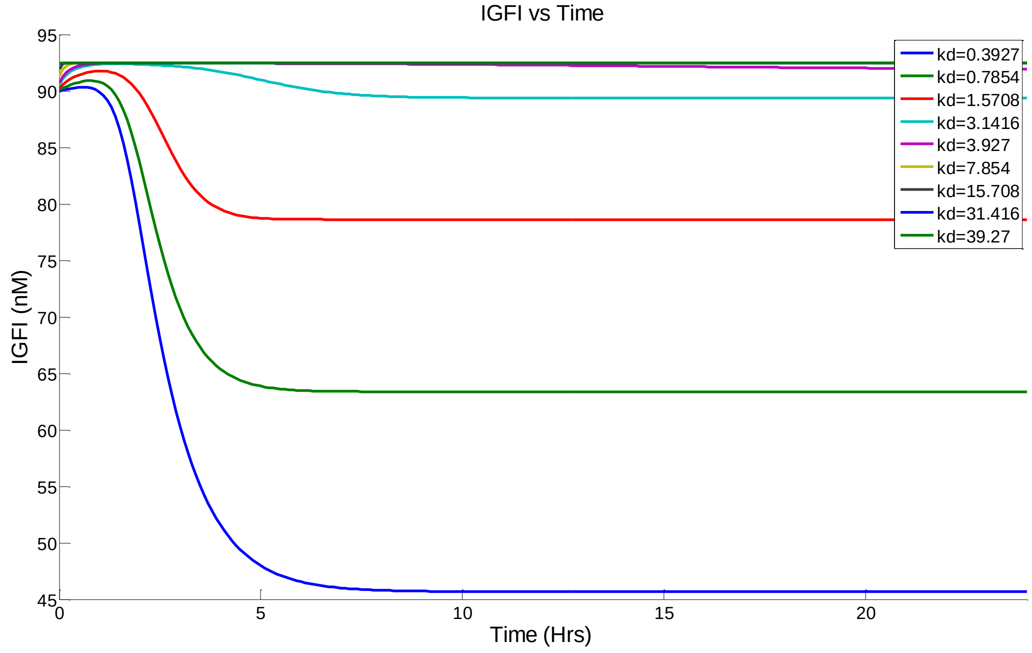
IGFI vs Time
IGFI (nM)
Time (Hrs)
95
90
85
80
75
70
65
60
55
50
45
0
5
10
15
20
kd=0.3927
kd=0.7854
kd=1.5708
kd=3.1416
kd=3.927
kd=7.854
kd=15.708
kd=31.416
kd=39.27

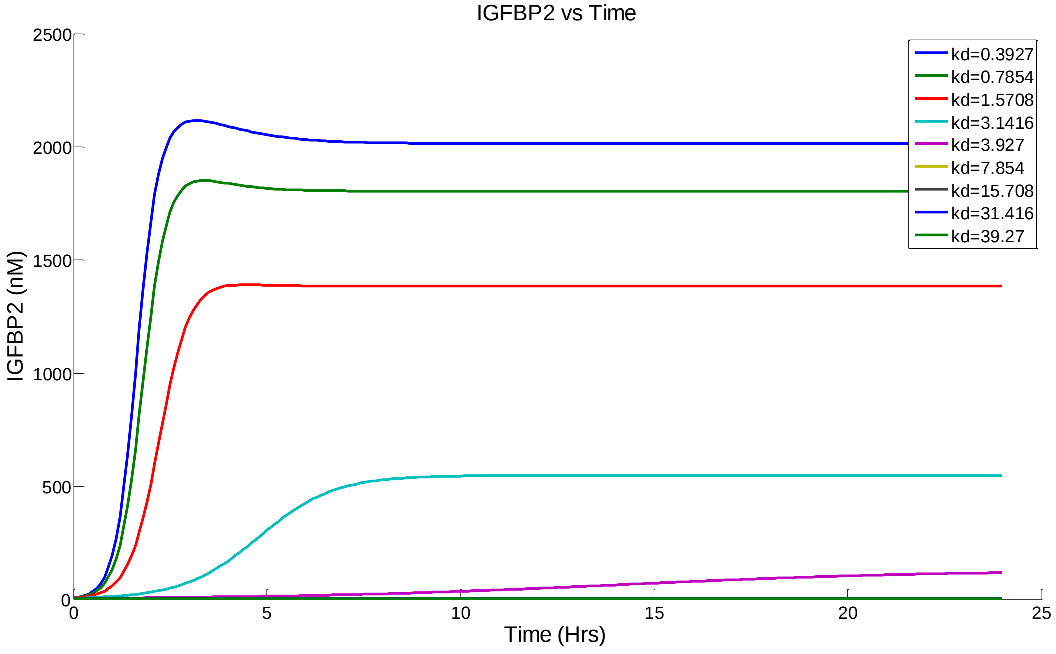
IGFBP2 vs Time
IGFBP2 (nM)
Time (Hrs)
2500
2000
1500
1000
500
0
0
5
10
15
20
25
kd=0.3927
kd=0.7854
kd=1.5708
kd=3.1416
kd=3.927
kd=7.854
kd=15.708
kd=31.416
kd=39.27

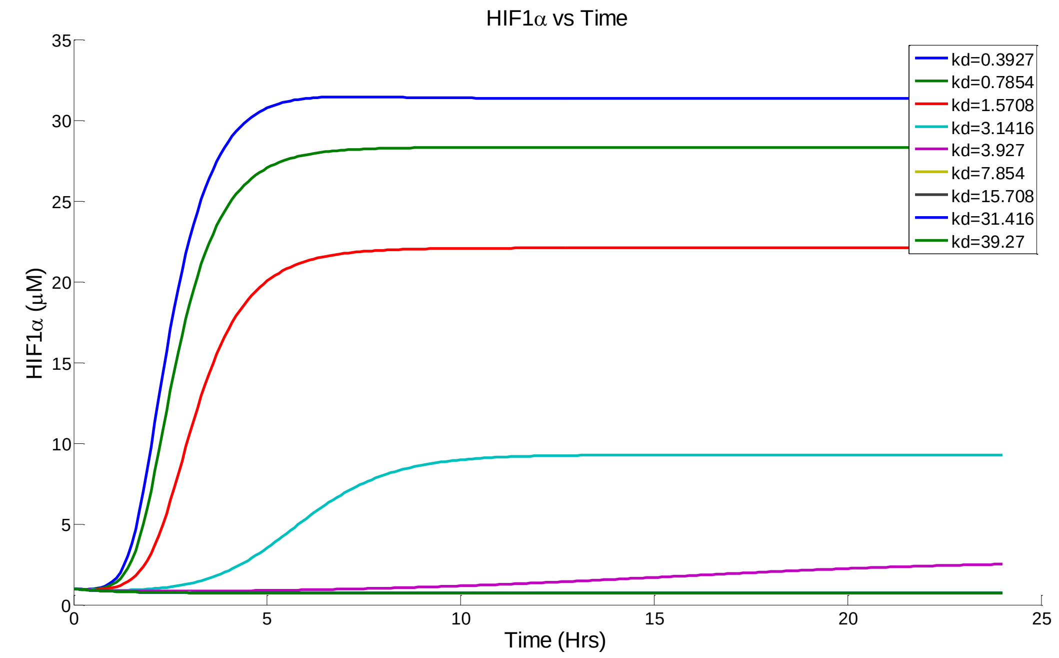
HIF1α vs Time
HIF1α (μM)
Time (Hrs)
kd=0.3927
kd=0.7854
kd=1.5708
kd=3.1416
kd=3.927
kd=7.854
kd=15.708
kd=31.416
kd=39.27

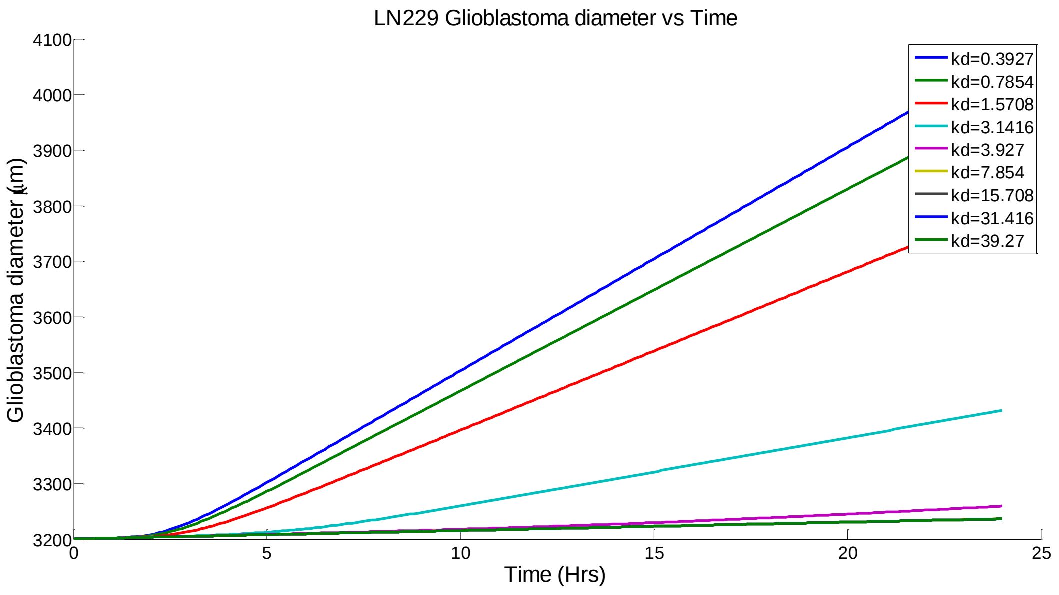
LN229 Glioblastoma diameter vs Time
Glioblastoma diameter (μm)
Time (Hrs)
kd=0.3927
kd=0.7854
kd=1.5708
kd=3.1416
kd=3.927
kd=7.854
kd=15.708
kd=31.416
kd=39.27

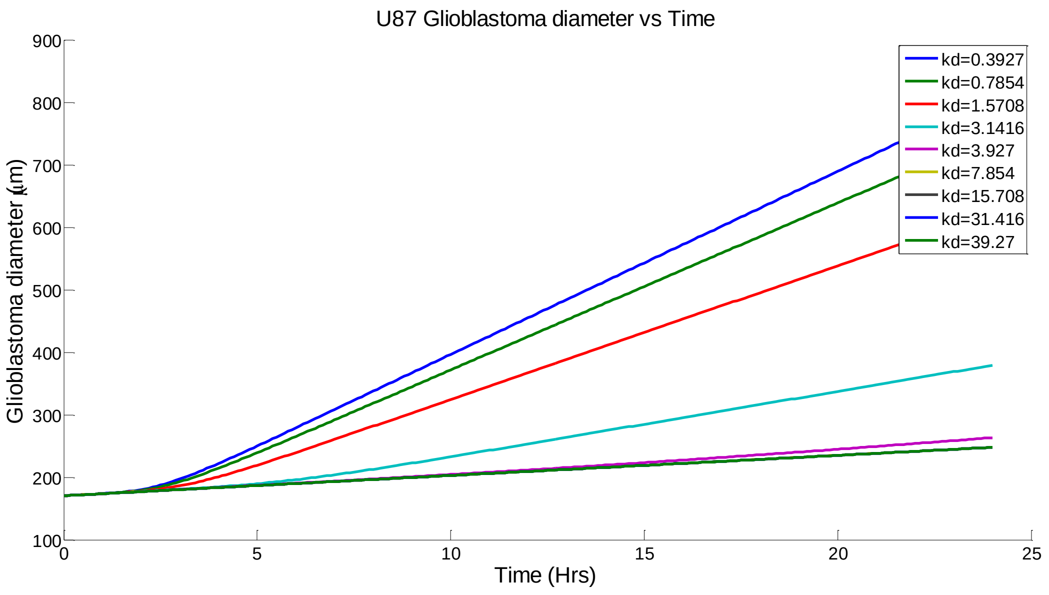
U87 Glioblastoma diameter vs Time
Glioblastoma diameter (μm)
Time (Hrs)
kd=0.3927
kd=0.7854
kd=1.5708
kd=3.1416
kd=3.927
kd=7.854
kd=15.708
kd=31.416
kd=39.27

k6

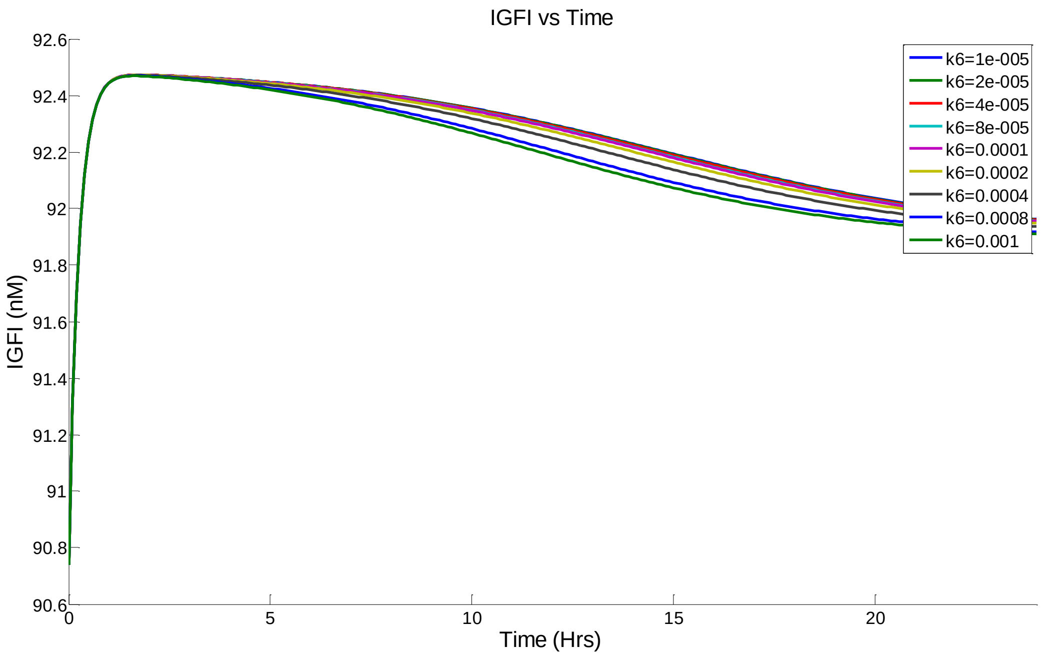
IGFI vs Time
IGFI (nM)
Time (Hrs)
k6=1e-005
k6=2e-005
k6=4e-005
k6=8e-005
k6=0.0001
k6=0.0002
k6=0.0004
k6=0.0008
k6=0.001

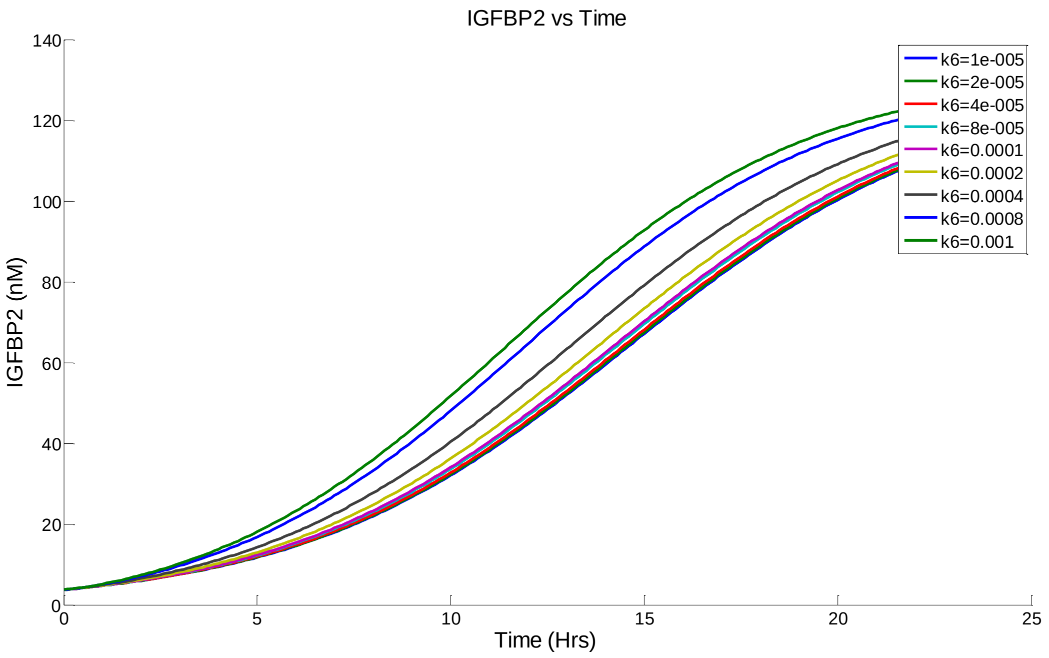
IGFBP2 vs Time
IGFBP2 (nM)
Time (Hrs)
0
20
40
60
80
100
120
140
0
5
10
15
20
25
k6=1e-005
k6=2e-005
k6=4e-005
k6=8e-005
k6=0.0001
k6=0.0002
k6=0.0004
k6=0.0008
k6=0.001

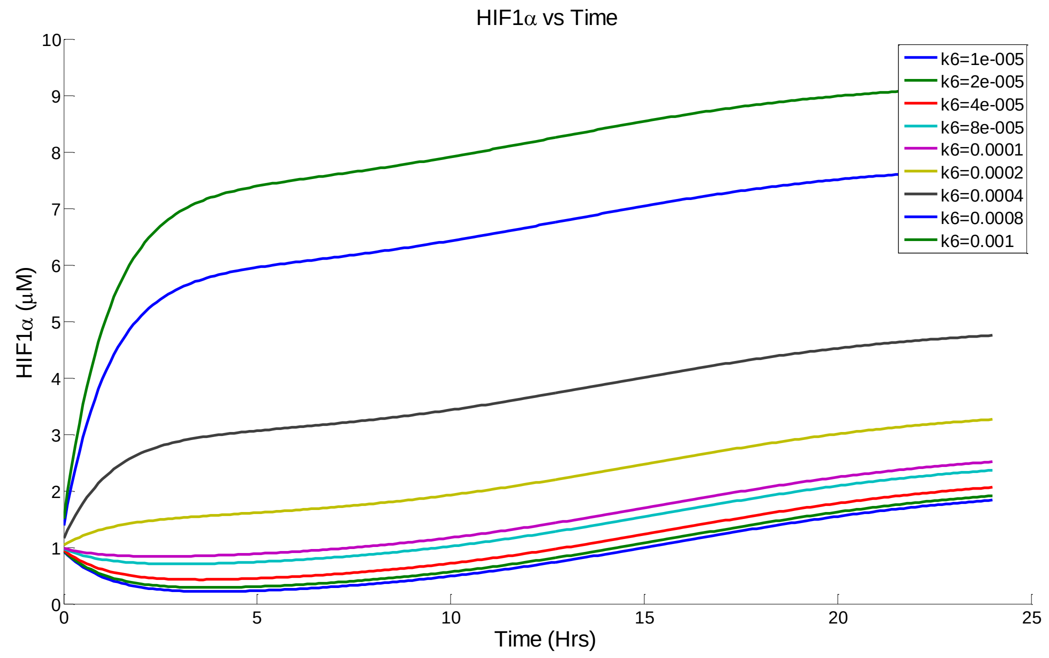
HIF1α vs Time
HIF1α (μM)
Time (Hrs)
0
1
2
3
4
5
6
7
8
9
10
0
5
10
15
20
25
k6=1e-005
k6=2e-005
k6=4e-005
k6=8e-005
k6=0.0001
k6=0.0002
k6=0.0004
k6=0.0008
k6=0.001

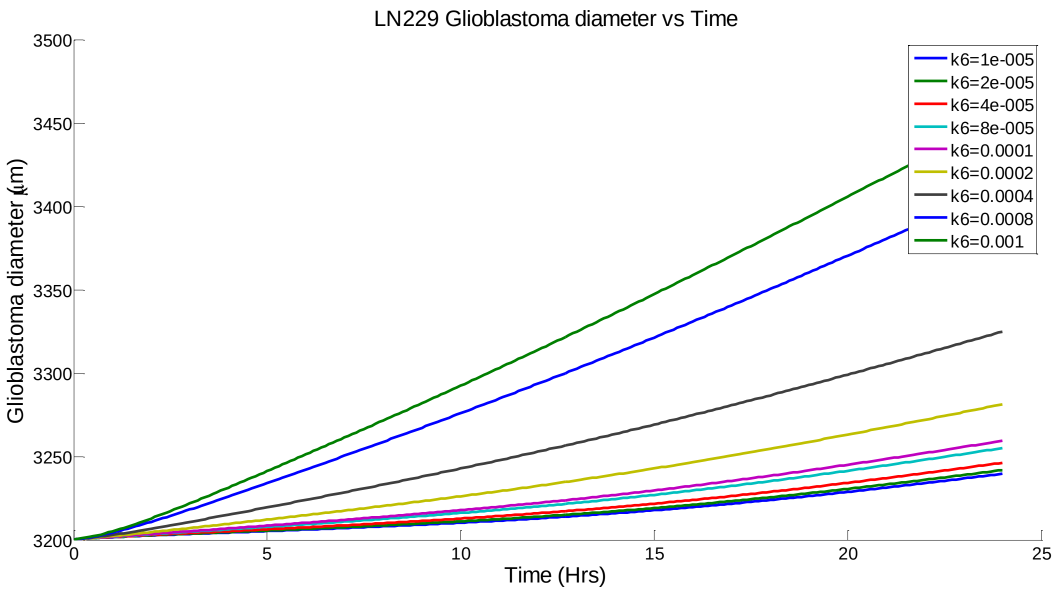
LN229 Glioblastoma diameter vs Time
Glioblastoma diameter (μm)
Time (Hrs)
3500
3450
3400
3350
3300
3250
3200
0
5
10
15
20
25
k6=1e-005
k6=2e-005
k6=4e-005
k6=8e-005
k6=0.0001
k6=0.0002
k6=0.0004
k6=0.0008
k6=0.001

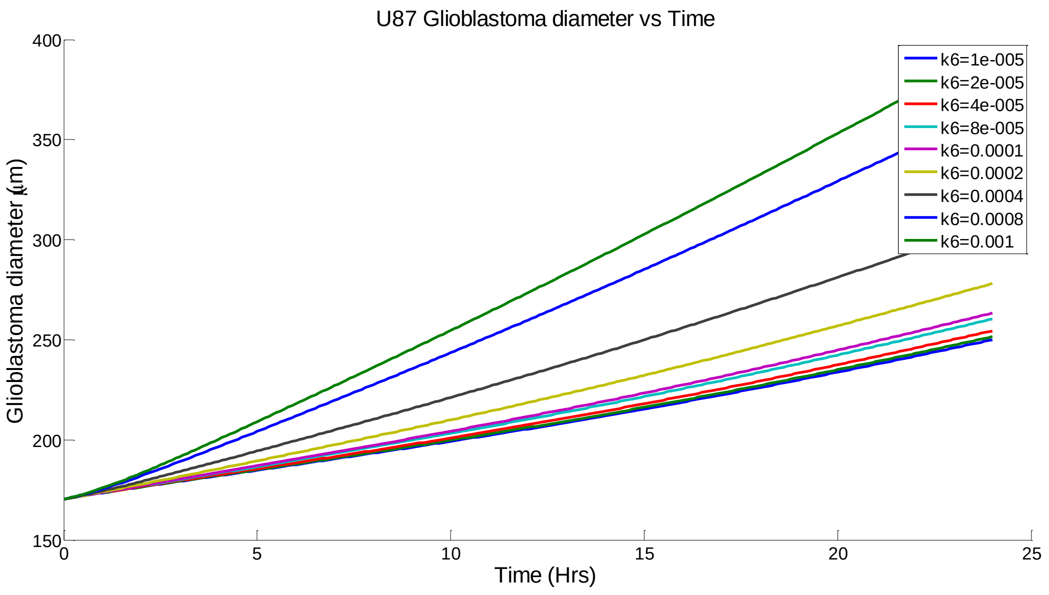
U87 Glioblastoma diameter vs Time
Glioblastoma diameter (μm)
Time (Hrs)
400
350
300
250
200
150
0
5
10
15
20
25
k6=1e-005
k6=2e-005
k6=4e-005
k6=8e-005
k6=0.0001
k6=0.0002
k6=0.0004
k6=0.0008
k6=0.001

k7

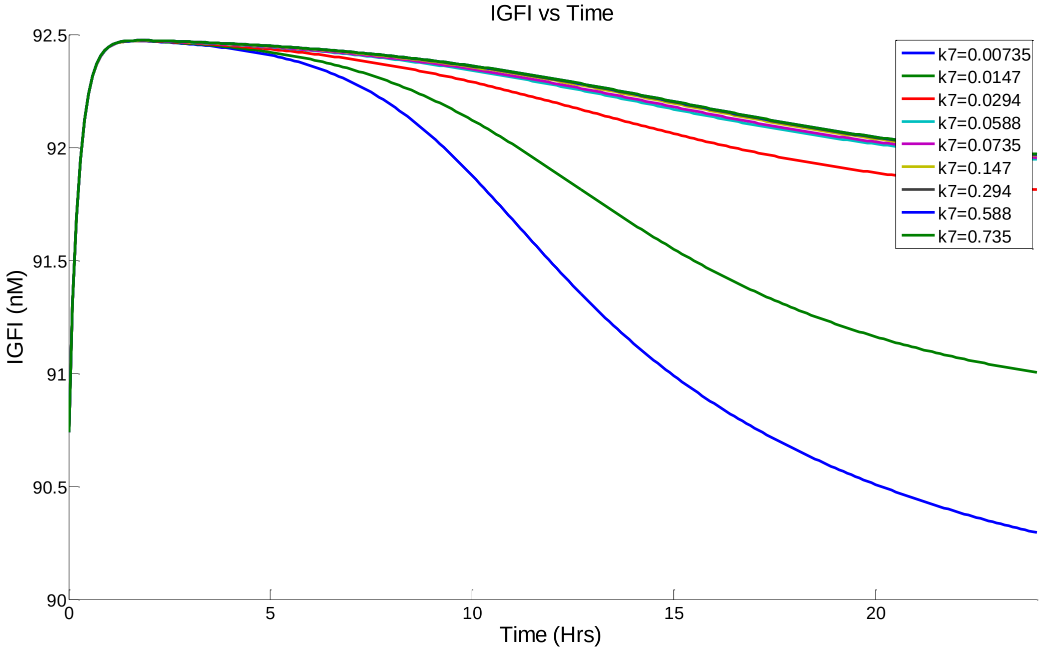

IGFI vs Time
92.5
92
91.5
91
90.5
90
IGFI (nM)
0
5
10
15
20
Time (Hrs)
k7=0.00735
k7=0.0147
k7=0.0294
k7=0.0588
k7=0.0735
k7=0.147
k7=0.294
k7=0.588
k7=0.735

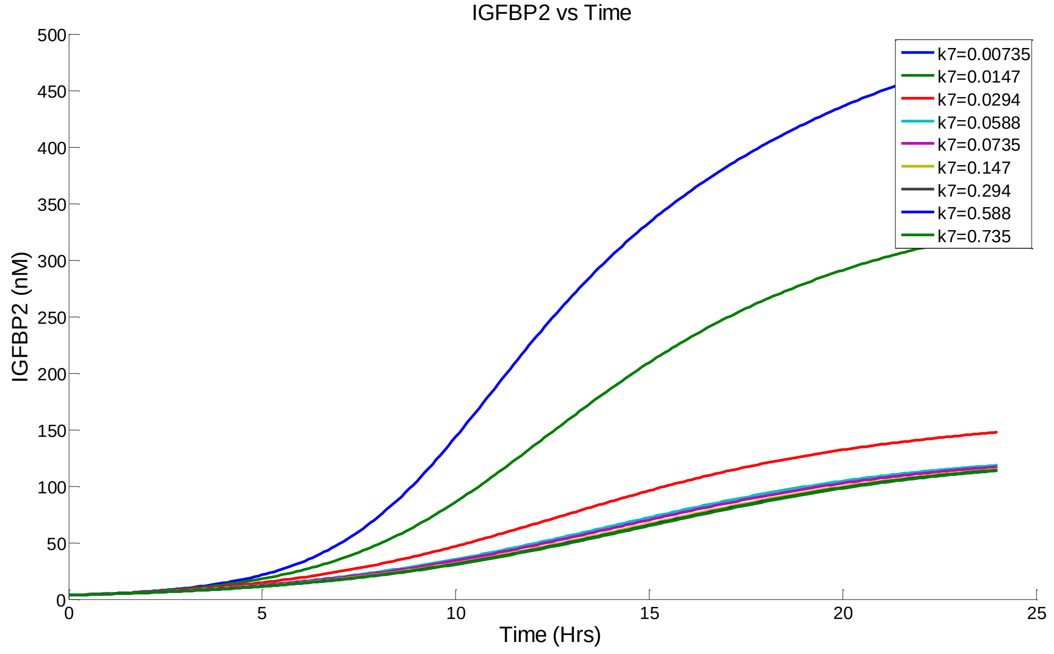

IGFBP2 vs Time
500
450
400
350
300
250
200
150
100
50
0
IGFBP2 (nM)
0
5
10
15
20
25
Time (Hrs)
k7=0.00735
k7=0.0147
k7=0.0294
k7=0.0588
k7=0.0735
k7=0.147
k7=0.294
k7=0.588
k7=0.735

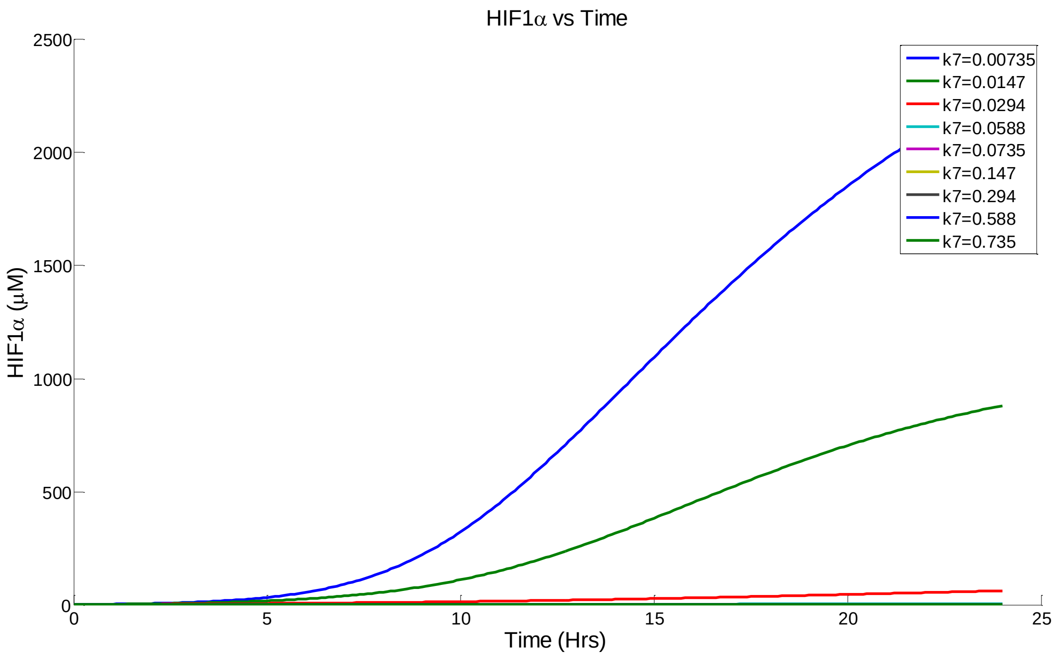
HIF1α vs Time
2500
2000
1500
1000
500
0
HIF1α (μM)
0
5
10
15
20
25
Time (Hrs)
k7=0.00735
k7=0.0147
k7=0.0294
k7=0.0588
k7=0.0735
k7=0.147
k7=0.294
k7=0.588
k7=0.735

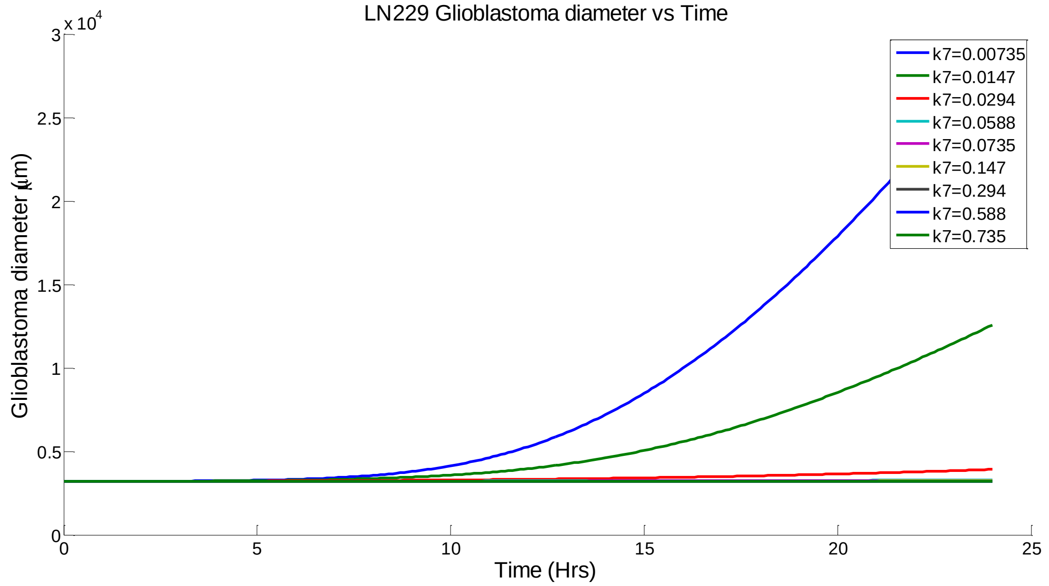
LN229 Glioblastoma diameter vs Time
x 10^4
3
2.5
2
1.5
1
0.5
0
Glioblastoma diameter (μm)
0
5
10
15
20
25
Time (Hrs)
k7=0.00735
k7=0.0147
k7=0.0294
k7=0.0588
k7=0.0735
k7=0.147
k7=0.294
k7=0.588
k7=0.735

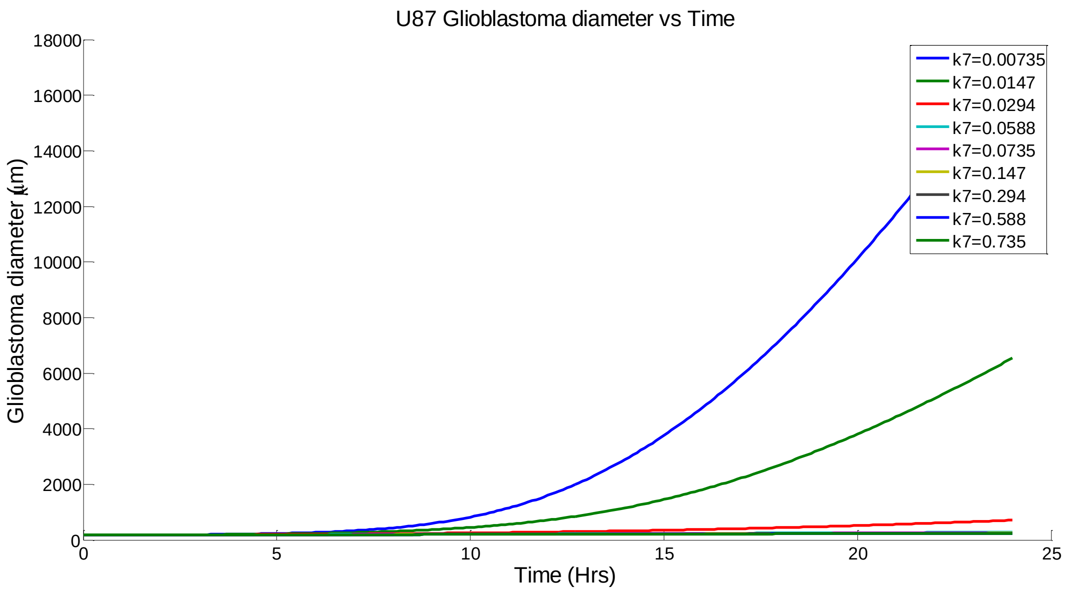
U87 Glioblastoma diameter vs Time
Glioblastoma diameter (μm)
Time (Hrs)
k7=0.00735
k7=0.0147
k7=0.0294
k7=0.0588
k7=0.0735
k7=0.147
k7=0.294
k7=0.588
k7=0.735
0
2000
4000
6000
8000
10000
12000
14000
16000
18000
5
10
15
20
25

k8

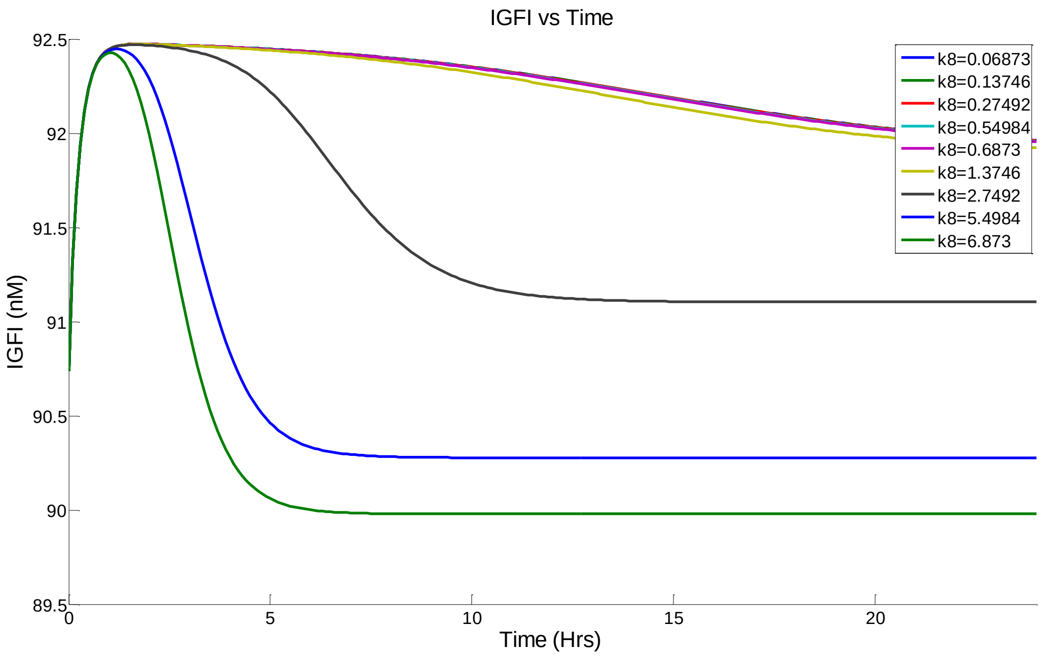
IGFI vs Time
IGFI (nM)
Time (Hrs)
k8=0.06873
k8=0.13746
k8=0.27492
k8=0.54984
k8=0.6873
k8=1.3746
k8=2.7492
k8=5.4984
k8=6.873
89.5
90
90.5
91
91.5
92
92.5
0
5
10
15
20

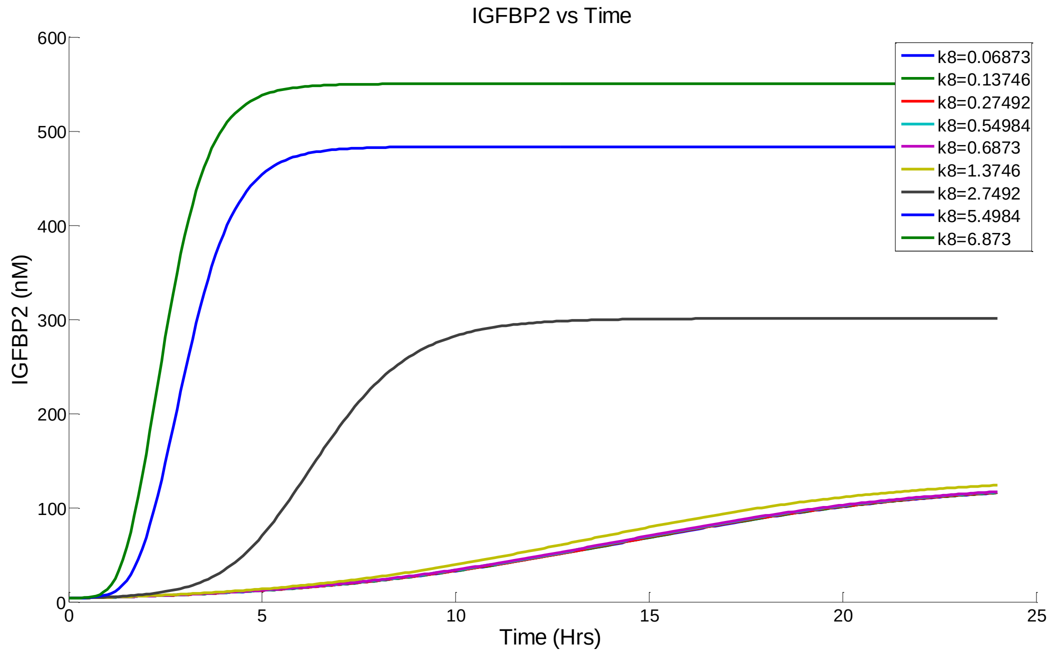
IGFBP2 vs Time
IGFBP2 (nM)
Time (Hrs)
0
100
200
300
400
500
600
0
5
10
15
20
25
k8=0.06873
k8=0.13746
k8=0.27492
k8=0.54984
k8=0.6873
k8=1.3746
k8=2.7492
k8=5.4984
k8=6.873

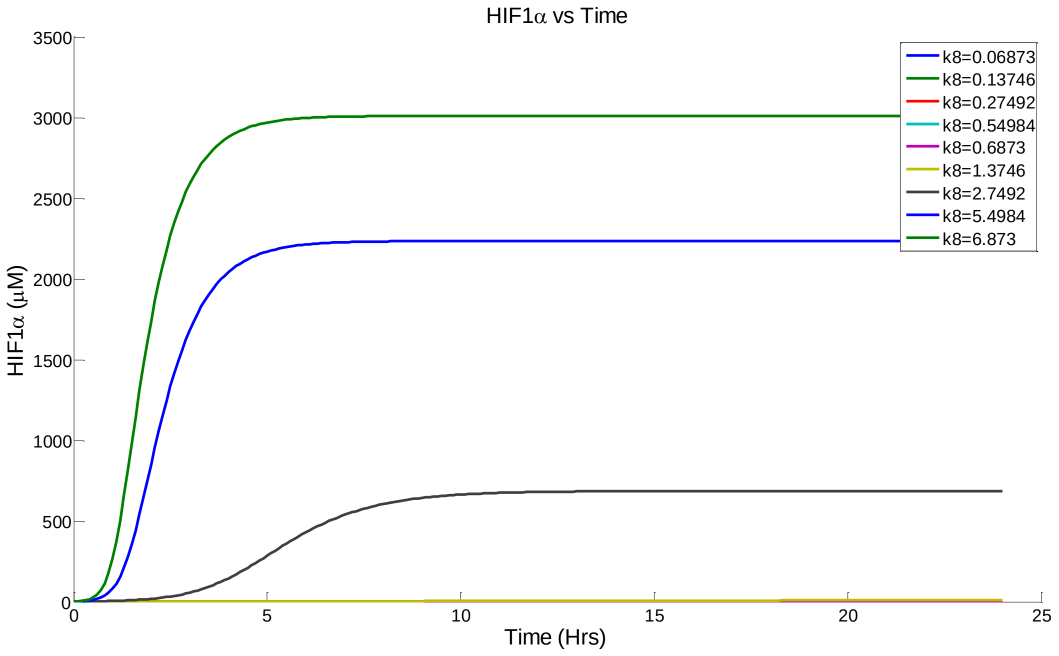
HIF1α vs Time
HIF1α (μM)
Time (Hrs)
0
500
1000
1500
2000
2500
3000
3500
0
5
10
15
20
25
k8=0.06873
k8=0.13746
k8=0.27492
k8=0.54984
k8=0.6873
k8=1.3746
k8=2.7492
k8=5.4984
k8=6.873

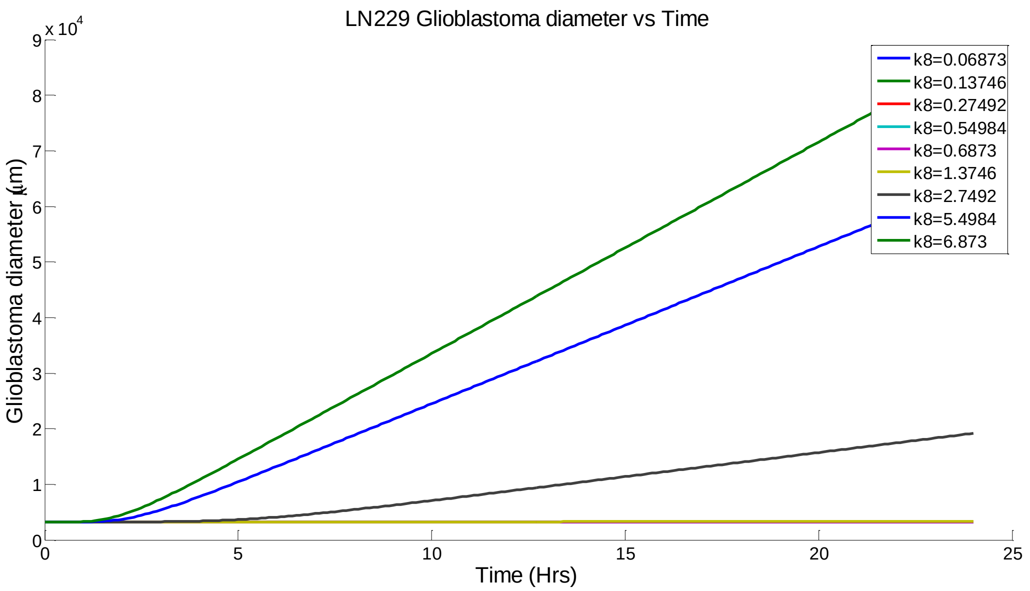

LN229 Glioblastoma diameter vs Time
x 10^4
9
8
7
6
5
4
3
2
1
0
Glioblastoma diameter (μm)
0
5
10
15
20
25
Time (Hrs)
k8=0.06873
k8=0.13746
k8=0.27492
k8=0.54984
k8=0.6873
k8=1.3746
k8=2.7492
k8=5.4984
k8=6.873

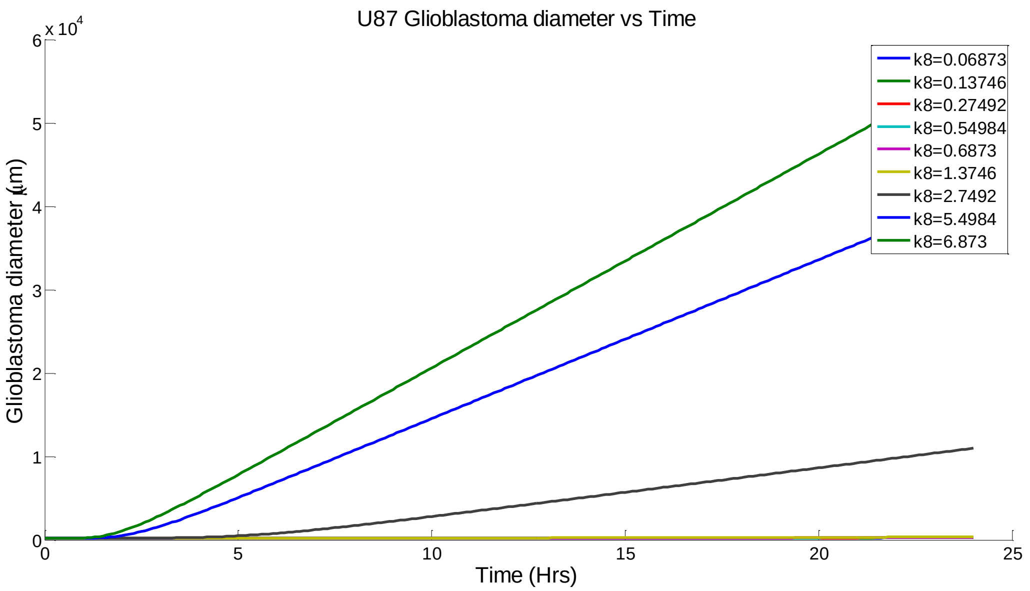

U87 Glioblastoma diameter vs Time
x 10^4
6
5
4
3
2
1
0
Glioblastoma diameter (μm)
0
5
10
15
20
25
Time (Hrs)
k8=0.06873
k8=0.13746
k8=0.27492
k8=0.54984
k8=0.6873
k8=1.3746
k8=2.7492
k8=5.4984
k8=6.873

k9

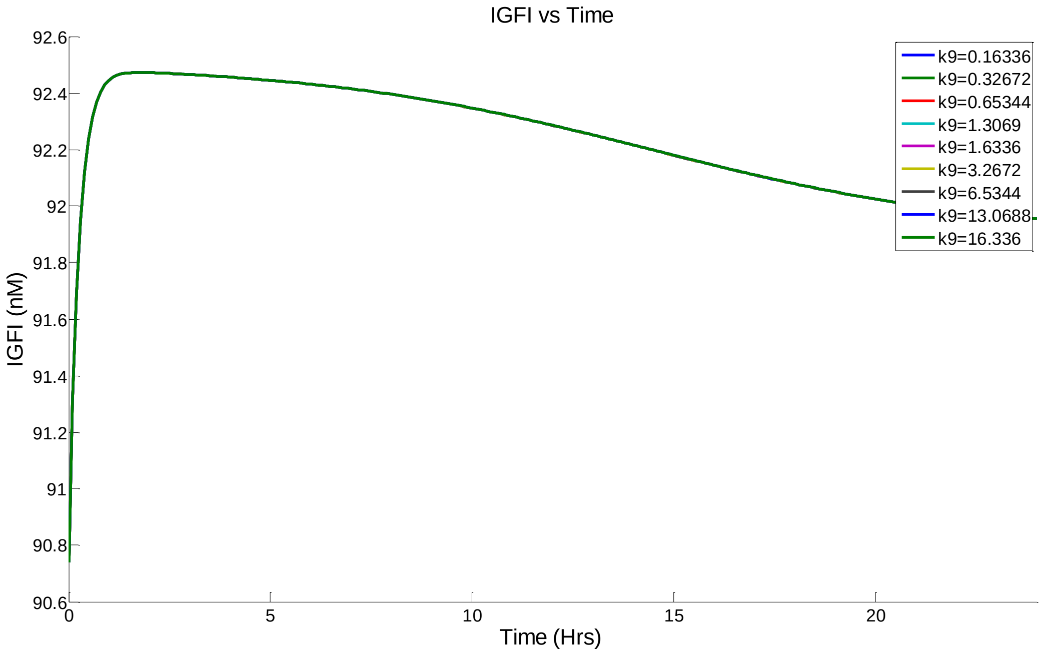
IGFI vs Time
IGFI (nM)
Time (Hrs)
92.6
92.4
92.2
92
91.8
91.6
91.4
91.2
91
90.8
90.6
0
5
10
15
20
k9=0.16336
k9=0.32672
k9=0.65344
k9=1.3069
k9=1.6336
k9=3.2672
k9=6.5344
k9=13.0688
k9=16.336

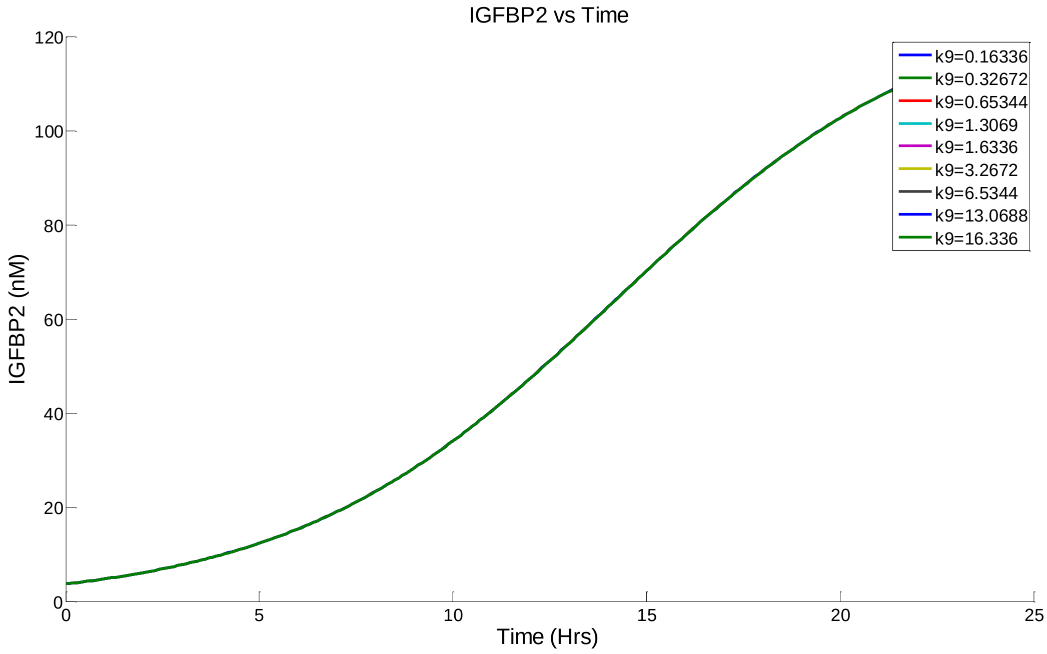
IGFBP2 vs Time
IGFBP2 (nM)
Time (Hrs)
120
100
80
60
40
20
0
0
5
10
15
20
25
k9=0.16336
k9=0.32672
k9=0.65344
k9=1.3069
k9=1.6336
k9=3.2672
k9=6.5344
k9=13.0688
k9=16.336

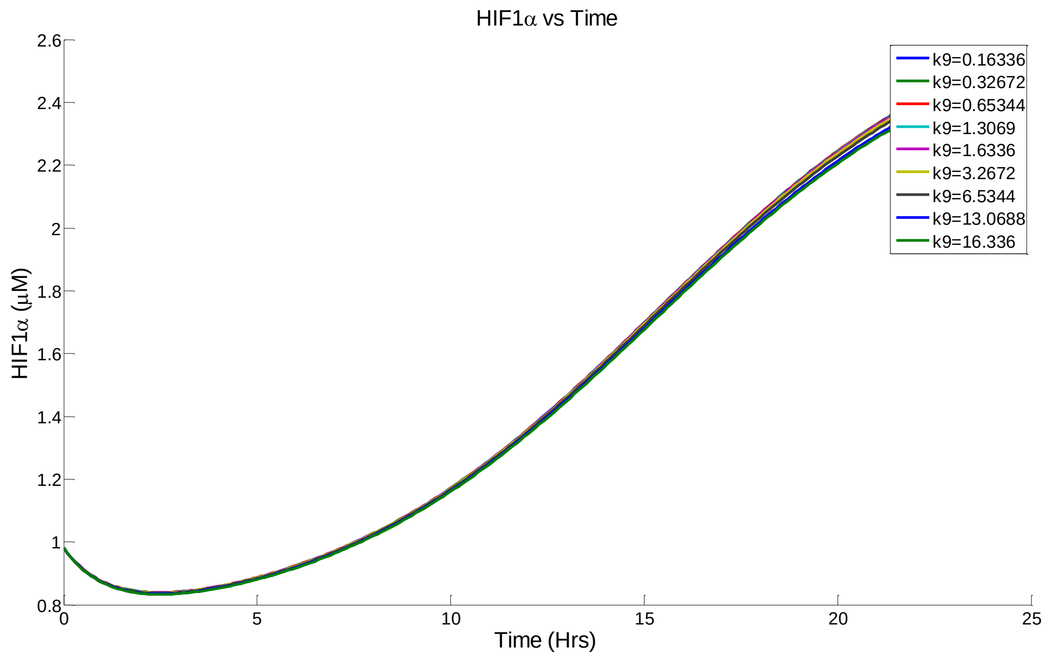
HIF1α vs Time
HIF1α (μM)
Time (Hrs)
k9=0.16336
k9=0.32672
k9=0.65344
k9=1.3069
k9=1.6336
k9=3.2672
k9=6.5344
k9=13.0688
k9=16.336

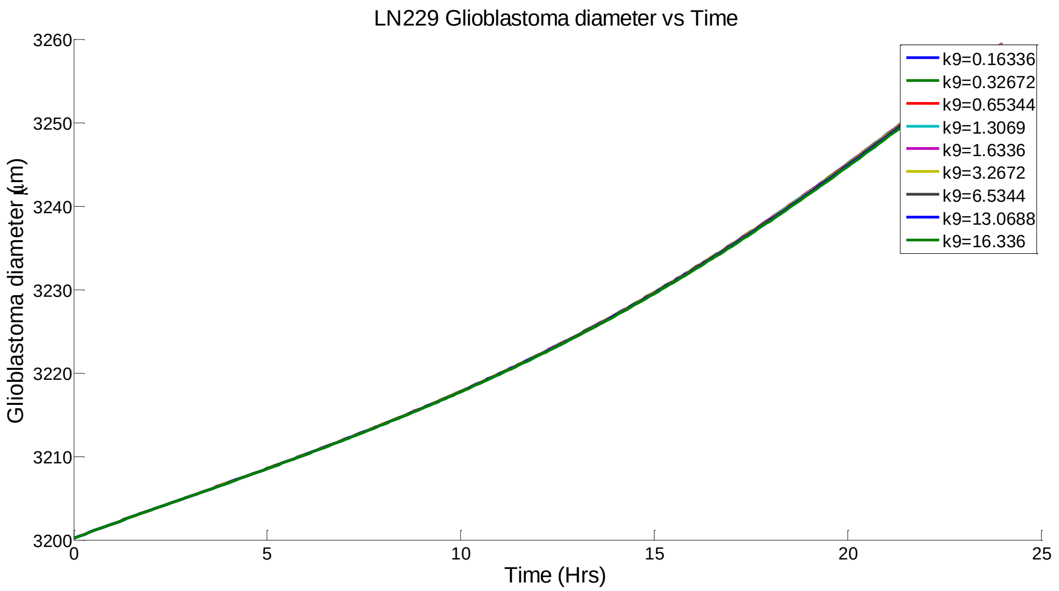
LN229 Glioblastoma diameter vs Time
Glioblastoma diameter (μm)
Time (Hrs)
k9=0.16336
k9=0.32672
k9=0.65344
k9=1.3069
k9=1.6336
k9=3.2672
k9=6.5344
k9=13.0688
k9=16.336

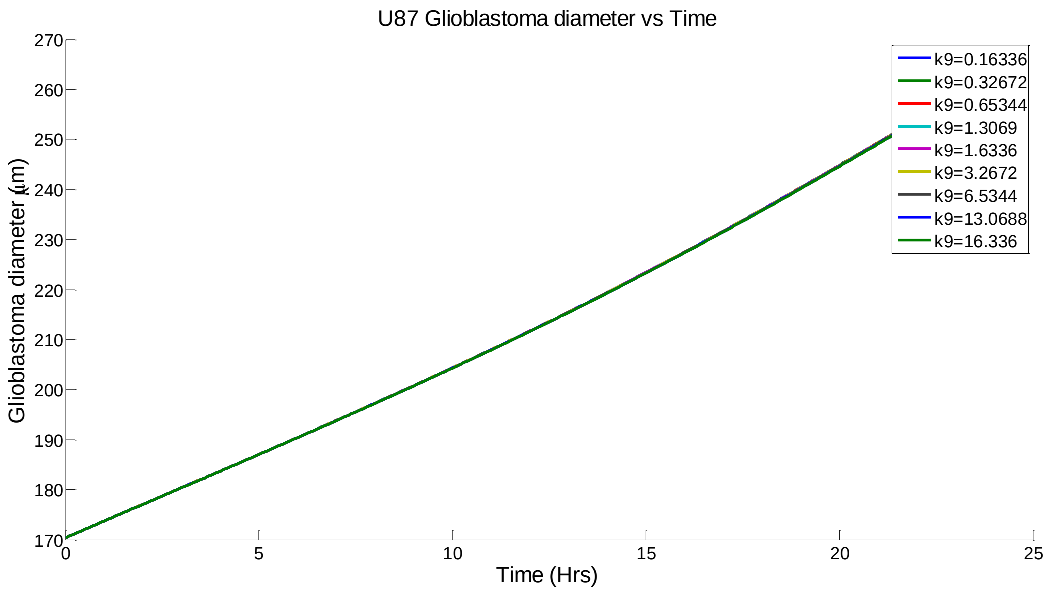
U87 Glioblastoma diameter vs Time
Glioblastoma diameter (μm)
Time (Hrs)
k9=0.16336
k9=0.32672
k9=0.65344
k9=1.3069
k9=1.6336
k9=3.2672
k9=6.5344
k9=13.0688
k9=16.336

k10

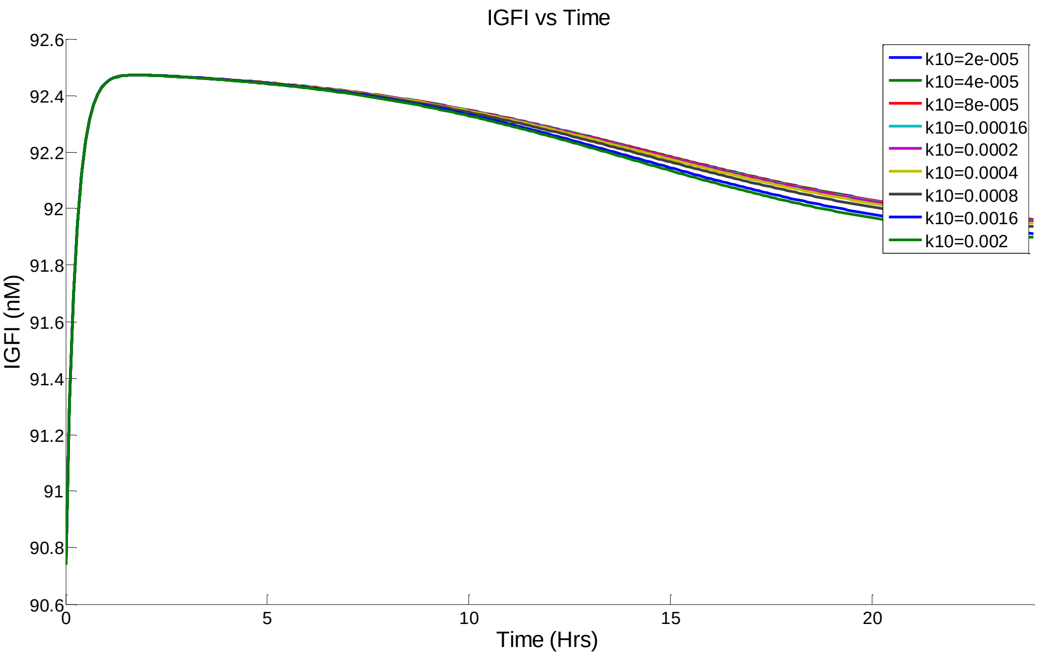
IGFI vs Time
IGFI (nM)
Time (Hrs)
k10=2e-005
k10=4e-005
k10=8e-005
k10=0.00016
k10=0.0002
k10=0.0004
k10=0.0008
k10=0.0016
k10=0.002

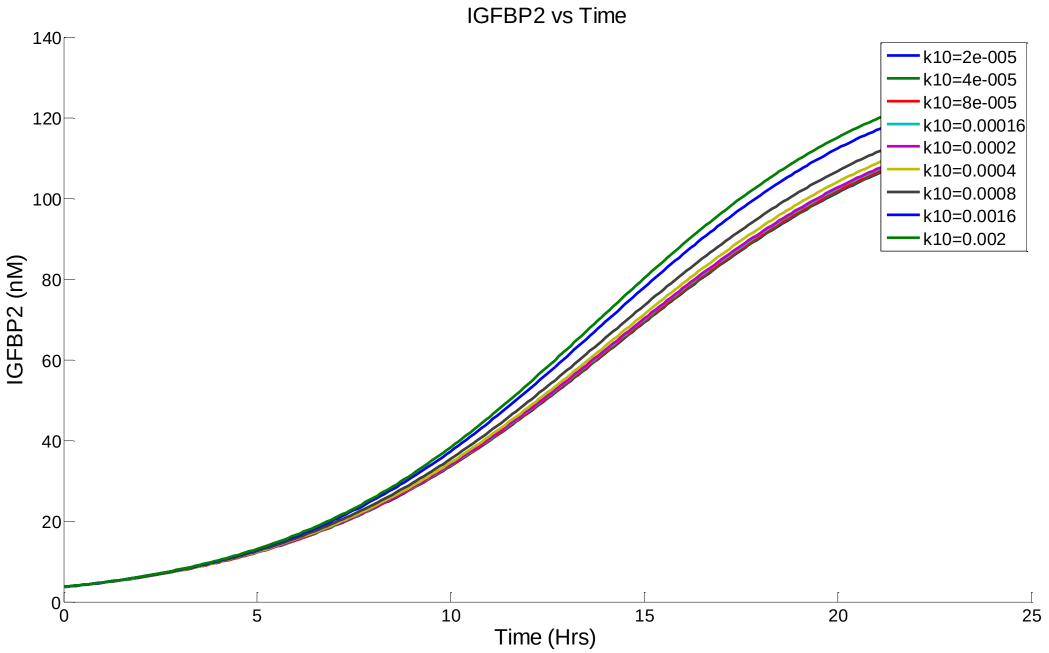
IGFBP2 vs Time
IGFBP2 (nM)
Time (Hrs)
0
20
40
60
80
100
120
140
0
5
10
15
20
25
k10=2e-005
k10=4e-005
k10=8e-005
k10=0.00016
k10=0.0002
k10=0.0004
k10=0.0008
k10=0.0016
k10=0.002

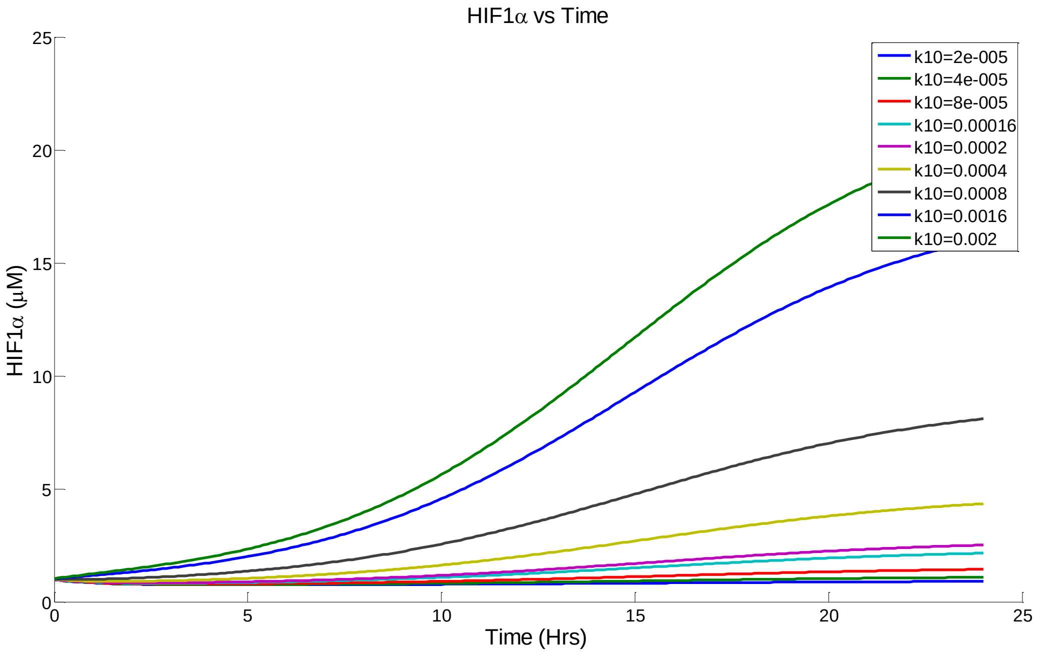
HIF1α vs Time
HIF1α (μM)
Time (Hrs)
0
5
10
15
20
25
0
5
10
15
20
25
k10=2e-005
k10=4e-005
k10=8e-005
k10=0.00016
k10=0.0002
k10=0.0004
k10=0.0008
k10=0.0016
k10=0.002

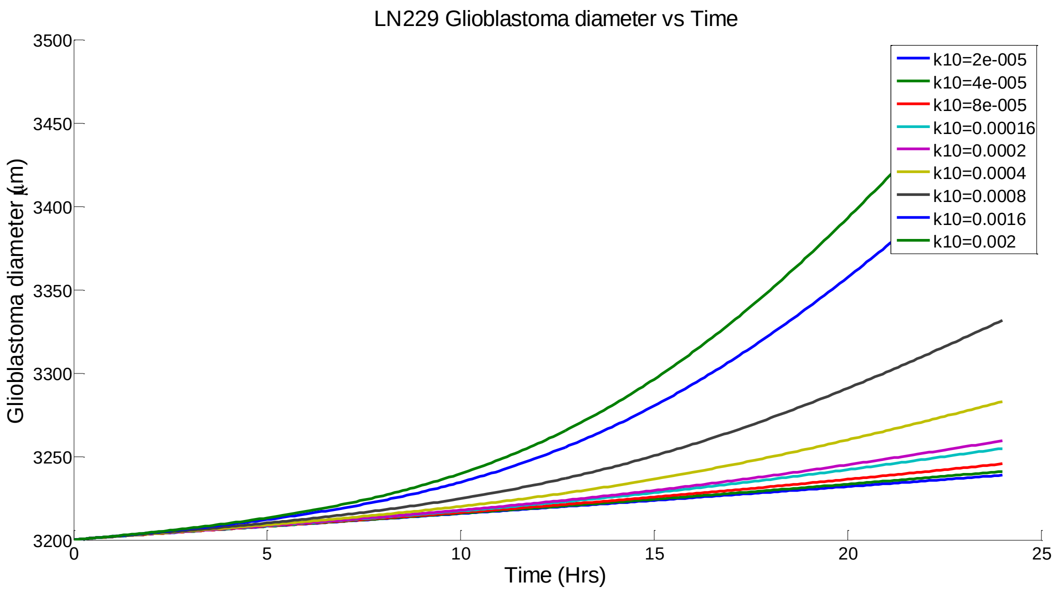
LN229 Glioblastoma diameter vs Time
Glioblastoma diameter (μm)
Time (Hrs)
3500
3450
3400
3350
3300
3250
3200
0
5
10
15
20
25
k10=2e-005
k10=4e-005
k10=8e-005
k10=0.00016
k10=0.0002
k10=0.0004
k10=0.0008
k10=0.0016
k10=0.002

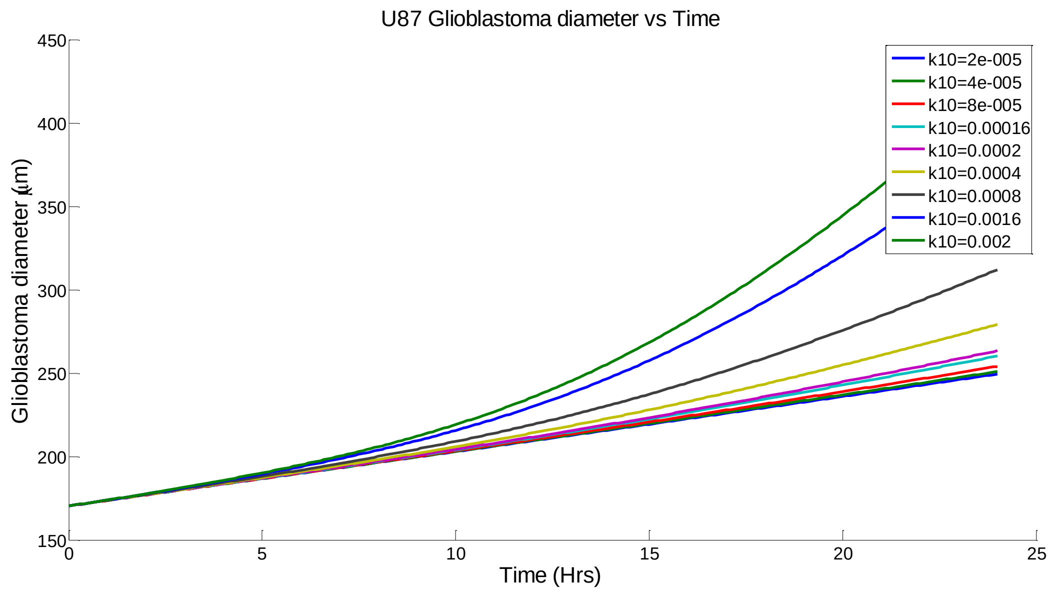
U87 Glioblastoma diameter vs Time
Glioblastoma diameter (μm)
Time (Hrs)
450
400
350
300
250
200
150
0
5
10
15
20
25
k10=2e-005
k10=4e-005
k10=8e-005
k10=0.00016
k10=0.0002
k10=0.0004
k10=0.0008
k10=0.0016
k10=0.002

v1

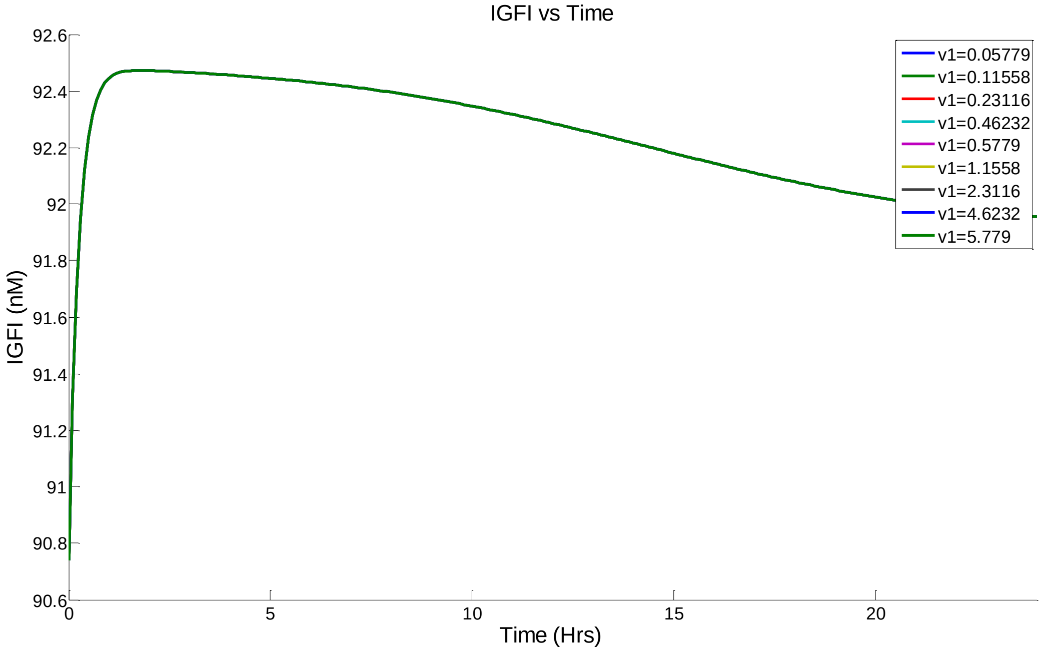

IGFI vs Time
IGFI (nM)
Time (Hrs)
v1=0.05779
v1=0.11558
v1=0.23116
v1=0.46232
v1=0.5779
v1=1.1558
v1=2.3116
v1=4.6232
v1=5.779

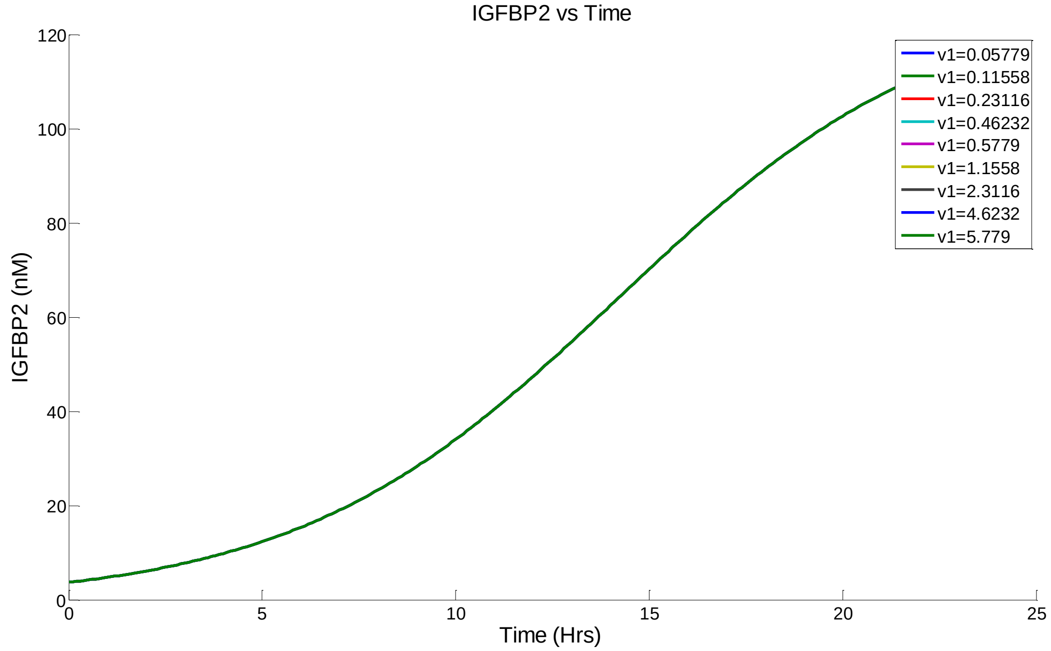

IGFBP2 vs Time
IGFBP2 (nM)
Time (Hrs)
v1=0.05779
v1=0.11558
v1=0.23116
v1=0.46232
v1=0.5779
v1=1.1558
v1=2.3116
v1=4.6232
v1=5.779

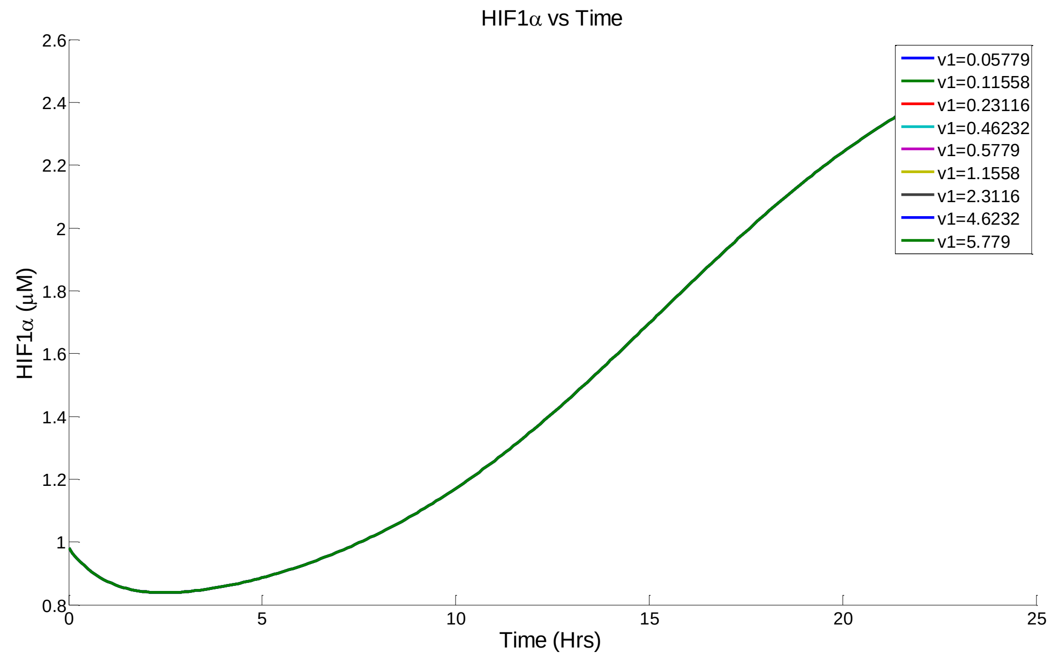
HIF1α vs Time
HIF1α (μM)
Time (Hrs)
v1=0.05779
v1=0.11558
v1=0.23116
v1=0.46232
v1=0.5779
v1=1.1558
v1=2.3116
v1=4.6232
v1=5.779

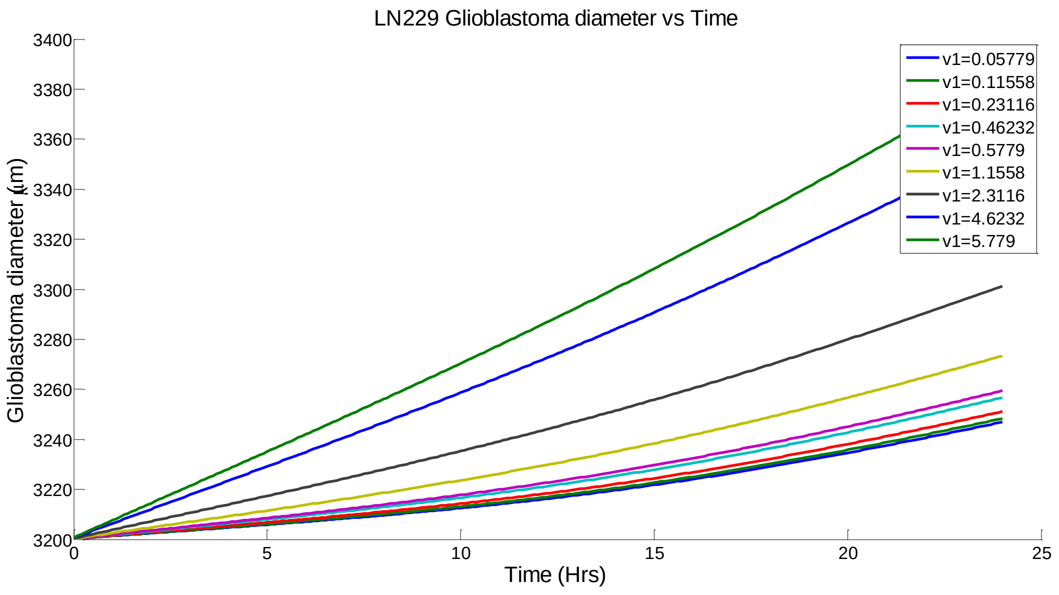
LN229 Glioblastoma diameter vs Time
Glioblastoma diameter (μm)
Time (Hrs)
v1=0.05779
v1=0.11558
v1=0.23116
v1=0.46232
v1=0.5779
v1=1.1558
v1=2.3116
v1=4.6232
v1=5.779

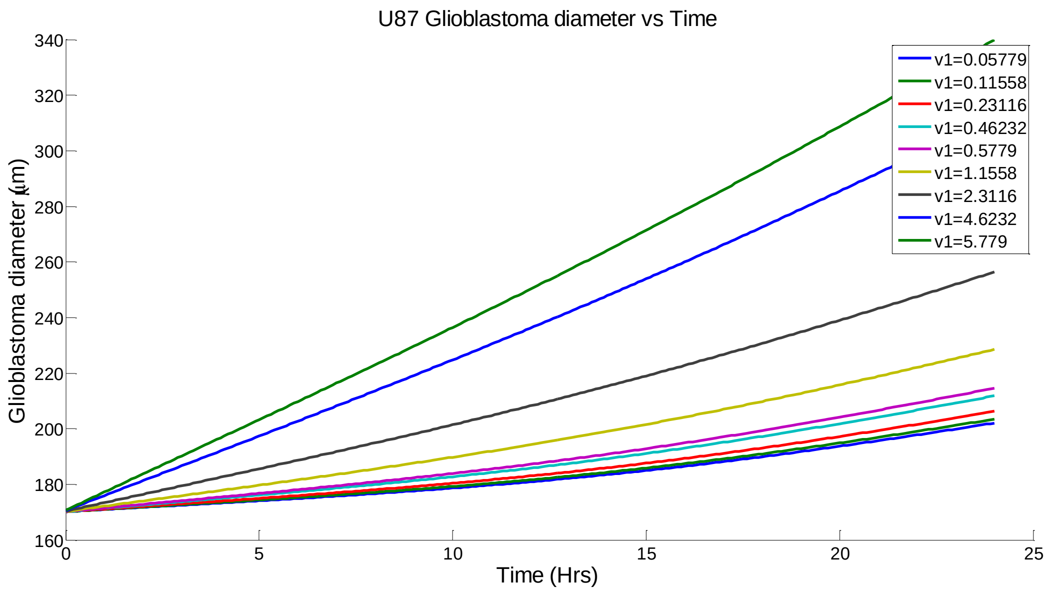
U87 Glioblastoma diameter vs Time
Glioblastoma diameter (μm)
Time (Hrs)
v1=0.05779
v1=0.11558
v1=0.23116
v1=0.46232
v1=0.5779
v1=1.1558
v1=2.3116
v1=4.6232
v1=5.779

k11

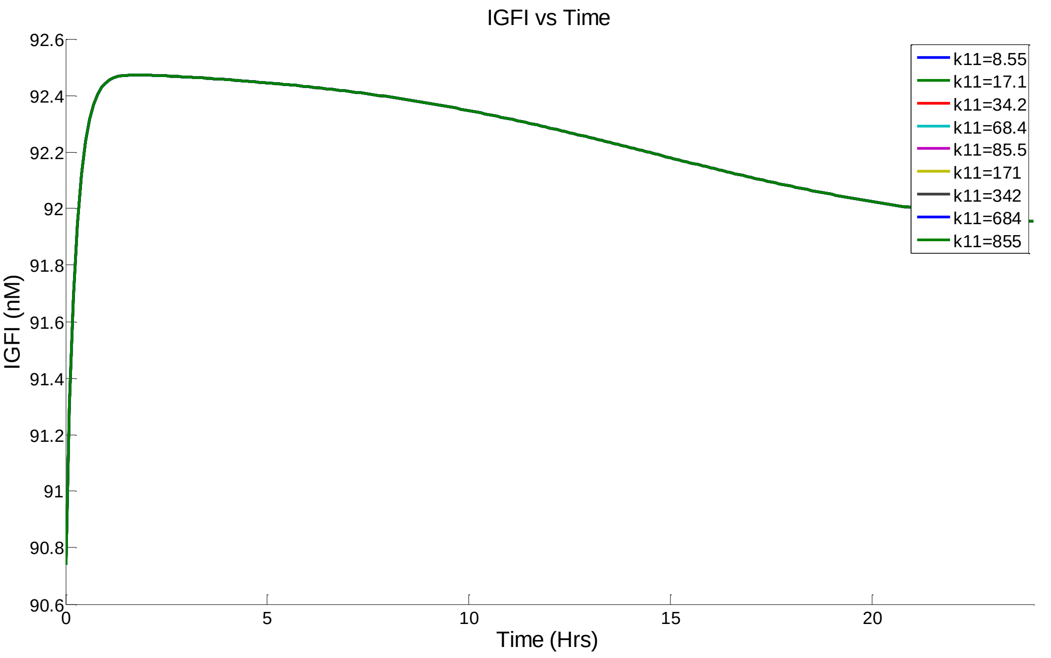
IGFI vs Time
IGFI (nM)
Time (Hrs)
k11=8.55
k11=17.1
k11=34.2
k11=68.4
k11=85.5
k11=171
k11=342
k11=684
k11=855

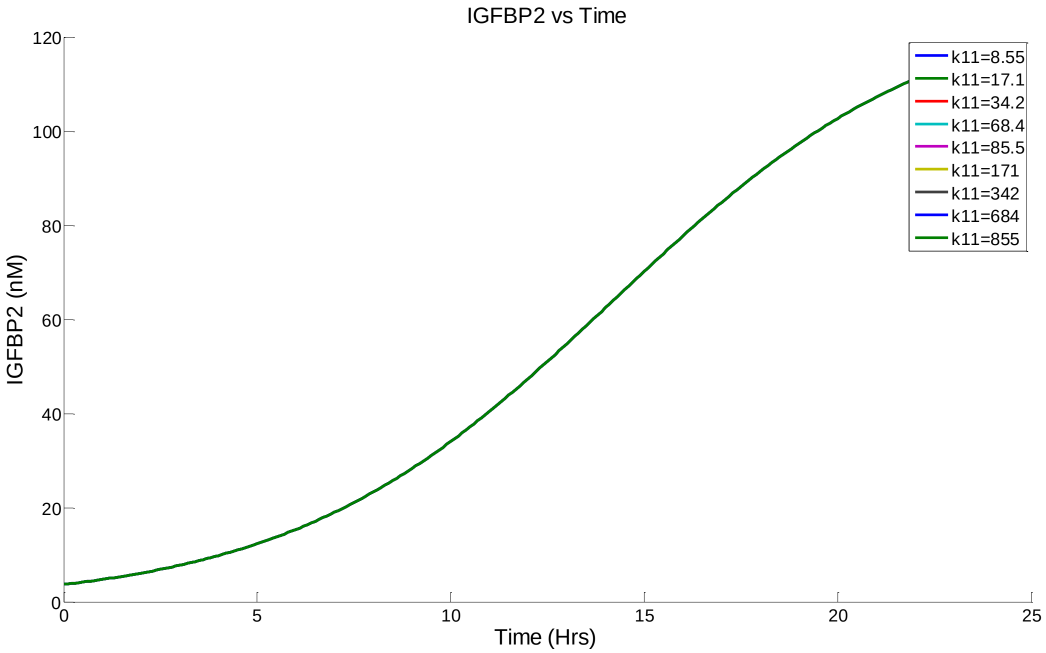
IGFBP2 vs Time
IGFBP2 (nM)
Time (Hrs)
0
20
40
60
80
100
120
0
5
10
15
20
25
k11=8.55
k11=17.1
k11=34.2
k11=68.4
k11=85.5
k11=171
k11=342
k11=684
k11=855

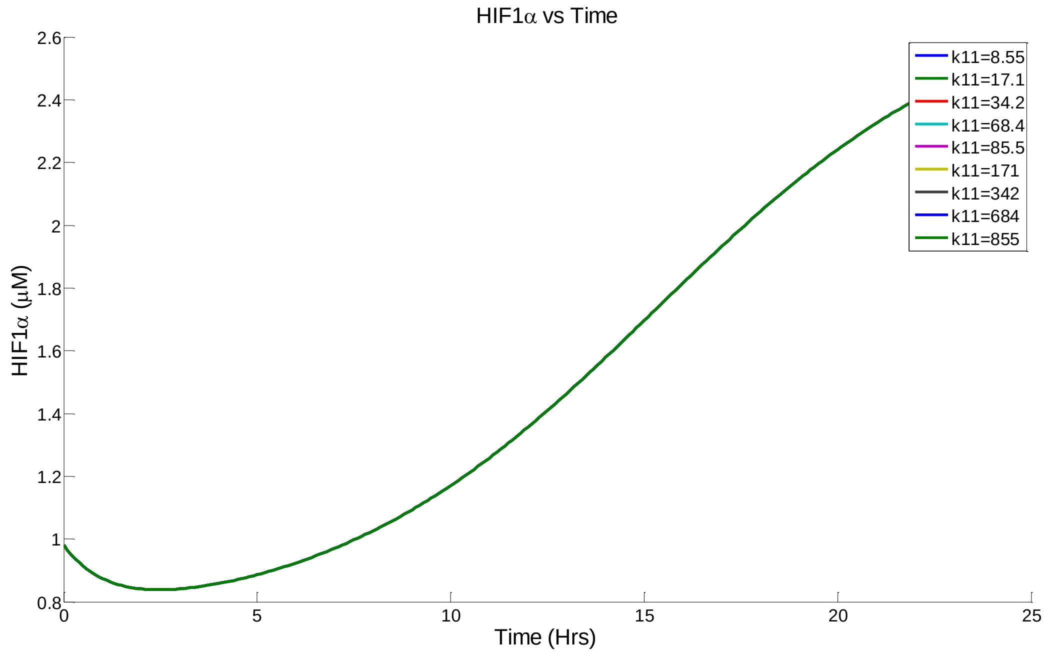
HIF1α vs Time
HIF1α (μM)
Time (Hrs)
0.8
1
1.2
1.4
1.6
1.8
2
2.2
2.4
2.6
0
5
10
15
20
25
k11=8.55
k11=17.1
k11=34.2
k11=68.4
k11=85.5
k11=171
k11=342
k11=684
k11=855

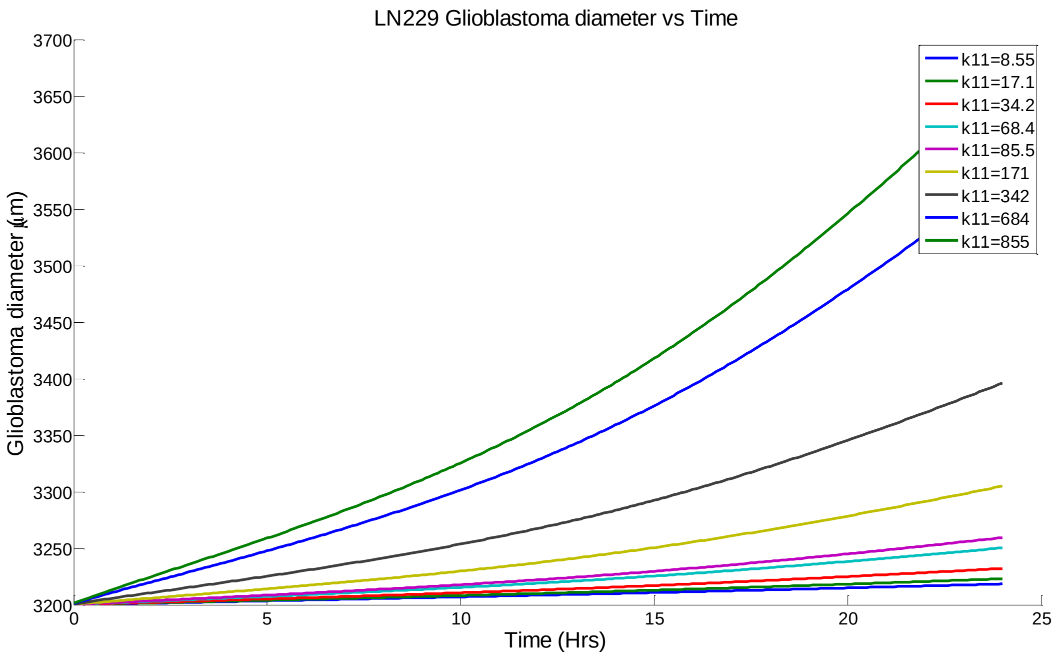
LN229 Glioblastoma diameter vs Time
Glioblastoma diameter (μm)
Time (Hrs)
3200
3250
3300
3350
3400
3450
3500
3550
3600
3650
3700
0
5
10
15
20
25
k11=8.55
k11=17.1
k11=34.2
k11=68.4
k11=85.5
k11=171
k11=342
k11=684
k11=855

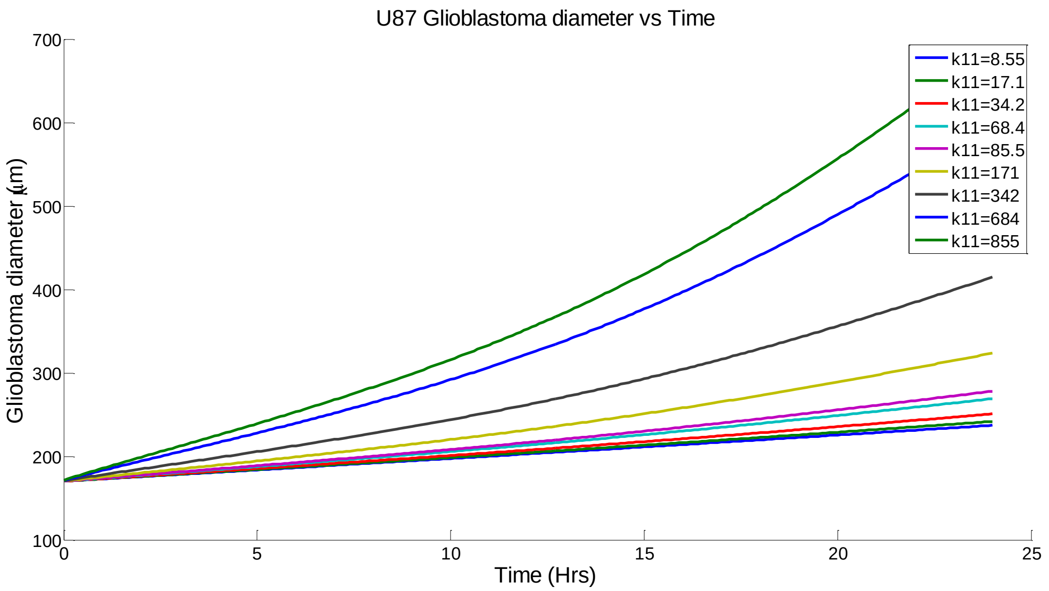
U87 Glioblastoma diameter vs Time
Glioblastoma diameter (μm)
Time (Hrs)
100
200
300
400
500
600
700
0
5
10
15
20
25
k11=8.55
k11=17.1
k11=34.2
k11=68.4
k11=85.5
k11=171
k11=342
k11=684
k11=855
