# Supplementary material for: Simulation Predicts IGFBP2-HIF1α Interaction Drives Glioblastoma Growth
Source: PLoS Comput Biol. 2015 Apr 17;11(4):e1004169. doi: 10.1371/journal.pcbi.1004169 (PMC4401766; doi:10.1371/journal.pcbi.1004169)
Supplement: S1 Fig — PC1 is the first principle component and PC2 is the second principle component. Both components contributed about 10% each to the overall correlation. (PDF) [file pcbi.1004169.s003.pdf]

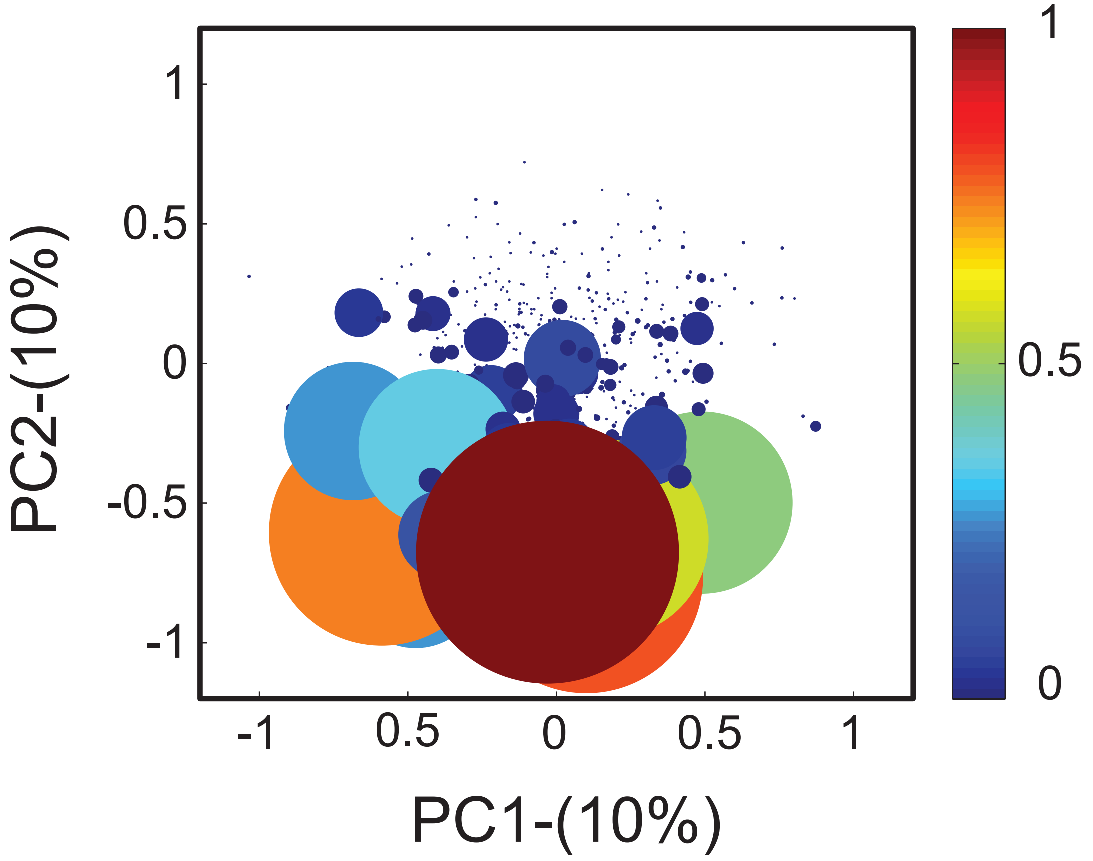

S1 Figure. Results from Principle Component Analysis of the rate constants and its effect on the glioblastoma growth in the U87 glioblastoma cell line. PC1 is the first principle component and PC2 is the second principle component. Both components contributed about 10% each to the overall correlation.
