# Supplementary material for: Simulation Predicts IGFBP2-HIF1α Interaction Drives Glioblastoma Growth
Source: PLoS Comput Biol. 2015 Apr 17;11(4):e1004169. doi: 10.1371/journal.pcbi.1004169 (PMC4401766; doi:10.1371/journal.pcbi.1004169)
Supplement: S4 Fig — PC2 is the second principle component and PC3 is the third principle component. Both components contributed about 10% and 9% each to the overall correlation respectively. (PDF) [file pcbi.1004169.s006.pdf]

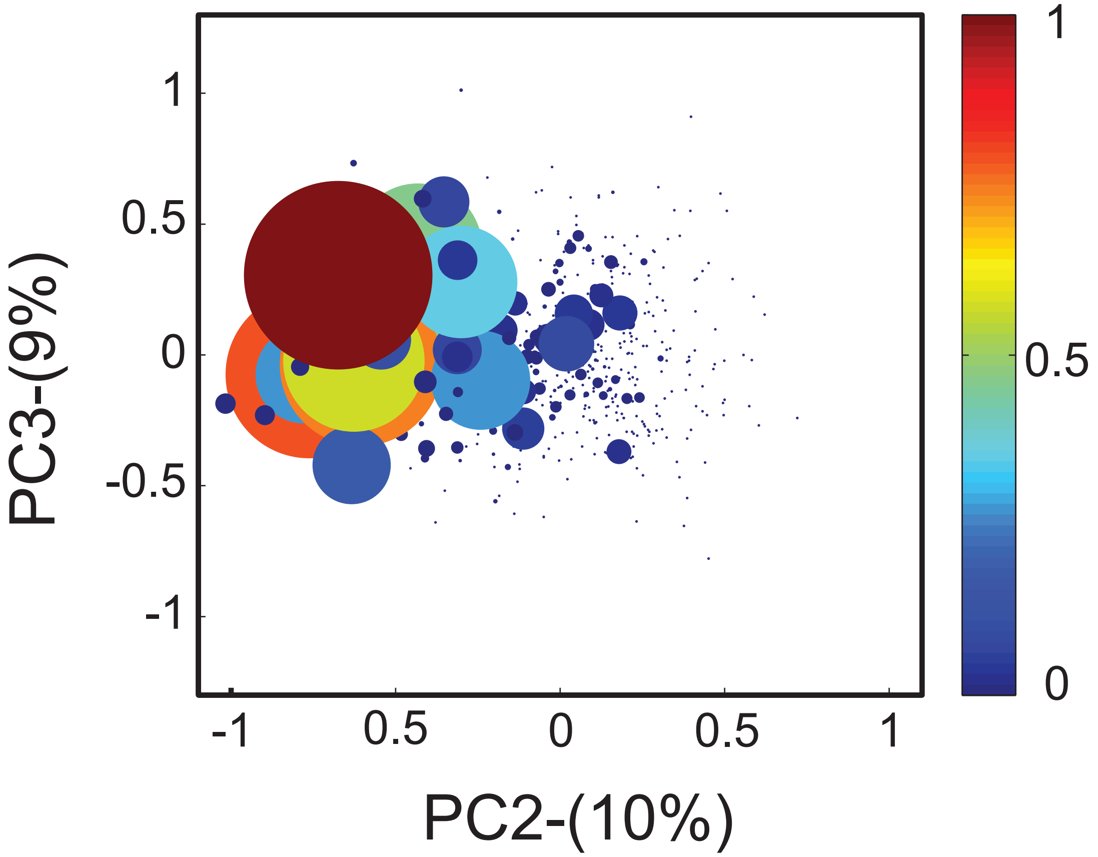

S4 Figure. Results from Principle Component Analysis of the rate constants and its effect on the glioblastoma growth in the LN229 glioblastoma cell line. PC2 is the second principle component and PC3 is the third principle component. Both components contributed about 10% and 9% each to the overall correlation respectively.
