# Supplementary material for: Simulation Predicts IGFBP2-HIF1α Interaction Drives Glioblastoma Growth
Source: PLoS Comput Biol. 2015 Apr 17;11(4):e1004169. doi: 10.1371/journal.pcbi.1004169 (PMC4401766; doi:10.1371/journal.pcbi.1004169)
Supplement: S5 Fig — Glioblastoma growth was simulated for (A) control conditions, and when two separate interactions were removed from the model: (B) IGFI to HIF1α and (C) IGFBP2 to HIF1α. (D) Removal of the IGFBP2 to HIF1α had a small reduction in the glioblastoma growth as compared to control conditions. (PDF) [file pcbi.1004169.s007.pdf]

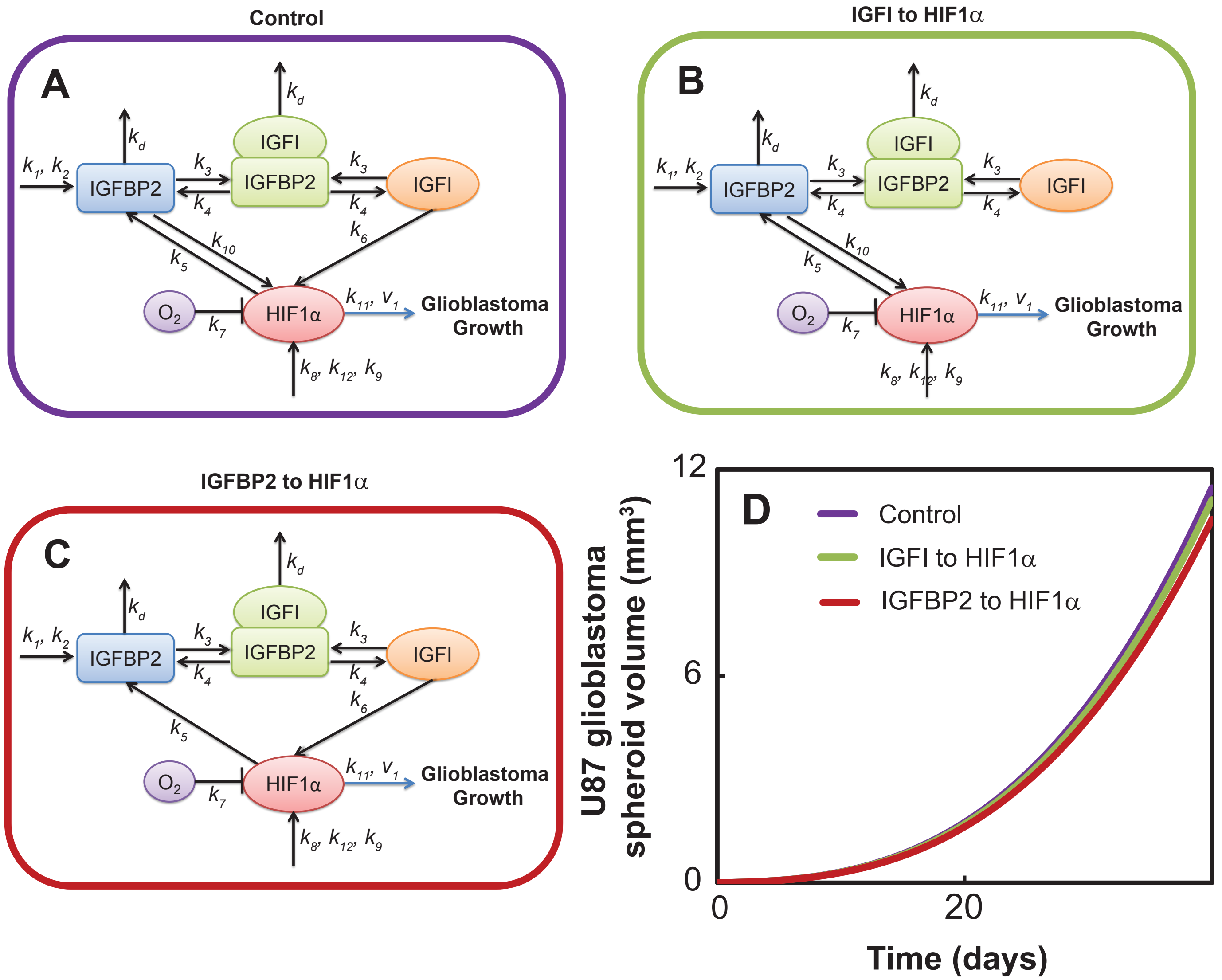

S5 Figure. *in silico* reduction of glioblastoma growth for U87 glioblastoma cell line. Glioblastoma growth was simulated for (A) control conditions, and when two separate interactions were removed from the model: (B) IGFI to HIF1α and (C) IGFBP2 to HIF1α. (D) Removal of the IGFBP2 to HIF1α had a small reduction in the glioblastoma growth as compared to control conditions.
